# Mild, visible light-mediated decarboxylation of aryl carboxylic acids to access aryl radicals†
†Electronic supplementary information (ESI) available. See DOI: 10.1039/c6sc05533h



**DOI:** 10.1039/c6sc05533h

**Published:** 2017-02-27

**Authors:** L. Candish, M. Freitag, T. Gensch, F. Glorius

**Affiliations:** a Organisch-Chemisches Institut , Westfälische Wilhelms-Universität Münster , Corrensstrasse 40 , 48149 , Münster , Germany . Email: glorius@uni-muenster.de

## Abstract

Herein we present the first example of aryl radical formation *via* the visible light-mediated decarboxylation of aryl carboxylic acids using photoredox catalysis. This method constitutes a mild protocol for the decarboxylation of cheap and abundant aryl carboxylic acids and tolerates both electron-rich substrates and those lacking *ortho*-substitution. The *in situ* formation of an acyl hypobromite is proposed to prevent unproductive hydrogen atom abstraction and trapping of the intermediate aroyloxy radical, enabling mild decarboxylation.

## Abstract 2

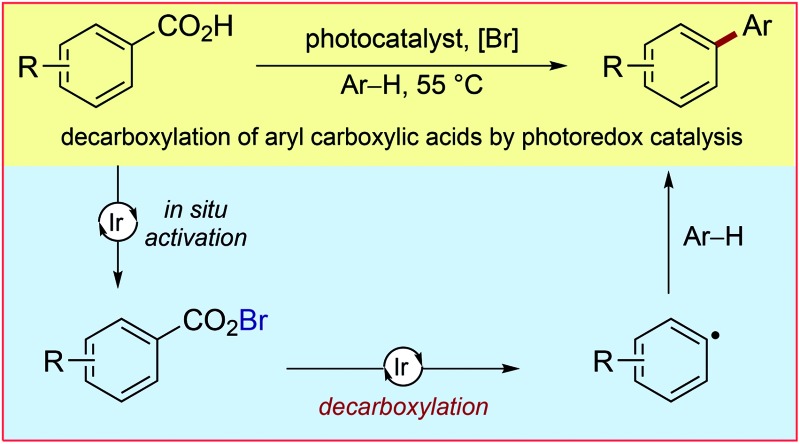
Herein we present the first example of aryl radical formation *via* the visible light-mediated decarboxylation of aryl carboxylic acids using photoredox catalysis.

## 

Since seminal publications by Lei,^1*a*^ Nishibayashi^1*b*^ and MacMillan,^1*c*^ the decarboxylation of alkyl carboxylic acids *via* photoredox catalysis^2^ has become a commanding method to generate alkyl radicals^1*d*^ which can undergo either direct functionalisation or participate in dual-catalytic processes.^1*e*^ Despite strong interest in this area, the decarboxylation of aryl carboxylic acids using photoredox catalysis is yet to be realised. While aryl radicals can be accessed *via* photoredox catalysis from electron-poor arenes, such as diazonium and iodonium salts, aryl halides,^3^ and aryl borates,^4^ many of these reagents are unstable, expensive and often not commercially available. In contrast, aryl carboxylic acids are abundant and cheap, making them appealing starting materials. Considering the wealth of methods for the functionalisation of alkyl carboxylic acids using photoredox catalysis,^1^ the development of a mild protocol for the decarboxylative functionalisation of aryl carboxylic acids is of high importance.

The decarboxylation of aroyloxy radicals (**I**) is known to be challenging,^5*a*^ in fact, Barton categorised **I** as a “non-decarboxylating acyloxy radical”, noting that **I** does not decarboxylate at temperatures below 120–130 °C.^5b,c^ Though generation of **I** *via* photoredox catalysis has been described, either *via* single electron reduction of benzoyl peroxide^6*a*^ or single electron oxidation of benzoates,^6b,7^ decarboxylation was not reported in these cases. Instead, **I** has been reported to add to arenes to provide benzoate esters (Scheme 1a).^6^ Additionally, our laboratory recently disclosed the use of **I** as a hydrogen atom transfer (HAT) catalyst for the functionalisation of unactivated C(sp^3^)–H bonds.^7^ While the activation energy for the decarboxylation of **I** is only 8–9 kcal mol^–1^,^8*a*^ the rate of decarboxylation (*k* = 1.4 × 10^6^ s^–1^) is not competitive with nondecarboxylative pathways including addition to arenes (*k* = 2.2 × 10^8^ M^–1^ s^–1^)^8*a*^ and HAT (*k* = 1.2 × 10^7^ M^–1^ s^–1^).^8a,9^ As such, while the decarboxylation of **I** is theoretically possible its realisation is practically challenging. By comparison, the more facile decarboxylation of alkyl carboxylic acids, which readily occurs at ambient temperatures, proceeds at least one thousand times faster than the decarboxylation of **I**.^8*c*^

**Scheme 1:**
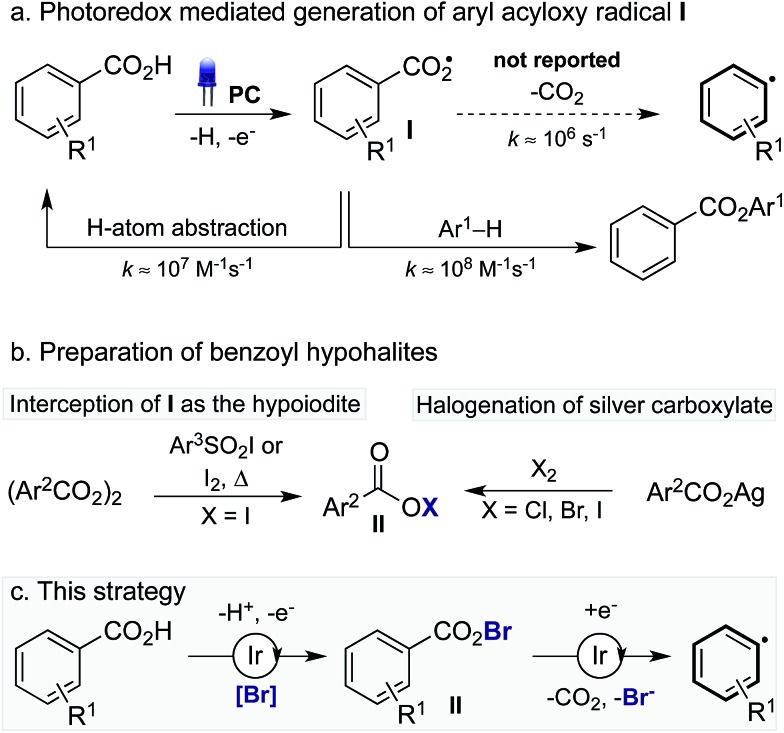
Challenges of photoredox-catalysed decarboxylation of aryl carboxylic acids and this approach to achieve a mild decarboxylation protocol.

Recently, Greaney^10a,b^ and Su^10*c*^ disclosed their work describing the catalytic oxidative radical decarboxylation of aryl carboxylic acids using Ag(i) and a stoichiometric oxidant. This work is complementary to the aryl decarboxylation protocols developed by Goossen and Larrosa involving CO_2_ extrusion/arene metalation,^11^ however, characteristic of all methods for aryl decarboxylation is the requirement of forcing reaction conditions. Given photoredox catalysis has become a commanding method to generate radicals under mild reaction conditions^2^ we believed we could harness this potential in order to develop a mild process for the decarboxylation of aryl carboxylic acids. To this end, we were inspired by reports from Hammond^12*a*^ and da Silva Corrêa^12*b*^ that aroyloxy radicals, formed *via* the thermal decomposition of benzoyl peroxide, react rapidly with iodination reagents to yield benzoyl hypoiodites, such as **II** (X = I) (Scheme 1b). Acyl hypohalites are also proposed intermediates in the Hunsdiecker reaction, formed *via* the halogenation of stoichiometric metal carboxylates (M = Ag, Hg, Tl), with heating of the benzoyl hypohalite initiating a radical chain that furnishes the halogenated product.^13,14^ We proposed that generation of an aroyloxy radical *via* photoredox catalysis in the presence of a suitable bromination reagent may enable the *in situ* generation of benzoyl hypobromite **II** (X = Br), thus inhibiting undesired reactions of **I** including HAT and addition to arenes (Scheme 1c). It was hoped that a subsequent single electron reduction of **II** would facilitate decarboxylation, thus yielding an aryl radical, which could be trapped with arenes to furnish biaryl products.

As the success of this process hinges on the ability of **II** to participate in single electron reduction, and for the resultant radical anion to undergo decarboxylation at temperatures unprecedented for aryl carboxylic acids, hypobromite **1** was initially synthesised and subjected to photoredox conditions. Iridium-centred photocatalyst [Ir(dF(CF_3_)ppy)_2_(dtbbpy)]PF_6_ (dF(CF_3_)ppy = 2-(2,4-difluorophenyl)-5-(trifluoromethyl)pyridine, dtbbpy = 4,4′-di-*tert*-butyl-2,2′-bipyridine) (**PC**) was selected, as luminescence quenching studies suggested it capable of oxidising benzoates, while also being significantly reducing (
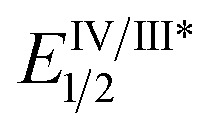
 = –0.89 V or 
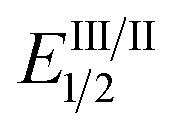
 = –1.37 V *vs.* SCE). Hypobromite **1** was prepared at low temperature and then heated to 55 °C in chlorobenzene in the absence of **PC**.^15^ As predicted, both under irradiation with blue LEDs (*λ*_max_ = 455 nm) and in the dark, only benzoate ester **2a** was formed (eqn (1)). Gratifyingly, when hypobromite **1** was irradiated at 55 °C in the presence of **PC** the desired biaryl product **3a** was afforded, along with ester **2a** (eqn (2)). This result suggests that oxidative quenching of **PC** (
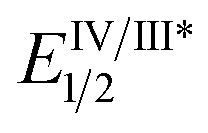
 = –0.89 V *vs.* SCE) by **1** is possible, thus providing radical anion **III** and facilitating decarboxylation. Addition of the aryl radical to the arene solvent and oxidation of the intermediary cyclohexadienyl radical by the Ir(iv) should, after deprotonation, afford biaryl **3a** with regeneration of the ground-state **PC**.1
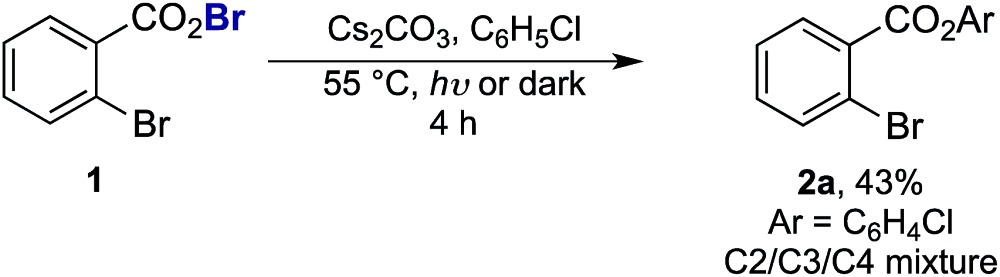

2
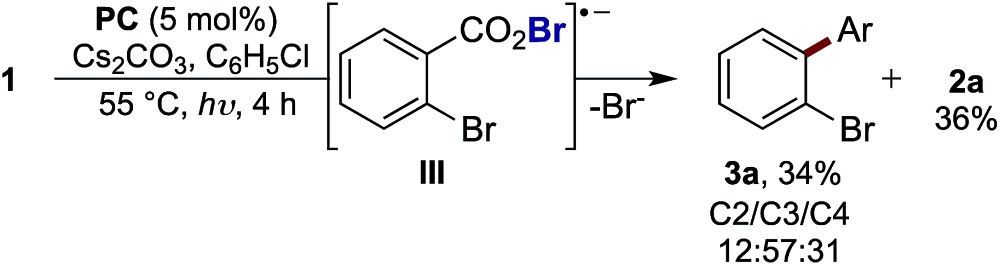



With evidence that decarboxylation of a hypobromite could be facilitated by the photocatalyst and light, we commenced studies into the photoredox catalysed decarboxylation of aryl carboxylic acid **4a**. The irradiation of **4a** under visible light at 55 °C with **PC** (3 mol%) and Cs_2_CO_3_ (2 equiv.) in the absence of bromine resulted in only 1% yield of biaryl **3b** (Chart 1). However, the addition of bromine (3.5 equiv.) to the reaction enabled the product **3b** to form in 51% yield (Chart 1), along with approximately 10% of corresponding benzoate ester **2b**. When this reaction was performed in the absence of **PC**, a mixture of mono-, di-, tri- and tetra-bromobenzene was observed,^16^ along with 14% **2b**. As bromine is a toxic, volatile liquid, incompatible with numerous functional groups, we decided to explored alternative brominating agents to determine whether they could also affect the reaction. Pleasingly, we found many brominating reagents capable of facilitating product formation (see ESI† for a list of the halogenation reagents screened). In line with our previous studies, bromomalonate derivatives were found to be excellent reagents,^1h,17^ with **5** providing the product in 77% yield (with no benzoate ester observed), while *N*-bromosuccinimide (NBS) and *N*-bromophthalimide (NBP) provided the product in lower yields (Chart 1).

**Chart 1:**
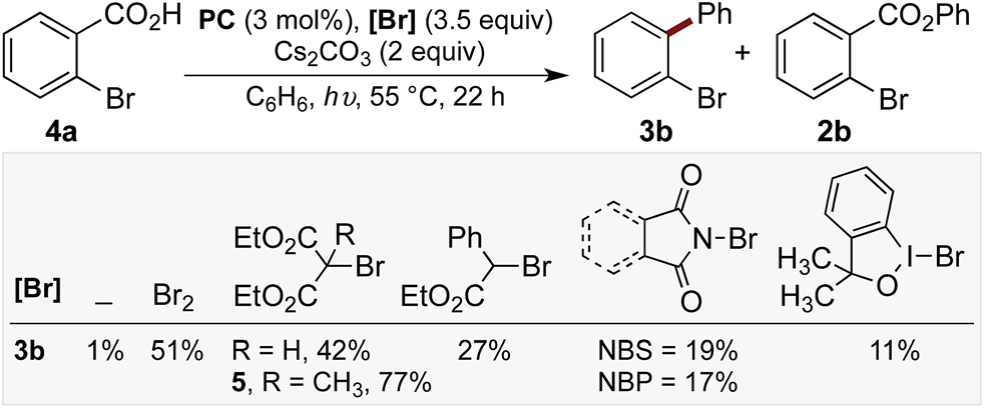
Screen of different bromination reagents. General reaction conditions: reactions were performed using **4a** (0.1 mmol), **PC** (3 mol%), Cs_2_CO_3_ (2 equiv.), [**Br**] (3.5 equiv.), arene (2.2 mL), blue LED (*λ*_max_ = 455 nm), 55 °C, 22 h. Yields determined by GC-MS using decane as standard.

To gain a deeper understanding of the decarboxylation, the influence of the sterics and electronics of the aryl carboxylate and the electronics of the trapping arene were studied (Scheme 2). The decarboxylation of electron-deficient acids with *ortho*-substituents proceeded smoothly at 55 °C to provide biaryls (**3b–f**). The reaction of *ortho*-bromobenzoic acid (**4b**) was performed on 1.5 mmol scale, though an extended reaction time (60 h) was required.

**Scheme 2:**
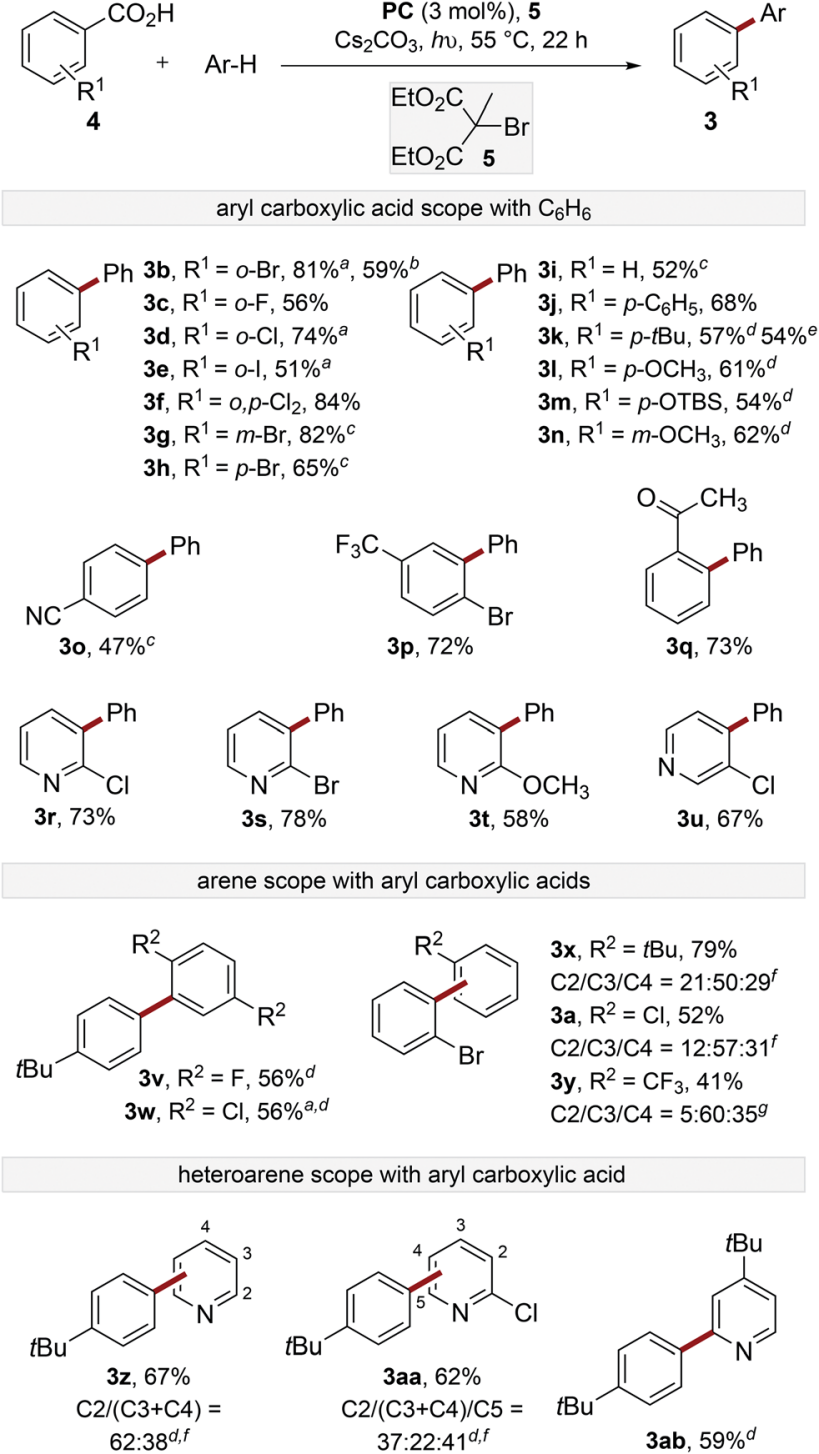
Scope of the biaryl synthesis. General reactions conditions; benzoic acid (0.2 mmol), **PC** (3 mol%), Cs_2_CO_3_ (2 equiv.), **5** (3.5 equiv.), arene (4.5 mL), blue LED (*λ*_max_ = 455 nm), 55 °C, 22 h. Isolated yields (average of two reactions) following column chromatography. ^*a*^Reactions were performed with arene (150 equiv.) in CH_3_CN (1 : 1 v/v). ^*b*^Reactions were performed on 1.5 mmol scale, reaction time = 60 h. ^*c*^Reactions were performed with blue LED (*λ*_max_ = 415 nm) at 80 °C. ^*d*^2 equiv. **5** used. ^*e*^Reactions were performed using 23 W CFL. ^*f*^Ratio of isomers determined by GC-MS. ^*g*^Ratio of isomers determined by ^19^F NMR spectroscopic analysis of the crude mixture.

The reaction of electron-deficient acids containing *meta*- or *para*-substituents required heating the reaction to 80 °C to achieve acceptable yields of the products (**3g–h**). Acids containing electron-donating substituents are generally not tolerated in the catalytic oxidative radical decarboxylation reactions described by Greaney and Su,^10b,c^ presumably because of their slower rate of decarboxylation.^18^ Interestingly, electron-rich acids were found to react smoothly under our reaction conditions to afford products **3j–n**. A small amount of decarboxylative bromination was observed for electron-rich benzoic acids, although this could be suppressed by reducing the equivalents of **5**, though lower yields of the biaryl products were observed. Pleasingly, the decarboxylation of *para-tert*-butylbenzoic acid proceeded smoothly, providing the product (**3k**) in similar yield (54% *cf.* 57%) using a 23 W CFL (compact fluorescent lamp) instead of blue LEDs. Substituted nicotinic and isonicotinic acids also underwent decarboxylative arylation in reasonable yields to provide phenylpyridines (**3r–u**).

The scope of the trapping arene was also explored, with 1,4-disubstituted arenes affording single biaryl products (**3v–w**), while electron-rich and electron-deficient monosubstituted arenes act as efficient radical traps to provide the coupled products as a mixture of isomers (**3a**, **3x–y**). Additionally, electron-deficient heteroarenes also provided satisfactory yields of phenylpyridine products (**3z**, **3aa–ab**).^19^ In all cases, the arene was used in excess. However, filtration of the crude reaction mixture through a short silica gel plug followed by distillation generally allowed greater than 80% of the remaining arene to be reisolated in high purity.

In order to gain insight into the reaction mechanism we studied the kinetics of the excited **PC** *via* Stern–Volmer luminescence quenching. On the basis of these results we propose that reductive quenching of **PC** (Ir(iii)*/Ir(ii)) by the benzoate anion provides aroyloxy radical **I** (Scheme 3). Two alternative pathways were then considered for the decarboxylation of **I** to provide the aryl radical. Pathway A involves the direct decarboxylation of **I**, a process that is known to be energetically feasible but is unprecedented at these reaction temperatures.^5a,b,8a,20^ Alternatively, pathway B is proposed to involve bromination of **I** to furnish hypobromite **II**, thus circumventing unproductive reactions of **I** with the solvent. Based on the results of the stoichiometric experiments of **1** (*vide supra*), we propose a single electron reduction of **II** by the sufficiently reducing Ir(ii) (
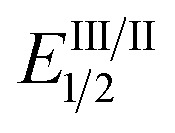
 = –1.37 V *vs.* SCE) provides radical anion **III**, which undergoes decarboxylation to afford the aryl radical. Theoretical analysis suggests that upon reduction to the radical anion **III**, stepwise bond dissociation does not take place. Instead, concerted C–C and O–Br bond cleavage occurs to yield CO_2_, a bromide anion and the phenyl radical (see the ESI† for details of computational analysis). Subsequent trapping of the aryl radical (formed *via* either pathway) with a (hetero)arene should generate the cyclohexadienyl radical **IV**, which, following oxidation to the aryl cation and deprotonation, affords the biaryl product **3**.

**Scheme 3:**
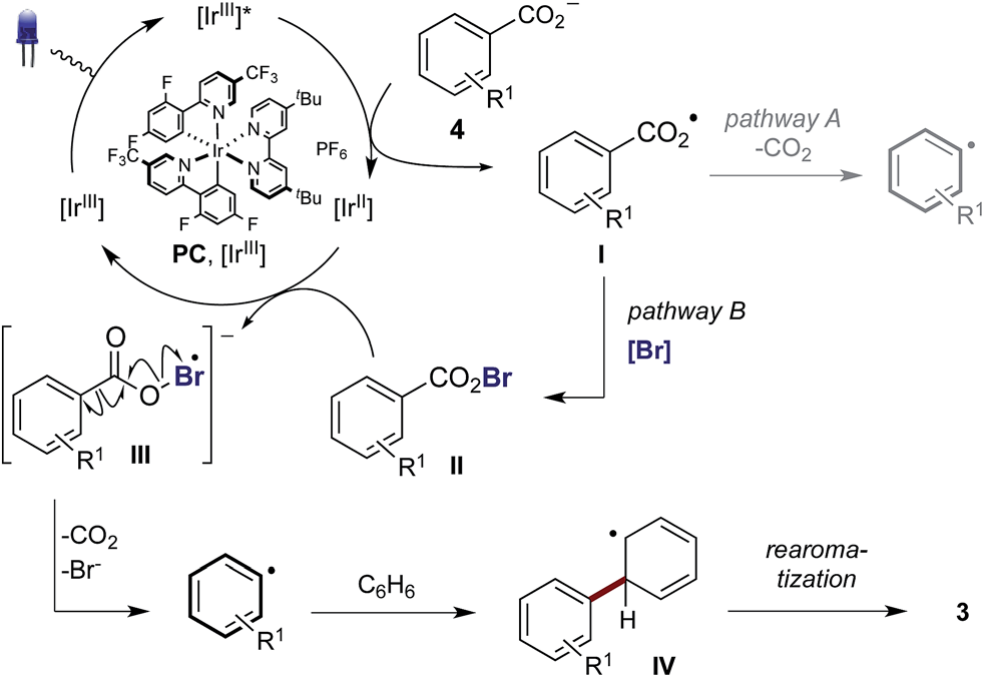
Proposed reaction mechanism. Counterions and ligand sphere of Ir are omitted for clarity.

Our mechanistic hypothesis was supported by a number of experiments. The reaction was undertaken using biphenyl-2-carboxylic acid (**4b**), thus enabling trapping of the aroyloxy radical **I** *via* 6-*endo-trig* cyclisation to form lactone **6** in 89% yield (Scheme 4a).^6*b*^ Additionally, to confirm the reaction was proceeding *via* the intermediacy of an aryl radical, the selectivity of the reaction of 4-methoxybenzoic acid with fluorobenzene was analysed and compared to the reported selectivity for the addition of 4-methoxyphenyl radical to fluorobenzene (Scheme 4b).^21^ The observation of identical regioselectivity for the biaryl product **3ac** afforded to that reported suggests the reaction is proceeding *via* the same reactive species.

**Scheme 4:**
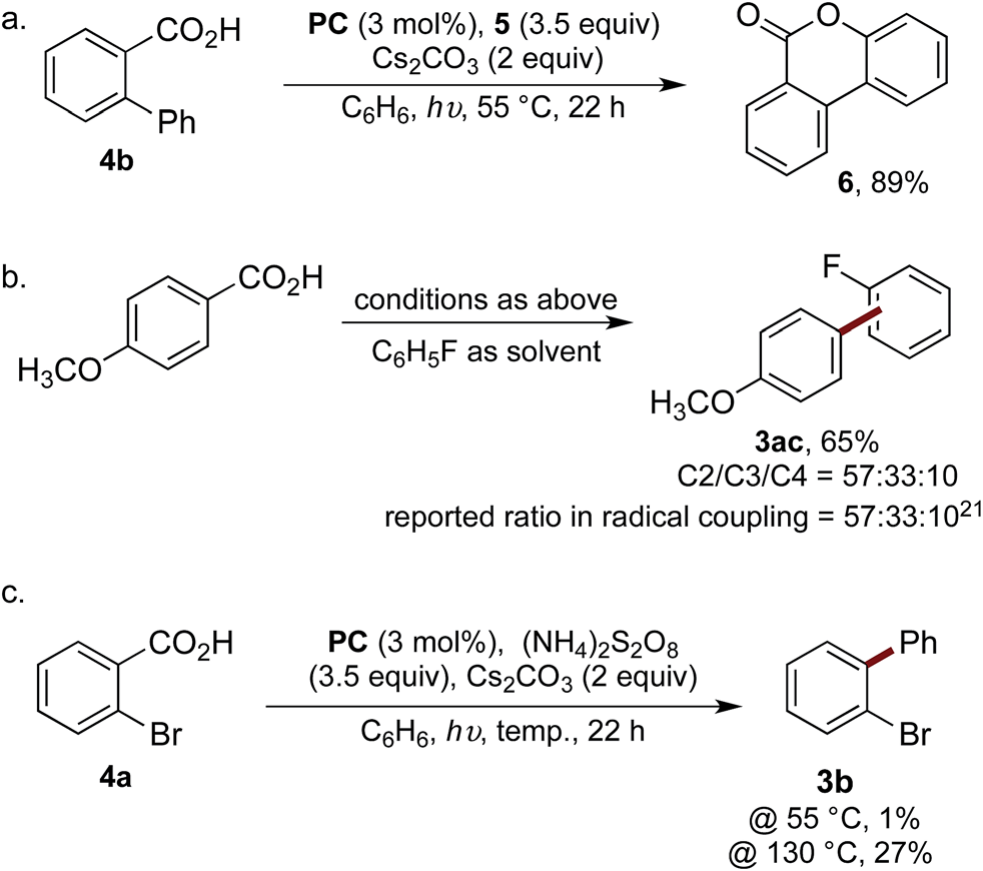
(a) Trapping of the intermediate aroyloxy radical; (b) comparison of selectivities for biaryl formation, ratio determined by ^19^F NMR spectroscopic analysis; (c) visible light-mediated decarboxylation of **4a** at elevated temperatures.

We next attempted to determine whether the reaction proceeds *via* direct decarboxylation of **I** (pathway A), or if pathway B is more likely. Though the decarboxylation of aroyloxy radicals are unprecedented at 55 °C, we were curious whether the bromination reagents were merely acting as oxidants for **PC**. Thus, the reaction of **4a** was performed under the standard reaction conditions, in the absence of **5**, which was replaced with a number of reagents known to be capable of oxidising **PC**. Under these conditions, biaryl **3b** was either not formed or only observed in trace amounts (<5%) (see ESI† for details). Additionally, to test Barton's theory, that aroyloxy radicals require >120–130 °C to undergo decarboxylation (*vide supra*), the reaction of **4a** was undertaken at 130 °C in the presence of ammonium persulfate. Biaryl **3b** was afforded in 27%, *cf.* 1% when the reaction was performed at 55 °C (Scheme 4c). Finally, when the reaction of biphenyl-2-carboxylic acid (**4b**) was undertaken with ammonium persulfate, instead of **5**, lactone **6** was isolated in 34% yield.^6*b*^ This confirms that ammonium persulfate is a competent oxidant under the reaction conditions at 55 °C. Taken together, we believe that this provides strong experimental evidence, which is in-line with literature precedence, that the reaction is not proceeding *via* the direct decarboxylation of aroyloxy radical **I** (pathway A). Based on the results of these mechanistic studies and the stoichiometric experiments of hypobromite **1** (eqn (1) and (2)) we tentatively propose that pathway B is operative under our reaction conditions. Experimental studies and detailed computational analysis are currently on going to gain deeper insight into the reaction mechanism.

## Conclusions

In summary, the first decarboxylation of aryl carboxylic acids to provide aryl radicals *via* photoredox catalysis is disclosed. Moreover, we have developed a method for the relatively mild decarboxylation of aryl carboxylic acids, proceeding at much lower temperatures than methods previously reported. The reaction does not require *ortho*-substituents on the aryl carboxylic acid, and, unlike previously reported catalytic oxidative decarboxylative protocols, tolerates electron-rich acids. It is proposed that the key to achieving this decarboxylation is the *in situ* formation of a benzoyl hypobromite. We strongly believe that this work paves the way for the development of mild protocols for the decarboxylative functionalisation of cheap, abundant aryl carboxylic acids.
