# Supplementary material for: Mild, visible light-mediated decarboxylation of aryl carboxylic acids to access aryl radicals
Source: Chem Sci. 2017 Feb 27;8(5):3618–22. doi: 10.1039/c6sc05533h (PMC5437376; doi:10.1039/c6sc05533h)
Supplement: Supplementary file 1 [file SC-008-C6SC05533H-s001.pdf]

Electronic Supplementary Material (ESI) for Chemical Science.
This journal is © The Royal Society of Chemistry 2017

Mild, visible light-mediated decarboxylation of aryl carboxylic acids to access aryl radicals

Lisa Candish, Matthias Freitag, Tobias Gensch, Frank Glorius*

Organisch-Chemisches Institut, Westfälische Wilhelms-Universität Münster, Corrensstrasse 40, 48149 Münster

Supporting Information

Contents

1. **General information** S2
2. **Synthesis of carboxylic acids** S3
3. **Stoichiometric experiments with benzoyl hypobromite II** S4
4. **Optimization and control reactions** S8
5. **Oxidant screen** S9
6. **General procedure for the visible light-promoted aryl decarboxylation** S9
7. **Scope of the visible light-promoted decarboxylation/radical trapping** S10
8. **Mechanistic studies** S19
    a) **Stern-Volmer luminescence quenching experiments** S19
    b) **Quantum yield measurements** S21
    c) **Reaction rates** S22
    d) **Analysis of radical intermediates** S23
    e) **Investigation of alternative reaction pathways** S23
    f) **Qualitative detection of CO_2** S24
    g) **Theoretical computations for the decarboxylation of III** S25
9. **References** S28
10. **^{1}H NMR, ^{13}C NMR and ^{19}F NMR spectral data** S29

1. General information

Unless otherwise noted, all reactions were carried out under an atmosphere of argon in screw-capped vials. The solvents used were purified by distillation over standard drying agents and were stored over molecular sieves and transferred under argon. Blue LEDs (5 W, λ_{max} = 455 nm) were used for blue light irradiation. In each case, six reactions were setup and the light source was placed ~ 3 cm from the reaction vessels (Figure S1 A-B). A box, was placed over the lights to shield the light. The temperature of the solvent in the reaction was measured using a contact thermometer to be 55 °C. Reactions conducted at 80 °C were carried-out using a Schlenk tube fitted with a glass rod, with a blue LED (λ_{max} = 415 nm) mounted atop the glass rod (Figure S1 C-D).

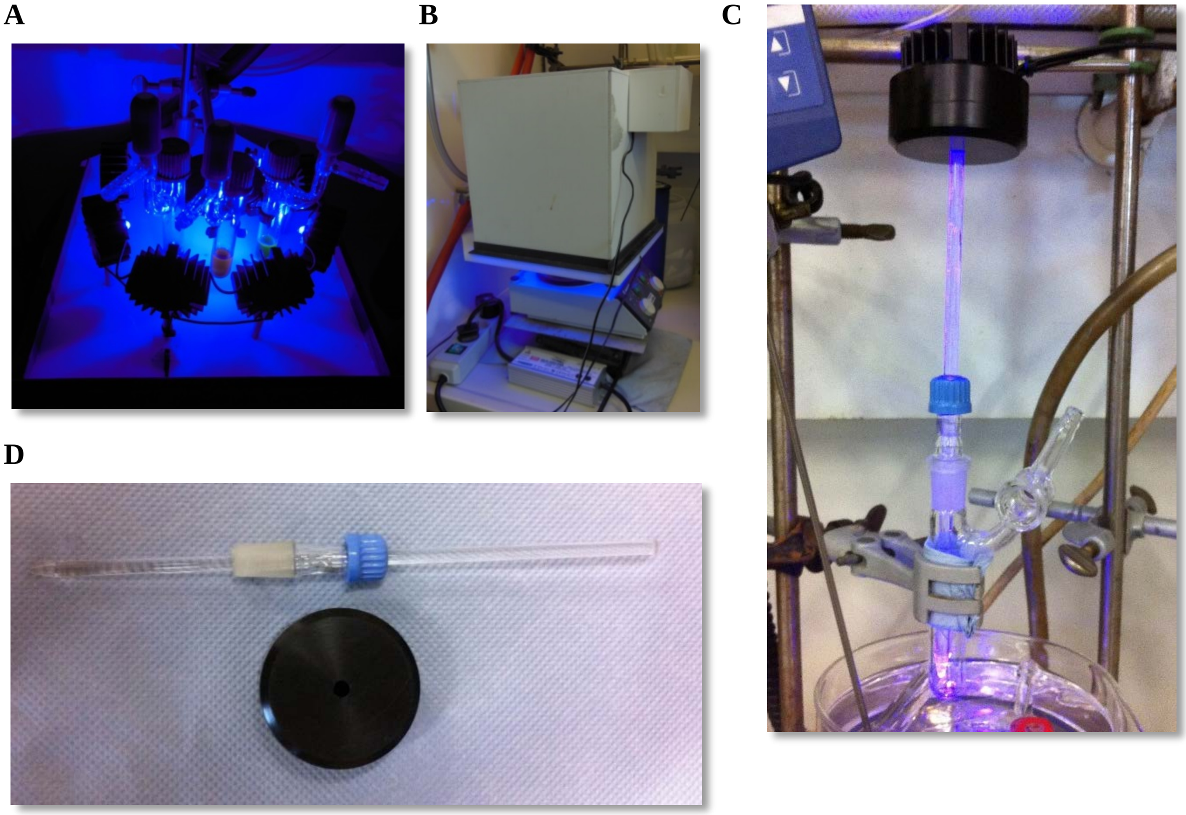

Figure S1. Photographs of; **A-B** the custom-made "light box" used for reactions conducted at 55 °C, **C** Schlenk tube setup with glass rod, LED-holder and blue LED, **D** Glass rod with quick-fit and LED-holder.

$[Ru(bpy)_3]_2(PF_6)_2$ (bpy = 2,2′-bipyridine),[1] $[Ru(bpz)_3](PF_6)_2$ (bpz = 2,2′-bipyrazine),[2] $[Ir(ppy)_2(dtbbpy)](PF_6)$ (ppy = 2-phenylpyridine, dtbbpy = 4,4′-di-*tert*-butyl-2,2′-bipyridine),[3] and $[Ir(dF(CF_3)ppy)_2(dtbbpy)](PF_6)$ (dF(CF$_3$)ppy = 2-(2,4-difluorophenyl)-5-trifluoromethylpyridine)[4] were prepared following literature procedures.

Flash chromatography was performed on Merck silica gel (40-63 mesh) using standard techniques. NMR-spectra were recorded on a Bruker ARX-300, AV-300, AV-400 MHz or a Varian 600 MHz. Chemicals shifts (δ) are quoted in ppm downfield of tetramethylsilane. The residual solvent signals were used as references for ^{1}H and ^{13}C NMR{^{1}H} spectra ($CDCl_3$: δ_H = 7.26 ppm, δ_C = 77.16 ppm; CD_3OD: δ_H = 4.87 ppm, δ_C = 49.00 ppm; DMSO-d_6: δ_H = 2.50 ppm, δ_C = 39.52 ppm).

GC-MS spectra were recorded on an Agilent Technologies 7890A GC-system with an Agilent 5975C VL MSD or an Agilent 5975 inert Mass Selective Detector (EI) and a HP-5MS column (0.25 mm x 30 m, film: 0.25 µm). ESI mass spectra were recorded on a Bruker Daltonics MicroTof spectrometer. Infrared spectra were recorded on a Varian Associates FT-IR 3100 Excalibur or on a Shimadzu FTIR 8400S spectrometer. The wave numbers (ν) of recorded IR-signals are quoted in cm^{-1}. The absorbance

values of the samples were measured placing the sample in a quartz cuvette of pathlength of 1 cm using a JASCO V-650 spectrophotometer. No attempts were made to optimize yields for the synthesis of substrates. Elemental analysis was performed using Vario EL III elementar Hanau.

Full Stern-Volmer luminescence quenching analysis was conducted using a Jasco FP-8300 spectrofluorometer. The following parameters were employed: excitation bandwidth = 5 nm, data interval = 0.2 nm, scan speed = 500 nm/min, response time = 0.2 sec. UV/Vis Absorption spectra were recorded on a Jasco V-650 spectrophotometer, equipped with a temperature control unit at 25 °C. The samples were measured in Hellma fluorescence QS quartz cuvettes (chamber volume = 1.4 mL, H × W × D = 46 mm × 12.5 mm, 12.5 mm) fitted with a PTFE stopper.

2. Synthesis of carboxylic acids

4-((*tert*-Butyldimethylsilyl)oxy)benzoic acid (S1a)[5]

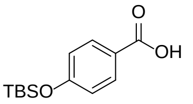

Compound **S1a** was prepared according to the procedure of Waldmann and coworkers.[5] Imidazole (1.02 g, 15.0 mmol, 1.5 equiv), 4-dimethylaminopyridine (122 mg, 1.0 mmol, 0.10 equiv) and 4-hydroxybenzoic acid (1.38 g, 10.0 mmol, 1.0 equiv) were dissolved in methylene chloride (15 ml) under argon. The solution was cooled to 0 °C and *tert*-butyldimethylsilyl chloride (3.32 g, 22.0 mmol, 2.2 equiv) in methylene chloride (5 mL) was added dropwise. The mixture was stirred at room temperature for 18 hr, then NH_4Cl (20 mL of a sat. aq. solution) was added and the solution was allowed to stir for further 15 min. The reaction mixture was extracted with ethyl acetate (3 x 20 mL) and the combined organic layers were washed with brine (50 mL), dried over $MgSO_4$, filtered and concentrated. The residue was purified by flash chromatography (CH_2Cl_2/CH_3OH 95:5 to CH_2Cl_2/CH_3OH 90:10; 0.2 vol% acetic acid). The product **S1a** was obtained as a white solid (1.52 g, 60%). All data was consistent with that previously reported.[5]

R_f (CH_2Cl_2/CH_3OH 90:10): 0.60; **^{1}H NMR** (300 MHz, CD_3OD): δ (ppm): 7.93 (AA′BB′, J = 8.8, 2H), 6.90 (AA′BB′, J = 8.8, 2H), 0.96 (s, 9H), 0.20 (s, 6H); **^{13}C NMR** (75 MHz, CD_3OD): δ (ppm): 169.7, 161.4, 132.8, 125.0, 120.9, 26.1, 19.1, –4.4.

2-Bromonicotinic acid (S1b)[6]

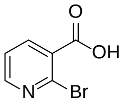

Compound **S1b** was prepared according to the procedure of Spivey and coworkers.[6] To a stirred suspension of 2-bromo-3-methylpyridin (1.00 g, 6.00 mmol, 1.0 equiv) in H_2O (25 mL) was added $KMnO_4$ (2.40 g, 15.0 mmol, 25.0 equiv) at room temperature. The resulting solution was refluxed for 20 h. The solution was allowed to cool to room temperature and filtered. The clear filtrate was concentrated to approximately 20 mL *in vacuo* and the solution was acidified to pH 3 with HCl (1M aq. solution). The resulting white precipitate was collected by filtration and dried *in vacuo*. The product **S1b** was obtained as a white solid (0.60 g, 46%). All data was consistent with that previously reported.[6]

^{1}H NMR (300 MHz, DMSO-*d6*): δ (ppm): 13.80 (s, 1H), 8.50 (dd, *J* = 4.8, 2.0 Hz, 1H), 8.13 (dd, *J* = 7.6, 2.0 Hz, 1H), 7.55 (dd, *J* = 7.6, 4.8 Hz, 1H); **^{13}C NMR** (75 MHz, DMSO-*d6*): δ (ppm): 166.5, 151.9, 139.3, 138.8, 131.2, 123.3.

2-Methoxynicotinic acid (S1c)[7]

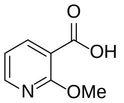

Compound **S1c** was prepared according to the modified procedure of Eliott and Goddard.[7] To a stirred suspension of 2-chloronicotinic acid (1.59 g, 10.0 mmol, 1.0 equiv) in methanol (18 mL) was added sodium methoxide (1.16 g, 21.4 mmol, 2.1 equiv) at room temperature. The resulting solution was heated at 115 °C overnight. The cooled solution was filtered and

the solid residue was washed with methanol. The solvent was removed *in vacuo* and the resulting white solid was dissolved in H_2O. The solution was acidified to pH 3 with HCl (6M aq. solution). The resulting white precipitate was collected by filtration and dried *in vacuo*. The product **S1c** was obtained as a white solid (1.01 g, 66%). All data was consistent with that previously reported.[7]

^{1}H NMR (400 MHz, DMSO-*d6*): δ (ppm): 12.95 (s, 1H), 8.33 (dd, *J* = 4.9, 2.0 Hz, 1H), 8.09 (dd, *J* = 7.5, 2.0 Hz, 1H), 7.07 (dd, *J* = 7.5, 4.9 Hz, 1H). 3.90 (s, 3H); **^{13}C NMR** (101 MHz, DMSO-*d6*): δ (ppm): 166.0, 161.4, 150.2, 140.7, 116.7, 115.1, 53.5.

3. Stoichiometric experiments with benzoyl hypobromite II

To determine whether benzoyl hypobromite **II** is a competent intermediate in the reaction, **II** was synthesized independently and subjected to the reaction conditions. The procedure described by Baran and co-workers for preparation of acetyl hypobromite[8] was selected for the synthesis, and involved the reaction of a silver benzoate with bromine at 0 °C in d_2-methylene chloride or chlorobenzene. d_2-Methylene chloride and chlorobenzene were selected as the silver carboxylate and silver bromide are insoluble in these solvents, allowing them to be easily filtered away from the solution of hypobromite at the end of the reaction.

Benzoyl hypobromite species have been reported as reactive intermediates in a number of transformations, however this species has never been characterized. Often, the reported conditions either generate a lot of byproducts, making them unsuitable to analyze the intermediate, or the acyl hypobromite is not stable under the described conditions. However, while acetyl hypobromite is known to be unstable, decomposing slowly at -20 °C in the absence of light, we believe the benzoyl hypobromite should be considerably more stable, as it cannot decompose via a similar radical chain process at ambient temperatures.

Preparation and characterization of ^{13}C labeled benzoyl hypobromite

Studies commenced with the preparation of benzoyl hypobromite to see if we could characterize this reactive species by NMR. Unfortunately, it was found to decompose too rapidly to measure a ^{13}C NMR spectrum where the carbonyl signal was visible. To overcome this, ^{13}C-labeled benzoyl hypobromite was synthesized and the ^{1}H and ^{13}C NMR measured.

To this end, silver benzoate **S2a** was first synthesized in two-steps from benzoic acid-α-^{13}C **S1d** (99 atom % ^{13}C), from the sodium salt (eq. 1): To a stirred suspension of Na_2CO_3 (239 mg, 2.25 mmol, 1.0 equiv) in methanol (5 mL) was added a solution of acid **S1d** (554 mg, 4.5 mmol, 2.0 equiv) in methanol (10 mL). The mixture was stirred for 12 hours at room temperature. After this time, the methanol was removed to quantitatively yield a white solid and the compound was dried under vacuum for 4 hours.

Following the modified procedure of Úbeda and co-workers,[9] to a magnetically stirred solution of the sodium benzoate (432 mg, 3 mmol, 1.0 equiv) in methylene chloride (12 mL) was added a solution of silver nitrate (510 mg, 3 mmol, 1.0 equiv, freshly recrystallized) in water (6 mL). A white precipitate formed and after 30 min the solid was collected by filtration and washed with a minimal volume of cold methanol. The white solid was collected and dried at room temperature under vacuum overnight, providing silver benzoate **S2a**.

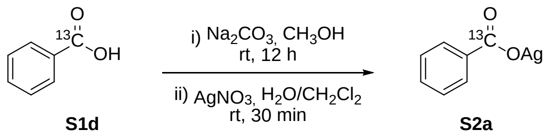

S1d S2a (1)

^{1}H NMR (400 MHz, DMSO-d_6): δ (ppm): 8.01–7.95 (m, 2H), 7.45-7.37 (m, 3H); **^{13}C NMR** (100 MHz, DMSO-d_6): δ (ppm): 170.2, 136.6 (d, *J* = 66.2 Hz), 130.3, 129.6 (d, *J* = 2.3 Hz), 127.8 (d, *J* = 4.0 Hz).

Synthesis of benzoyl hypobromite **S3**

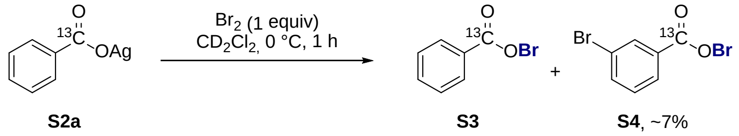

(2)

To a flame-dried Schlenk tube, foiled to protect from light, was placed ^{13}C-labeled silver benzoate (**S2a**) (115 mg, 0.5 mmol, 1.0 equiv) and d_2-methylene chloride (3 mL). The suspension was cooled to 0 °C and bromine (36 µL, 0.7 mmol, 1.4 equiv). The suspension was allowed to stir for 1 hour at 0 °C and then filtered through a syringe filter. ^{1}H and ^{13}C NMR were measured immediately, at 0 °C. Small amounts (~7%) decomposition was observed, this is believed to be **S4**.

^{1}H NMR (600 MHz, CD_2Cl_2): δ (ppm): 8.01 (ddd, *J* = 8.4, 4.0, 1.4 Hz, 2H), 7.65–7.60 (m, 1H), 7.47 (td, *J* = 7.8, 1.2 Hz, 2H); **^{13}C NMR** (151 MHz, CD_2Cl_2): δ (ppm): 167.0 (broad), 134.2 (d, J = 0.45 Hz), 130.7 (d, *J* = 2.6 Hz), 129.1 (d, *J* = 4.6 Hz), 125.

As expected, a shift up field of the signal corresponding to the carbonyl was observed (Figure S2), from 173.2 ppm to 167.0 ppm. The calculated chemical shift for this carbon of **S3** is 166 ppm.[10] The signal is also observed to broaden, with a second, small peak visible. The smaller peak is believed to correspond to the carbonyl signal of **S4**. The broad nature of the carbonyl signal of hypobromite **S3** is attributed to rotation about the oxygen-carbonyl bond of the hypobromite from the favored s-*trans* to s-*cis* conformation.

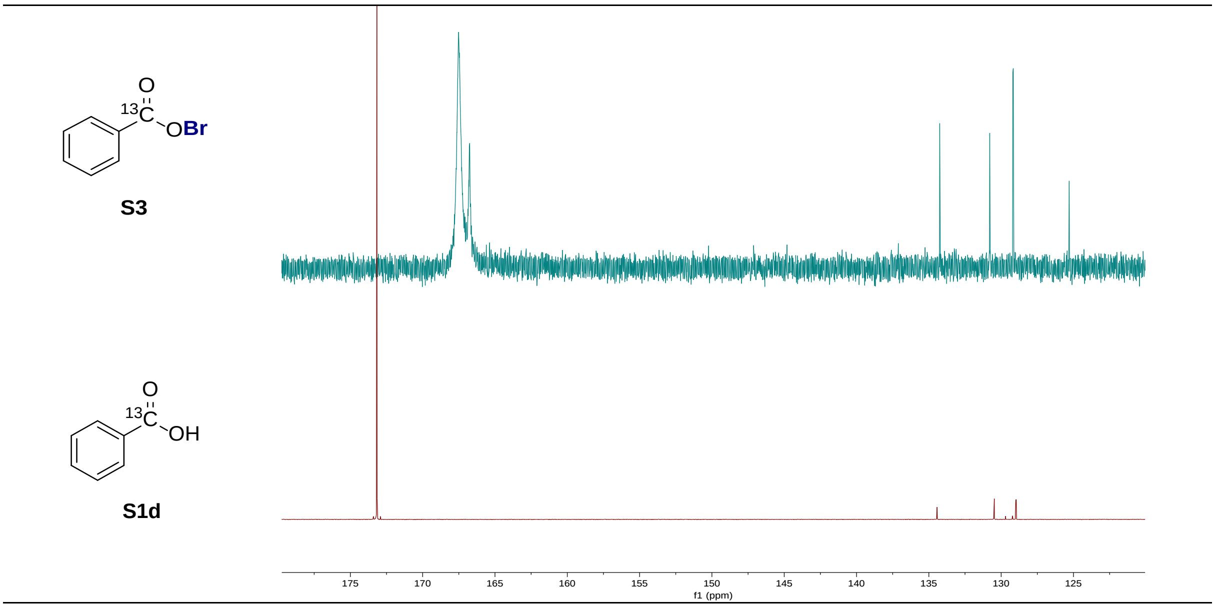

Figure S2. ^{13}C NMR of ^{13}C labeled benzoyl hypobromite **S3** and ^{13}C labeled benzoic acid **S1d** (151 MHz, CD_2Cl_2).

Preparation of aryl acyl hypobromite 1

Synthesis of ((2-bromobenzoyl)oxy)silver (**S2b**)

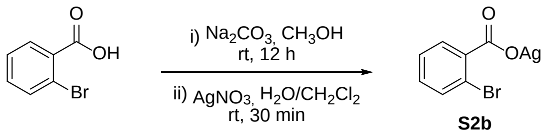

(3)

Silver benzoate **S2b** was synthesized in two-steps from the corresponding acid, which was first converted to the sodium salt: To a stirred suspension of Na_2CO_3 (265 mg, 2.5 mmol, 1.0 equiv) in methanol (5 mL) was added a solution of the acid (1.0 g, 5.0 mmol, 2.0 equiv) in methanol (10 mL) (eq. 3). The mixture was stirred for 12 hours at room temperature. After this time, the methanol was removed to quantitatively yield a white solid and the compound was dried under vacuum for 4 hours.

Following the modified procedure of Úbeda and co-workers,[9] to a magnetically stirred solution of the sodium benzoate (444 mg, 2.0 mmol, 1.0 equiv) in methylene chloride (8 mL) was added a solution of silver nitrate (340 mg, 2.0 mmol, 1.0 equiv, freshly recrystallized) in water (4 mL). A white precipitate formed and after 30 min the solid was collected by filtration and washed with methanol. The white solid was collected and dried at room temperature under vacuum overnight yielding silver carboxylate **S2b**.

IR (ATR): ν (cm^{-1}): 3186, 3057, 1621, 1596, 1490, 1312, 1171; **^{1}H NMR** (400 MHz, DMSO-d_6): δ (ppm): 7.54 (dd, *J* = 7.8, 1.2 Hz, 1H), 7.47 (dd, *J* = 7.6, 1.8 Hz, 1H), 7.31 (td, *J* = 7.6, 1.2 Hz, 1H), 7.19 (td, *J* = 7.8, 1.8 Hz, 1H); **^{13}C NMR** (100 MHz, DMSO-d_6): δ (ppm): 170. 9, 142.2, 132.5, 129.1, 129.0, 127.0, 118.8; **Elementa analysis:** found: 27.2% C, 1.2% H; calculated for 27.3% C, 1.3% H.

Synthesis of benzoyl hypobromite **1**

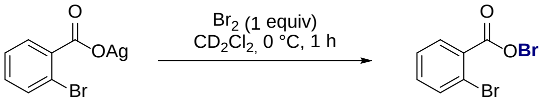

S2b **1** (4)

To a flame-dried Schlenk tube, foiled to protect from light, was placed silver benzoate **S2b** (153 mg, 0.5 mmol, 1.0 equiv) and d_2-methylene chloride (3 mL) (eq. 4). The suspension was cooled to 0 °C and bromine (36 µL, 0.7 mmol, 1.4 equiv). The suspension was allowed to stir for 1 hour at 0 °C and then filtered through a syringe filter. ^{1}H and ^{13}C NMR was measured immediately, at 0 °C.

NMR data for **1**

^{1}H NMR (600 MHz, CD_2Cl_2): δ (ppm): 7.75–7.72 (m, 1H), 7.71–7.67 (m, 1H), 7.44–7.39 (m, 2H); **^{13}C NMR** (151 MHz, CD_2Cl_2): δ (ppm): 166.4, 134.2, 133.3, 131.6, 128.7 (d, *J* = 7.8 Hz), 127.4, 121.5.

To determine whether the species prepared and characterized by NMR was the hypobromite, following measurement of ^{1}H and ^{13}C NMR spectra, cyclohexene (5.0 equiv) was added to the NMR tube. The solution immediately became colorless (consumption of the excess bromine), and after 5 min a second ^{1}H NMR was measured (Figure S3). This revealed that all the hypobromite had been consumed. It has been reported that acyl hypobromites can add across double bonds in a *trans* manner to yield 1,2-bromocarboxylates, such as **(±)-S5**.[11]

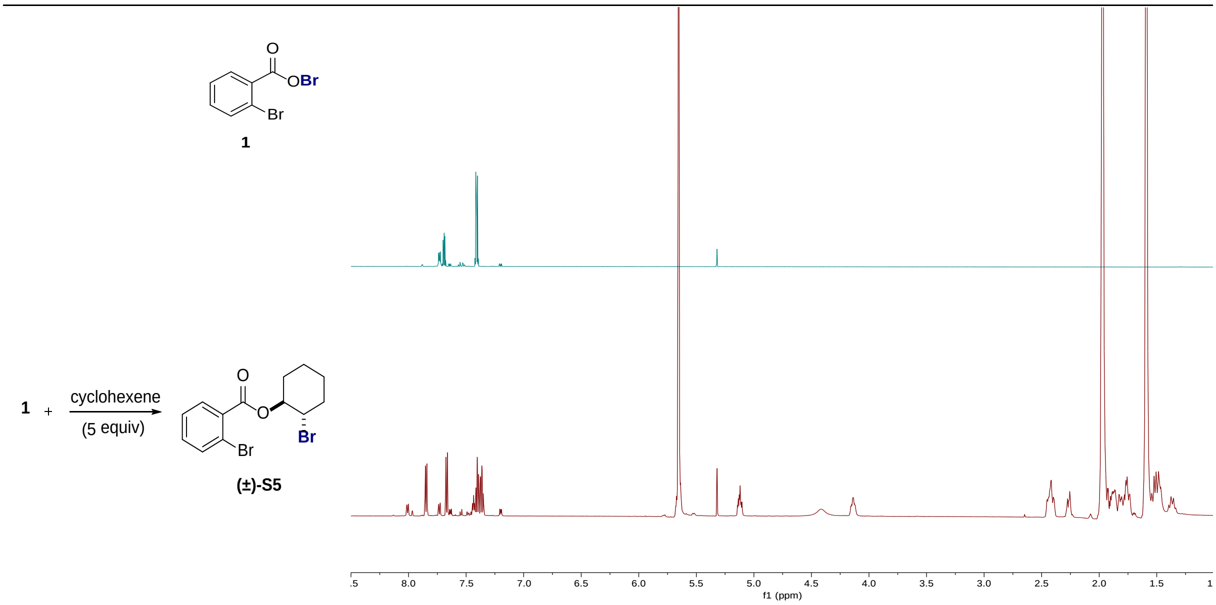

Figure S3. ^{1}H NMR of hypobromite **1** and 1,2-bromocarboxylates **(±)-S5** formed by the addition of cyclohexene (600 MHz, CD_2Cl_2).

Having determined that the benzoyl hypobromite could be prepared from the corresponding silver carboxylate and bromine, we next investigated whether it was a possible intermediate in the reaction. To this end, benzoyl hypobromite was synthesized by the addition of bromine (36 µL, 0.7 mmol, 1.0 equiv) to a suspension of silver benzoate **S6** (220 mg, 0.72 mmol, 1.2 equiv) in chlorobenzene (6 mL) at 0 °C. The suspension was allowed to stir for 1 hour at 0 °C and then filtered through a syringe filter and following filtration, subjected to a range of reaction conditions (Scheme S1). Hypobromite **1** (2 mL of a 0.1 M solution in chlorobenzene) was reacted in chlorobenzene (an additional 1 mL), in the presence of Cs_2CO_3 (2 equiv) at 55 °C in the absence of light and at this temperature under irradiation (blue LED, λ_{max} = 455 nm). Following analysis by GC-MS (decane as the internal standard) it was found that both sets of conditions yielded benzoate **2a** in 43% yield as a mixture of C2/C3/C4 isomers. This suggests that the thermolysis of the hypobromite at 55 °C provides the benzoyloxy radical, which undergoes addition to the chlorobenzene.

Next, hypobromite **1** (2 mL of a 0.1 M solution in chlorobenzene) was also subjected to irradiation by visible light in the presence of **PC** (6 mol%). Following irradiation (blue LED, λ_{max} = 455 nm) at 55 °C in the presence of Cs_2CO_3 (and an additional 1 mL of chlorobenzene) the reaction was analyzed by GC-MS (decane as the internal standard) and biaryl **3a** was observed (34% as a mixture of C2/C3/C4 isomers) along with **2** (36%), which presumably forms via background thermolysis under the conditions.

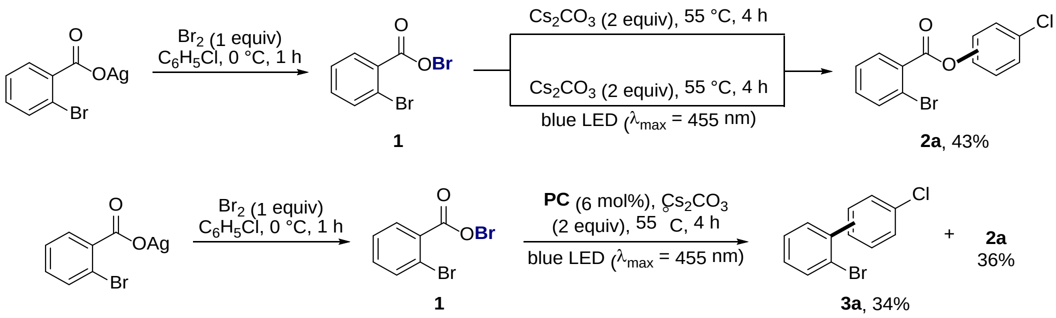

Scheme S1. Independent synthesis of hypobromite **1** and subjection to reaction conditions.

4. Optimization and control reactions

Chart S1. Screen of halogenation reagents

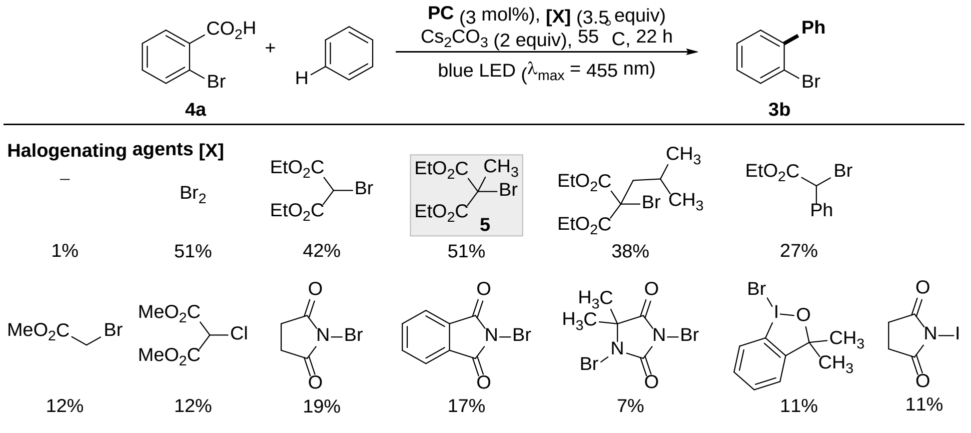

4a (0.1 mmol), halogenating agent **[X]** (3.5 equiv), Cs_2CO_3 (equiv), benzene (2.2 mL), 22 sh, blue LED (λ_{max} = 455 nm). [a] Yield determined by GC-MS using decane as standard.

Table S1. Optimization of visible light-promoted decarboxylation

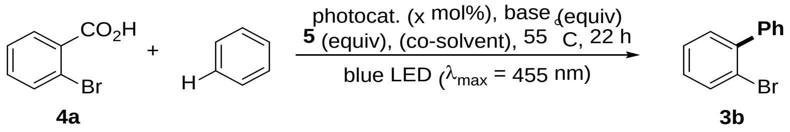

entry	conditions	yield (%)[a]
1	(Mes-Acr)ClO_4 (5 mol%), Cs_2CO_3 (2 equiv), **5** (3.5 equiv)	0
2	$[Ru(bpy)_3](PF_6)_2$ (2 mol%), Cs_2CO_3 (2 equiv), **5** (3.5 equiv)	0
3	$[Ir(ppy)_2(dtbbpy)]PF_6$ (2 mol%), Cs_2CO_3 (2 equiv), **5** (3.5 equiv)	0
4	$[Ru(bpz)_3](PF_6)_2$ (2 mol%), Cs_2CO_3 (2 equiv), **5** (3.5 equiv)	0
5	$[Ir(dF(CF_3)ppy)_2(dtbbpy)]PF_6$ (2 mol%), **5** (3.5 equiv), Cs_2CO_3 (2 equiv)	58
6	$[Ir(dF(CF_3)ppy)_2(dtbbpy)]PF_6$ (2 mol%), **5** (3.5 equiv), Cs_2CO_3 (1 equiv)	46
7	$[Ir(dF(CF_3)ppy)_2(dtbbpy)]PF_6$ (2 mol%), **5** (2.5 equiv), K_2CO_3 (1 equiv)	4
8	$[Ir(dF(CF_3)ppy)_2(dtbbpy)]PF_6$ (2 mol%), **5** (2.5 equiv), CsOH•H_2O (1 equiv)	0
9	$[Ir(dF(CF_3)ppy)_2(dtbbpy)]PF_6$ (2 mol%), **5** (2.5 equiv), CsOAc (1 equiv)	21
10	$[Ir(dF(CF_3)ppy)_2(dtbbpy)]PF_6$ (2 mol%), **5** (2.5 equiv), CsOBz (1 equiv)	26
11	$[Ir(dF(CF_3)ppy)_2(dtbbpy)]PF_6$ (2 mol%), **5** (2.5 equiv), DIPEA (0.5 equiv)	0
12	$[Ir(dF(CF_3)ppy)_2(dtbbpy)]PF_6$ (3 mol%), **5** (3.5 equiv), Cs_2CO_3 (2 equiv)	77
13	$[Ir(dF(CF_3)ppy)_2(dtbbpy)]PF_6$ (3 mol%), **5** (2.5 equiv), Cs_2CO_3 (1 equiv), 30 °C	19
14	$[Ir(dF(CF_3)ppy)_2(dtbbpy)]PF_6$ (3 mol%), **4** (2.5 equiv), Cs_2CO_3 (1 equiv), 30 °C, 120 h	75
15	entry 12, C_6H_6 (150 equiv, 1.3 mL), DCE (1.3 mL)	22[b]

16	entry 12, C_6H_6 (150 equiv, 1.3 mL), CH_2Cl_2 (1.3 mL)	17[b]
17	entry 12, C_6H_6 (150 equiv, 1.3 mL), CH_3CN (1.3 mL)	83 (81)
18	entry 12, C_6H_6 (150 equiv, 1.3 mL), DMSO (1.3 mL)	0
19	entry 12, C_6H_6 (150 equiv, 1.3 mL), EtOAc (1.3 mL)	25[b]
20	entry 12, blue LED (λ_{max} = 415 nm)	76
21	entry 12, no $[Ir(dF(CF_3)ppy)_2(dtbbpy)]PF_6$	0
22	entry 12, no light	0

4a (0.1 mmol), **5** (equiv), base (equiv), benzene (2.2 mL), co-solvent, 22 h, blue LED (λ_{max} = 455 nm). [a] Yield determined by GC-MS using decane as standard. [b] Bromobenzene observed as the major byproduct. Isolated yield in parentheses.

5. Oxidant Screen

Table S2. Screen of various oxidants

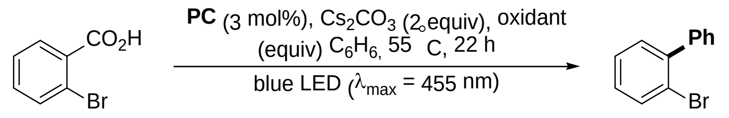

4a **3b**

entry	oxidant	yield (%)[a]
1	$Co(acac)_3$ (1.2 and 3.5 equiv)	5
2	O_2 (reaction not degassed)	5
3	O_2 (bubbled through reaction for 5 min)	trace
3	PhSH (1.2 and 3.5 equiv)	0
4	$K_2S_2O_8$ (1.2 and 3.5 equiv)	1
5	$(NH_4)_2S_2O_8$ (1.2 and 3.5 equiv)	1
6	viologen (1.2 equiv)	0

4a (0.1 mmol), oxidant (equiv), Cs_2CO_3 (2 equiv), C_6H_6 (2.2 mL.), 22 hr, blue LED (λ_{max} = 455 nm). [a] Yield determined by GC-FID using decane as standard.

6. General procedure for the visible light-promoted aryl decarboxylation

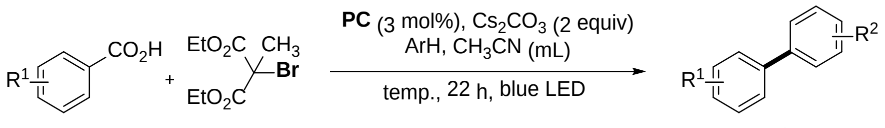

4 **5** **3**

General procedure A

To a 8 mL screw-capped vial with septum (not dried) Cs_2CO_3 (0.4 mmol, 2.0 equiv), $[Ir(dF(CF_3)ppy)_2(dtbbpy)](PF_6)$ (**PC**) (3 mol%), carboxylic acid (0.2 mmol, 1.0 equiv), arene (250 equiv) and diethyl 2-bromo-2-methylmalonate (**5**) (0.7 mmol, 3.5 equiv) were added. A needle was then inserted into the septum and the solution was purged with argon for 10 minutes. After this time, the needle was removed and the vial was sealed with Parafilm M®. The mixture was stirred under irradiation

from blue LEDs (λ_{max} = 455 nm) (situated ~ 3 cm away from the reaction vessel in a custom-made "light box", see Figure S1). Using a contact thermometer, the temperature of the solvent in the reaction was measured to be 55 °C during the reaction, after equilibration. After 22 hours, the reaction was filtered through a 1 cm thick pad of silica and the silica was washed with methylene chloride (50 mL). The filtrate was collected and the solvent removed *in vacuo*. The crude residue was purified by silica gel flash column chromatography to provide the pure product.

General procedure B

To a 8 mL screw-capped vial with septum (not dried) Cs_2CO_3 (0.4 mmol, 2.0 equiv), **PC** (3 mol%), carboxylic acid (0.2 mmol, 1.0 equiv), arene (150 equiv), acetonitrile (2.6 ml) and **5** (0.7 mmol, 3.5 equiv) were added. A needle was then inserted into the septum and the solution was purged with argon for 10 minutes. After this time, the needle was removed and the vial was sealed with Parafilm M®. The mixture was stirred at 55 °C under irradiation from blue LEDs (λ_{max} = 455 nm) in a light box. After 22 hours, the reaction was filtered through a 1 cm thick pad of silica and the silica was washed with methylene chloride (50 mL). The filtrate was collected and the solvent removed *in vacuo*. The crude residue was purified by silica gel flash column chromatography to provide the pure product.

General procedure C

To a 20 mL Schlenk tube with quick fit (not dried) and septum Cs_2CO_3 (0.4 mmol, 2 equiv), **PC** (3 mol%), carboxylic acid (0.2 mmol, 1.0 equiv), arene (250 equiv) and **5** (3.5 equiv) were added. A needle was then inserted into the septum and the solution was purged with argon for 10 minutes. After this time, the needle and spetum were removed and a glass rod with quick fit was inserted into the Schlenk so that the glass rod sat 1 cm above the reaction mixture. At the other end of the glass rod was placed the blue LED (λ_{max} = 415 nm), see Figure S1. The reaction mixture was heated to 80 °C for 22 hours and after this time the reaction was filtered through a 1 cm thick pad of silica and the silica was washed with methylene chloride (50 mL). The filtrate was collected and the solvent removed *in vacuo*. The crude residue was purified by silica gel flash column chromatography to provide the pure product.

7. Scope of the visible light-promoted decarboxylation/radical trapping

2-Bromo-1,1'-biphenyl (3b)[12]

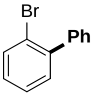

The title compound was prepared from 2-bromobenzoic acid and benzene following general procedure B (38.1 mg, 81%) as a white solid. Yield is the average from two reactions. All data was consistent with that previously reported.[12]

IR (ATR): ν (cm^{-1}): 3055, 1466, 1420, 1026, 1007, 745, 689; **R*f*** = 0.6 (pentane); **^{1}H NMR** (300 MHz, $CDCl_3$): δ (ppm): 7.69 (dd, *J* = 8.0, 1.2 Hz, 1H), 7.48–7.33 (m, 7H), 7.22 (ddd, *J* = 8.0, 6.7, 2.4 Hz, 1H); **^{13}C NMR** (75 MHz, $CDCl_3$): δ (ppm): 142.7, 141.2, 133.2, 131.4, 129.5, 128.8, 128.1, 127.7, 127.5, 122.8.

2-Fluoro-1,1'-biphenyl (3c)[13]

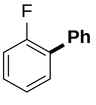

The title compound was prepared from 2-fluorobenzoic acid and benzene following general procedure A (19.5 mg, 56%) as a colorless oil. Yield is the average from two reactions. All data was consistent with that previously reported.[13]

IR (ATR): ν (cm^{-1}): 3036, 1481, 1435, 1250, 1215, 822, 752, 694; **R*f*** = 0.6 (pentane); **^{1}H NMR** (300 MHz, $CDCl_3$): δ (ppm): 7.62–7.54 (m, 2H), 7.52–7.42 (m, 3H), 7.42 –7.29 (m, 2H), 7.27–7.14 (m, 2H); **^{13}C NMR** (75 MHz, $CDCl_3$): δ (ppm): 159.9 (d, *J* = 247.6 Hz), 136.0, 130.9 (d, *J* = 3.5 Hz), 129.1(7) (d, *J* = 3.1 Hz), 129.1(5) (d, *J* = 18.9 Hz), 129.0 (d, *J* = 9.0 Hz), 128.6, 127.8, 124.5 (d, *J* = 3.7 Hz), 116.2 (d, *J* = 22.8 Hz); **^{19}F NMR** (282MHz, $CDCl_3$): δ (ppm): -118.1.

2-Chloro-1,1'-biphenyl (3d)[14]

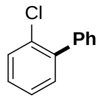

The title compound was prepared from 2-chlorobenzoic acid and benzene following general procedure B (28.3 mg, 74%) as a colorless oil. Yield is the average from two reactions. All data was consistent with that previously reported.[14]

IR (ATR): ν (cm^{-1}): 3059, 1466, 1424, 1126, 1076, 1038, 745, 698; $\mathbf{R}_f$ = 0.7 (pentane); **^{1}H NMR** (300 MHz, $CDCl_3$): δ (ppm): 7.50–7.37 (m, 6H), 7.37–7.27 (m, 3H); **^{13}C NMR** (75 MHz, $CDCl_3$): δ (ppm): 140.7, 139.6, 132.7, 131.5, 130.1, 129.6, 128.7, 128.2, 127.7, 127.0.

2-Iodo-1,1'-biphenyl (3e)[15]

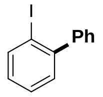

The title compound was prepared from 2-iodobenzoic acid and benzene following general procedure B (28.7 mg, 51%) as a white solid. Yield is the average from two reactions. All data was consistent with that previously reported.[15]

IR (ATR): ν (cm^{-1}): 3055, 1458, 1427, 1015, 1003, 745, 689; $\mathbf{R}_f$ = 0.6 (pentane); **^{1}H NMR** (300 MHz, $CDCl_3$): δ (ppm): 7.96 (dd, *J* = 7.9, 1.1 Hz, 1H), 7.48–7.28 (m, 7H), 7.04 (td, *J* = 7.9, 1.9 Hz, 1H); **^{13}C NMR** (75 MHz, $CDCl_3$): δ (ppm): 146.9, 144.4, 139.7, 130.3, 129.5, 129.0, 128.3, 128.2, 127.9, 98.9.

2,4-Dichloro-1,1'-biphenyl (3f)[16]

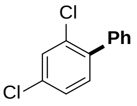

The title compound was prepared from 2,4-dichlorobenzoic acid and benzene following general procedure A (37.3 mg, 84%) as a white solid. Yield is the average from two reactions. All data was consistent with that previously reported.[16]

IR (ATR): ν (cm^{-1}): 1578, 1462, 1373, 1103, 1072, 1007, 810, 760, 698; $\mathbf{R}_f$ = 0.5 (pentane); **^{1}H NMR** (300 MHz, $CDCl_3$): δ (ppm): 7.50 (d, *J* = 1.9 Hz, 1H), 7.49–7.36 (m, 5H), 7.31–7.28 (m, 2H);**^{13}C NMR** (75 MHz, $CDCl_3$): δ (ppm): 139.2, 138.4, 133.8, 133.4, 132.2, 129.8, 129.5, 128.3, 128.0, 127.3.

3-Bromo-1,1'-biphenyl (3g)[17]

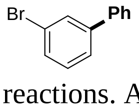

The title compound was prepared from 3-bromobenzoic acid and benzene following general procedure C (38.1 mg, 82%) as a white solid. Yield is the average from two reactions. All data was consistent with that previously reported.[17]

IR (ATR): ν (cm^{-1}): 1589, 1559, 1470, 1042, 880, 748, 694; $\mathbf{R}_f$ = 0.6 (pentane); **^{1}H NMR** (300 MHz, $CDCl_3$): δ (ppm): 7.76 (t, *J* = 1.9 Hz, 1H), 7.61–7.43 (m, 6H), 7.42–7.36 (m, 1H), 7.32 (t, *J* = 7.8 Hz, 1H); **^{13}C NMR** (75 MHz, $CDCl_3$): δ (ppm): 143.5, 139.8, 130.4, 130.3, 130.3, 129.0, 128.0, 127.2, 125.9, 123.0.

4-Bromo-1,1'-biphenyl (3h)[18]

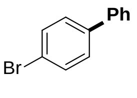

The title compound was prepared from 4-bromobenzoic acid and benzene following general procedure C (30.5 mg, 65%) as a white solid. Yield is the average from two reactions. All data was consistent with that previously reported.[18]

IR (ATR): ν (cm^{-1}): 3063, 1586, 1474, 1393, 1076, 1003, 829, 752, 687; $\mathbf{R}_f$ = 0.6 (pentane); **^{1}H NMR** (300 MHz, $CDCl_3$): δ (ppm): 7.60–7.53 (m, 4H), 7.49–7.41 (m, 4H), 7.40–7.34 (m, 1H); **^{13}C NMR** (75 MHz, $CDCl_3$): δ (ppm): 140.3, 140.1, 132.0, 129.0, 128.9, 127.8, 127.1, 121.7.

1,1'-Biphenyl (3i)[19]

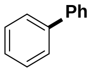

The title compound was prepared from benzoic acid and benzene following general procedure C (16.0 mg, 52%) as a white solid. Yield is the average from two reactions. All data was consistent with that previously reported.[19]

IR (ATR): ν (cm^{-1}): 3048, 1597, 1481, 1431, 1265, 1007, 729, 694; $\mathbf{R}_f$ = 0.6 (pentane); **^{1}H NMR** (300 MHz, $CDCl_3$): δ (ppm): 7.68–7.61 (m, 4H), 7.53–7.45 (m, 4H), 7.42–7.34 (m, 2H); **^{13}C NMR** (75 MHz, $CDCl_3$): δ (ppm): 141.3, 128.8, 127.3, 127.2.

1,1':4',1''-Terphenyl (3j)[19]

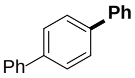

The title compound was prepared from 4-phenylbenzoic acid and benzene following general procedure A (31.4 mg, 68%) as a white solid. Yield is the average from two reactions. All data was consistent with that previously reported.[19]

IR (ATR): ν (cm^{-1}): 1481, 1454, 1404, 837, 745, 687; $\mathbf{R}_f$ = 0.7 (pentane); **^{1}H NMR** (300 MHz, $CDCl_3$): δ (ppm): 7.70–7.63 (m, 8H), 7.52–7.44 (m, 4H), 7.41–7.33 (m, 2H); **^{13}C NMR** (75 MHz, $CDCl_3$): δ (ppm): 140.8, 140.3, 129.0, 127.6, 127.5, 127.2.

4-(*tert*-Butyl)-1,1'-biphenyl (3k)[20]

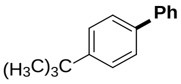

The title compound was prepared from 4-*tert*-butylbenzoic acid and benzene following general procedure A however, 2.0 equiv of **5** was used, (23.9 mg, 57%) as a colorless oil. Yield is the average from two reactions. All data was consistent with that previously reported.[20]

IR (ATR): ν (cm^{-1}):2963, 1485, 1362, 1269, 1115, 837, 764, 733, 694; $\mathbf{R}_f$ = 0.7 (pentane); **^{1}H NMR** (300 MHz, $CDCl_3$): δ (ppm): 7.65–7.61 (m, 2H), 7.58 (AA′BB′, *J* = 9.2 Hz, 2H), 7.50 (AA′BB′, *J* = 9.2 Hz, 2H), 7.48–7.42 (m, 2H), 7.39–7.31 (m, 1H), 1.40 (s, 9H); **^{13}C NMR** (75 MHz, $CDCl_3$): δ (ppm): 150.4, 141.2, 138.5, 128.8, 127.2, 127.1, 126.9, 125.9, 34.7, 31.5.

4-Methoxy-1,1'-biphenyl (3l)[21]

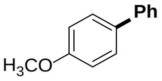

The title compound was prepared from 4-methoxybenzoic acid and benzene following general procedure A however, 2.0 equiv of **5** was used, (22.4 mg, 61%) as a white solid. Yield is the average from two reactions. All data was consistent with that previously reported.[21]

IR (ATR): ν (cm^{-1}): 3001, 2835, 1605, 1520, 1481, 1439, 1269, 1246, 1200, 1184, 1034, 833, 760, 698; $\mathbf{R}_f$ = 0.3 (pentane:EtOAc, 19:1); **^{1}H NMR** (300 MHz, $CDCl_3$): δ (ppm): 7.59–7.56 (m, 2H), 7.55 (d, *J* = 9.2 Hz, 2H), 7.47–7.40 (m, 2H), 7.37–7.28 (m, 1H), 7.00 (d, *J* = 9.2 Hz, 2H), 3.86 (s, 3H); **^{13}C NMR** (75 MHz, $CDCl_3$): δ (ppm): 159.3, 141.0, 133.9, 128.9, 128.3, 126.9, 126.8, 114.3, 55.5.

([1,1'-Biphenyl]-4-yloxy)(tert-butyl)dimethylsilane (3m)[22]

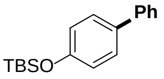

The title compound was prepared from 4-((*tert*-butyldimethylsilyl)oxy)benzoic acid and benzene following general procedure A however, 2.0 equiv of **5** was used, (30.4 mg, 54%) as a colorless oil. Yield is the average from two reactions. All data was consistent with that previously reported.[22]

IR (ATR): ν (cm^{-1}): 2928, 2859, 1520, 1489, 1269, 1254, 922, 845, 764, 691; $\mathbf{R}_f$ = 0.6 (pentane:EtOAc, 25:1); ^{1}**H NMR** (400 MHz, $CDCl_3$): δ (ppm): 7.63–7.54 (m, 2H), 7.48 (AA′BB′, *J* = 8.0 Hz, 2H), 7.42 (ddd, *J* = 8.2, 6.6, 1.4, Hz, 2H), 7.35–7.28 (m, 1H), 6.92 (AA′BB′, *J* = 8.0 Hz, 2H), 1.02 (s, 9H), 0.25 (s, 6H); ^{13}**C NMR** (101 MHz, $CDCl_3$): δ (ppm): 155.4, 141.1, 134.4, 128.8, 128.2, 126.9, 126.8, 120.5, 25.9, 18.4, -4.2.

3-Methoxy-1,1'-biphenyl (3n)[23]

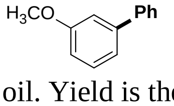

The title compound was prepared from 3-methoxybenzoic acid and benzene following general procedure A however, 2.0 equiv of **5** was used, (22.8 mg, 62%) as a colorless oil. Yield is the average from two reactions. All data was consistent with that previously reported.[23]

IR (ATR): ν (cm^{-1}): 2835, 1597, 1574, 1477, 1420, 1296, 1211, 1038, 756, 694; $\mathbf{R}_f$ = 0.4 (pentane:EtOAc, 19:1); ^{1}**H NMR** (300 MHz, $CDCl_3$): δ (ppm): 7.65–7.59 (m, 2H), 7.46 (tt, *J* = 6.6, 0.9 Hz, 2H), 7.42–7.34 (m, 2H), 7.21 (ddd, *J* = 8.2, 1.6, 0.9 Hz, 1H), 7.16 (dd, *J* = 2.6, 1.6 Hz, 1H), 6.92 (ddd, *J* = 8.2, 2.6, 0.9 Hz, 1H), 3.89 (s, 3H); ^{13}**C NMR** (75 MHz, $CDCl_3$): δ (ppm): 160.1, 142.9, 141.2, 129.9, 128.9, 127.5, 127.3, 119.8, 113.0, 112.8, 55.4.

3-Methoxy-1,1'-biphenyl was contaminated with 13% 1-bromo-3-methoxybenzene:[24]

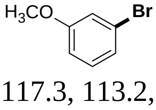

^{1}**H NMR** (300 MHz, $CDCl_3$): δ (ppm): 7.20–7.07 (m, 3H), 6.86 (ddd, *J* = 8.0, 2.4, 1.4 Hz, 1H), 3.81 (s, 3H); ^{13}**C NMR** (75 MHz, $CDCl_3$): δ (ppm): 132.8, 130.7, 123.9, 123.0, 117.3, 113.2, 55.6.

[1,1'-Biphenyl]-4-carbonitrile (3o)[17]

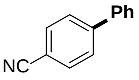

The title compound was prepared from 4-cyanobenzoic acid and benzene following general procedure C (16.9 mg, 47%) as a colorless oil. Yield is the average from two reactions. All data was consistent with that previously reported.[17]

IR (ATR): ν (cm^{-1}): 2226, 1605, 1481, 1397, 845, 768, 698; $\mathbf{R}_f$ = 0.3 (pentane:EtOAc, 30:1); ^{1}**H NMR** (400 MHz, $CDCl_3$): δ (ppm): 7.73 (AA′BB′, *J* = 8.0 Hz, 2H), 7.69 (AA′BB′, *J* = 8.0 Hz, 2H), 7.62–7.57 (m, 2H), 7.52–7.46 (m, 2H), 7.45–7.40 (m, 1H); ^{13}**C NMR** (101 MHz, $CDCl_3$): δ (ppm): 145.8, 139.3, 132.7, 129.2, 128.8, 127.9, 127.4, 119.1, 111.1

2-Bromo-5-(trifluoromethyl)-1,1'-biphenyl (3p)

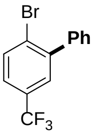

The title compound was prepared from 2-bromo-5-(trifluoromethyl)benzoic acid and benzene following general procedure A (43.2 mg, 72%) as a colorless oil. Yield is the average from two reactions.

IR (ATR): ν (cm^{-1}): 1404, 1331, 1285, 1169, 1123, 1084, 1015, 826, 764, 698; $\mathbf{R}_f$ = 0.7 (pentane); ^{1}**H NMR** (300 MHz, $CDCl_3$): δ (ppm): 7.81 (dd, *J* = 8.3, 0.9 Hz, 1H), 7.59 (d, *J* = 2.3 Hz, 1H), 7.52–7.38 (m, 6H); ^{13}**C NMR** (75 MHz, $CDCl_3$): δ (ppm): 143.5, 139.9, 133.9, 130.2 (q, *J* = 32.9 Hz) 129.4, 128.7 (q, *J* = 255 Hz), 128.3(9), 128.3(8), 128.1 (q, *J* = 3.7 Hz), 125.4 (q, *J* = 3.7 Hz); ^{19}**F NMR** (282MHz, $CDCl_3$): δ (ppm): -62.6; **HRMS** (APCI): m/z calculated for $[C_{13}H_8Br^{79}F_3^{+}]$ $[M^{+}]$: 299.9761, measured 299.9756.

1-([1,1'-Biphenyl]-2-yl)ethan-1-one (3q)[25]

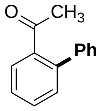

The title compound was prepared from 2-acetylbenzoic acid and benzene following general procedure A (28.6 mg, 73%) as a colorless oil. Yield is the average from two reactions. All data was consistent with that previously reported.[25]

IR (ATR): ν (cm^{-1}): 1686, 1593, 1474, 1354, 1269, 1234, 760, 741, 702; **R_f** = 0.4 (pentane:EtOAc, 9:1); **^{1}H NMR** (300 MHz, $CDCl_3$): δ (ppm): 7.60–7.47 (m, 2H), 7.47–7.32 (m, 7H), 2.01 (s, 3H); **^{13}C NMR** (75 MHz, $CDCl_3$): δ (ppm): 205.1, 141.0, 140.9, 140.6, 130.9, 130.4, 129.0, 128.8, 128.0, 128.0, 127.6, 30.6.

2-Chloro-3-phenylpyridine (3r)[17]

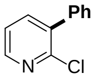

The title compound was prepared from 2-chloronicotinic acid and benzene following general procedure A (27.6 mg, 73%) as a white solid. Yield is the average from two reactions. All data was consistent with that previously reported.[17]

IR (ATR): ν (cm^{-1}): 3055, 1559, 1439, 1389, 1099, 760, 689; **R_f** = 0.3 (pentane:EtOAc, 30:1); **^{1}H NMR** (300 MHz, $CDCl_3$): δ (ppm): 8.39 (dd, *J* = 4.8, 1.9 Hz, 1H), 7.67 (dd, *J* = 7.6, 1.9 Hz, 1H), 7.50–7.42 (m, 5H), 7.31 (dd, *J* = 7.6, 4.8 Hz, 1H); **^{13}C NMR** (75 MHz, $CDCl_3$): δ (ppm): 149.8, 148.5, 139.8, 137.5, 137.1, 129.4, 128.4(2), 128.4(0), 122.6.

2-Bromo-3-phenylpyridine (3s)[26]

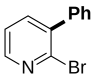

The title compound was prepared from 2-bromonicotinic acid and benzene following general procedure A (36.3 mg, 78%) as a white solid. Yield is the average from two reactions. All data was consistent with that previously reported.[26]

IR (ATR): ν (cm^{-1}): 3051, 1551, 1439, 1385, 1092, 1053, 760, 698; **R_f** = 0.3 (pentane:EtOAc, 30:1); **^{1}H NMR** (300 MHz, $CDCl_3$): δ (ppm): 8.38 (dd, *J* = 4.7, 2.0 Hz, 1H), 7.63 (dd, *J* = 7.6, 2.0 Hz, 1H), 7.56–7.39 (m, 5H), 7.34 (dd, *J* = 7.6, 4.7 Hz, 1H); **^{13}C NMR** (75 MHz, $CDCl_3$): δ (ppm): 148.9, 142.5, 139.9, 139.2, 139.0, 129.4, 128.5, 128.4, 122.9.

2-Methoxy-3-phenylpyridine (3t)[17]

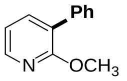

The title compound was prepared from 2-methoxynicotinic acid and benzene following general procedure A (21.5 mg, 58%) as a white solid. Yield is the average from two reactions. All data was consistent with that previously reported.[17]

IR (ATR): ν (cm^{-1}): 1586, 1447, 1400, 1250, 1019, 907, 729, 698; **R_f** = 0.4 (pentane:EtOAc, 19:1); **^{1}H NMR** (300 MHz, $CDCl_3$): δ (ppm): 8.17 (dd, *J* = 5.0, 1.9 Hz, 1H), 7.62 (dd, *J* = 7.3, 1.9 Hz, 1H), 7.59–7.53 (m, 2H), 7.43 (tt, *J* = 6.4, 1.1 Hz, 2H), 7.40–7.32 (m, 1H), 6.98 (dd, *J* = 7.3, 5.0 Hz, 1H), 3.98 (s, 3H); **^{13}C NMR** (75 MHz, $CDCl_3$): δ (ppm): 161.0, 145.8, 138.7, 136.9, 129.3, 128.4, 127.7, 124.8, 117.2, 53.7.

3-Chloro-4-phenylpyridine (3u)[17]

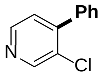

The title compound was prepared from 3-chloroisonicotinic acid and benzene following general procedure A (25.3 mg, 67%) as a white solid. Yield is the average from two reactions. All data was consistent with that previously reported.[17]

IR (ATR): ν (cm^{-1}): 1578, 1466, 1397, 1103, 1034, 837, 768, 694; **R$_f$** = 0.4 (pentane:EtOAc, 30:1); **^{1}H NMR** (400 MHz, $CDCl_3$): δ (ppm): 8.68 (d, *J* = 0.6 Hz, 1H), 8.52 (d, *J* = 5.0 Hz, 1H), 7.52–7.45 (m, 5H), 7.28 (dd, *J* = 5.0, 0.6 Hz, 1H); **^{13}C NMR** (101 MHz, $CDCl_3$): δ (ppm): 150.3, 148.0, 147.7, 136.7, 130.3, 129.1, 129.0, 128.6, 125.5.

4'-(*tert*-Butyl)-2,5-difluoro-1,1'-biphenyl (3v)

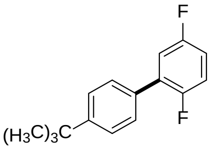

The title compound was prepared from 4-*tert*-butylbenzoic acid and 1,4-difluorobenzene following general procedure A however, 2.0 equiv of **5** was used, (27.6 mg, 56%) as a white solid. Yield is the average from two reactions.

IR (ATR): ν (cm^{-1}): 2966, 1458, 1099, 1031, 833, 812; **R$_f$** = 0.3 (pentane); **^{1}H NMR** (300 MHz, $CDCl_3$): δ (ppm): 7.50 (s, 4H), 7.20–7.05 (m, 2H), 6.98 (dddd, *J* = 9.0, 7.5, 3.7, 3.1 Hz, 1H), 1.38 (s, 9H); **^{13}C NMR** (75 MHz, $CDCl_3$): δ (ppm): 159.0 (dd, *J* = 224.8, 2.4 Hz), 155.8 (dd, *J* = 224.8, 2.4 Hz), 151.4, 132.0, 130.4 (dd, *J* = 15.8, 8.1 Hz), 128.7 (d, *J* = 3.2 Hz), 125.7, 117.4 (d, *J* = 8.8 Hz), 117.1, 117.05–114.64 (m), 34.8, 31.4; **^{19}F NMR** (282MHz, $CDCl_3$): δ (ppm): -119.25 (d, *J* = 17.7 Hz), -124.15 (d, *J* = 17.7 Hz); **HRMS** (APCI): m/z calculated for [$C_{15}H_{13}F_2^-$] [M-CH_3]: 231.0991, measured 231.0988.

4'-(*tert*-Butyl)-2,5-dichloro-1,1'-biphenyl (3w)

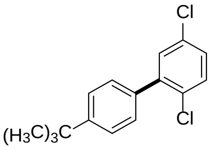

The title compound was prepared from 4-*tert*-butylbenzoic acid and 1,4-dichlorobenzene following general procedure A however, 2.0 equiv of **5** was used, (34.0 mg, 61%) as a white solid. Yield is the average from two reactions.

IR (ATR): ν (cm^{-1}): 2963, 1458, 1099, 1030, 833, 810; **R$_f$** = 0.3 (pentane); **^{1}H NMR** (300 MHz, $CDCl_3$): δ (ppm): 7.46 (AA′BB′, *J* = 9.2 Hz, 2H), 7.42–7.32 (m, 4H), 7.24 (dd, *J* = 8.6, 2.6 Hz, 1H), 1.37 (s, 9H); **^{13}C NMR** (75 MHz, $CDCl_3$): δ (ppm): 151.2, 142.0, 135.4, 132.6, 131.4, 131.2, 131.0, 129.1, 128.3, 125.3, 34.8, 31.5; **HRMS** (APCI): m/z calculated for [$C_{15}H_{13}Cl_2^-$] [M-CH_3]: 263.0388, measured 263.0400

3x as a mixture of C2/C3/C4 isomers: **2-Bromo-2'-(*tert*-butyl)-1,1'-biphenyl**,[27] **2-bromo-3'-(*tert*-butyl)-1,1'-biphenyl**, **2-bromo-4'-(*tert*-butyl)-1,1'-biphenyl**[28]

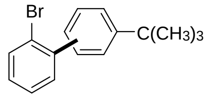

The title compound was prepared from 2-bromobenzoic acid and *tert*-butylbenzene following general procedure A (45.5 mg, 79%) as a mixture of C2/C3/C4 isomers (21:50:29) as a white solid. Yield is the average from two reactions. All data was consistent with that previously reported.

IR (ATR): ν (cm^{-1}): 2963, 1466, 1362, 1026, 1003, 752, 706; **R$_f$** = 0.4 (pentane); **^{1}H NMR** (300 MHz, $CDCl_3$): δ (ppm): 7.72–7.65 (m, 0.9H), 7.64–7.61 (m, 0.3H), 7.57 (dd, *J* = 8.2, 1.3 Hz, 0.3H), 7.50–7.30 (m, 4.6H), 7.25-7.18 (tdd, *J* = 9.7, 3.9, 1.8 Hz, 1.6H), 6.94 (dd, *J* = 7.6, 1.7 Hz, 0.3H), 1.38 (s, 2.8H), 1.38 (s, 4.5H), 1.21 (s, 1.7H) as a mixture of 3 compounds, 17H reported; **2-bromo-2'-(*tert*-butyl)-1,1'-biphenyl**[23] **^{13}C NMR** (75 MHz, $CDCl_3$): δ (ppm): 147.4, 145.7, 140.2, 132.5, 132.3, 132.0, 128.6, 127.9, 127.5, 126.3, 125.3, 125.1, 36.7, 32.4; **2-bromo-3'-(*tert*-butyl)-1,1'-biphenyl** **^{13}C NMR** (75 MHz, $CDCl_3$): δ (ppm): 150.8, 143.2, 140.8, 133.3, 131.5, 128.7, 127.8, 127.0, 126.5, 124.6, 122.8, 34.9, 31.5 (one signal overlapping); **2-bromo-4'-(*tert*-butyl)-1,1'-biphenyl**[24] **^{13}C NMR** (75 MHz, $CDCl_3$): δ (ppm): 150.6, 142.6, 138.2, 133.3, 131.6, 129.2, 128.6, 127.5, 125.0, 122.9, 34.8, 31.5.

3a as a mixture of C2/C3/C4 isomers: **2-Bromo-2'-chloro-1,1'-biphenyl**,[29] **2-bromo-3'-chloro-1,1'-biphenyl**,[30] **2-bromo-4'-chloro-1,1'-biphenyl**[28]

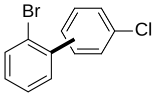

The title compound was prepared from 2-bromobenzoic acid and chlorobenzene following general procedure A (27.7 mg, 52%) as a mixture of C2/C3/C4 isomers (12:57:31) as an off-white oil. Yield is the average from two reactions. All data was consistent with that previously reported.

IR (ATR): ν (cm^{-1}): 3055, 1458, 1427, 1003, 748, 691; $\mathbf{R}_f$ = 0.3 (pentane); **^{1}H NMR** (300 MHz, $CDCl_3$): δ (ppm): 7.71–7.66 (m, 1H), 7.53–7.48 (m, 0.5H), 7.45–7.34 (m, 3.5H), 7.33–7.20 (m, 3H) as a mixture of 3 compounds, 8H reported; **2-bromo-2'-chloro-1,1'-biphenyl**[25] **^{13}C NMR** (75 MHz, $CDCl_3$): δ (ppm): 140.5, 140.1, 133.5, 132.7, 131.3, 131.2, 129.6, 129.5, 129.4, 127.3, 126.6, 123.8; **2-bromo-3'-chloro-1,1'-biphenyl**[26] **^{13}C NMR** (75 MHz, $CDCl_3$): δ (ppm): 142.9, 141.3, 134.0, 133.4, 131.2, 129.6, 129.4, 129.2, 127.9, 127.8, 127.6, 122.5; **2-bromo-4'-chloro-1,1'-biphenyl**[24] **^{13}C NMR** (75 MHz, $CDCl_3$): δ (ppm):141.5, 139.6, 133.9, 133.4, 131.2, 131.0, 129.2, 128.4, 127.6, 122.6.

3y as a mixture of C2/C3/C4 isomers: **2-Bromo-2'-(trifluoromethyl)-1,1'-biphenyl**, **2-bromo-3'-(trifluoromethyl)-1,1'-biphenyl**, **2-bromo-4'-(trifluoromethyl)-1,1'-biphenyl**[28]

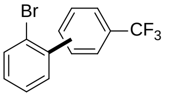

The title compound was prepared from 2-bromobenzoic acid and α,α,α-trifluorotoluene following general procedure A (24.6 mg, 41%) as a mixture of C2/C3/C4 isomers (5:60:35) as an off-white oil. Yield is the average from two reactions. All data was consistent with that previously reported. 2-Bromo-3'-(trifluoromethyl)-1,1'-biphenyl was synthesized independently to aid characterization.

IR (ATR): ν (cm^{-1}): 3059, 1335, 1323, 1165, 1126, 1076, 1018, 756; $\mathbf{R}_f$ = 0.4 (pentane); **2-bromo-3'-(trifluoromethyl)-1,1'-biphenyl** **^{1}H NMR** (300 MHz, $CDCl_3$): δ (ppm): 7.73–7.52 (m, 5H), 7.44–7.31 (m, 2H), 7.26 (ddd, J = 8.1, 7.1, 2.0 Hz, 1H); **^{13}C NMR** (75 MHz, $CDCl_3$): δ (ppm): 141.8, 141.2, 133.4, 133.0 (q, J = 1.4 Hz), 131.3, 130.6 (q, J = 32.3 Hz), 129.5, 128.6, 127.7, 126.4 (q, J = 3.8 Hz), 124.5 (q, J = 3.8 Hz), 124.2 (q, J = 270.2 Hz), 122.6; **^{19}F NMR** (282MHz, $CDCl_3$): δ (ppm): -62.5; **2-bromo-4'-(trifluoromethyl)-1,1'-biphenyl**[24] **^{13}C NMR** (75 MHz, $CDCl_3$): δ (ppm): 144.7 (q, J = 1.6 Hz), 141.3, 133.4, 131.2, 130.0, 129.9 (q, J = 32.7 Hz), 129.6, 127.8, 125.1 (q, J = 3.8 Hz), 124.4 (q, J = 272.2 Hz), 122.4

2-(4-(*tert*-Butyl)phenyl)pyridine (3z)[31]

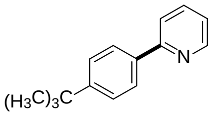

The title compound was prepared from 4-*tert*-butylbenzoic acid and pyridine following general procedure A however, 2.0 equiv of **5** was used, (28.3 mg, 67%) as a mixture of C2/C3+C4 isomers (62:38) as an colorless oil. Data reported is for major isomer 2-(4-(*tert*-butyl)phenyl)pyridine. Yield is the average from two reactions. All data was consistent with that previously reported.[31]

IR (ATR): ν (cm^{-1}): 2963, 2905, 1607, 1466, 1435, 1269, 1015, 845, 779, 733; $\mathbf{R}_f$ = 0.3 (pentane:EtOAc, 9:1); **^{1}H NMR** (300 MHz, $CDCl_3$): δ (ppm): 8.69 (dt, J = 4.8, 1.4 Hz, 1H), 7.94 (AA′BB′, J = 6.2 Hz, 2H), 7.74–7.70 (m, 2H), 7.51 (AA′BB′, J = 6.2 Hz, 2H), 7.20 (td, J = 4.8, 3.5 Hz, 1H), 1.37 (s, 9H); **^{13}C NMR** (75 MHz, $CDCl_3$): δ (ppm): 157.5, 152.2, 149.7, 136.7, 136.7, 126.7, 125.8, 121.9, 120.4, 34.8, 31.4.

3aa as a mixture of isomers: **3-(4-(*tert*-Butyl)phenyl)-2-chloropyridine**, **5-(4-(*tert*-butyl)phenyl)-2-chloropyridine**, **4-(4-(*tert*-butyl)phenyl)-2-chloropyridine**, **2-(4-(*tert*-butyl)phenyl)-6-chloropyridine**

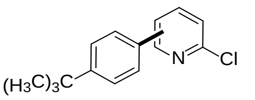

The title compound was prepared from 4-*tert*-butylbenzoic acid and 2-chloropyridine following general procedure A however, 2.0 equiv of **5** was used, (30.4 mg, 62%) as a mixture of C2/(C3+C4)/C5 isomers (37:22:41) as a colorless oil. All isomers could be separated by column chromatography, however determination of the isomeric ratio from analysis of the crude mixture did not allow for complete differentiation of the C3 and C4 isomers. Yield is the average from two reactions.

R_f = 0.5 (pentane); **3-(4-(*tert*-Butyl)phenyl)-2-chloropyridine ^{1}H NMR** (300 MHz, $CDCl_3$): δ (ppm): 7.93 (AA′BB′, *J* = 9.2 Hz, 2H), 7.72–7.65 (m, 1H), 7.63 (dd, *J* = 7.7, 1.2 Hz, 1H), 7.49 (AA′BB′, *J* = 9.2 Hz, 2H), 7.23 (dd, *J* = 7.5, 1.2 Hz, 1H), 1.37 (s, 9H); **^{13}C NMR** (75 MHz, $CDCl_3$): δ (ppm): 158.3, 153.0, 151.4, 139.3, 135.1, 126.9, 125.9, 122.3, 118.5, 34.8, 31.4; **5-(4-(*tert*-butyl)phenyl)-2-chloropyridine ^{1}H NMR** (300 MHz, $CDCl_3$): δ (ppm): 8.60 (dd, *J* = 2.6, 0.8 Hz, 1H), 7.83 (dd, *J* = 8.3, 2.6 Hz, 1H), 7.56–7.46 (m, 4H), 7.38 (dd, *J* = 8.3, 0.8 Hz, 1H), 1.36 (s, 9H); **^{13}C NMR** (75 MHz, $CDCl_3$): δ (ppm): 151.8, 148.0, 137.1, 133.7, 130.6, 126.8, 126.3, 126.0, 124.3, 34.8, 31.4; **4-(4-(*tert*-butyl)phenyl)-2-chloropyridine ^{1}H NMR** (300 MHz, $CDCl_3$): δ (ppm): 8.41 (dd, *J* = 5.2, 0.7 Hz, 1H), 7.60–7.49 (m, 5H), 7.43 (dd, *J* = 5.2, 1.6 Hz, 1H), 1.36 (s, 9H); **^{13}C NMR** (75 MHz, $CDCl_3$): δ (ppm): 153.3, 152.3, 151.5, 150.1, 134.0, 126.9, 126.4, 121.9, 120.4, 34.9, 31.4; **2-(4-(*tert*-butyl)phenyl)-6-chloropyridine ^{1}H NMR** (300 MHz, $CDCl_3$): δ (ppm): 8.38 (dd, *J* = 4.8, 2.0 Hz, 1H), 7.68 (dd, *J* = 7.6, 2.0 Hz, 1H), 7.48 (AA′BB′, *J* = 9.2 Hz, 2H), 7.40 (AA′BB′, *J* = 9.2 Hz, 2H), 7.30 (dd, *J* = 7.6, 4.8 Hz, 1H), 1.37 (s, 9H); **^{13}C NMR** (75 MHz, $CDCl_3$): δ (ppm): 151.5, 149.9, 148.2, 139.8, 137.0, 134.6, 129.1, 125.4, 122.7, 34.8, 31.5;

4-(*tert*-Butyl)-2-(4-(*tert*-butyl)phenyl)pyridine (3ab)[32]

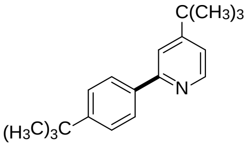

The title compound was prepared from 4-*tert*-butylbenzoic acid and 4-*tert*-butylpyridine following general procedure A however, 2.0 equiv of **5** was used, (31.5 mg, 59%) as a mixture of C2/C3 isomers (95:5) as a colorless oil. Yield is the average from two reactions. All data was consistent with that previously reported.[32]

IR (ATR): ν (cm^{-1}): 2963, 1609, 1466, 1434, 1270, 845, 733; **R_f** = 0.3 (pentane:EtOAc, 19:1); **^{1}H NMR** (300 MHz, $CDCl_3$): δ (ppm): 8.58 (dd, *J* = 5.3, 0.8 Hz, 1H), 7.90 (AA′BB′, *J* = 9.2 Hz, 2H), 7.69 (dd, *J* = 1.9, 0.8 Hz, 1H), 7.50 (AA′BB′, *J* = 9.2 Hz, 2H), 7.21 (dd, *J* = 5.3, 1.8 Hz, 1H), 1.36(5) (s, 9H), 1.36(2) (s, 9H); **^{13}C NMR** (75 MHz, $CDCl_3$): δ (ppm): 160.7, 157.6, 152.0, 149.6, 137.3, 126.9, 125.8, 119.2, 117.7, 35.0, 34.8, 31.5, 30.7.

3ac as a mixture of C2/C3/C4 isomers:[33] **2-Fluoro-4'-methoxy-1,1'-biphenyl**, **3-fluoro-4'-methoxy-1,1'-biphenyl**, **4-fluoro-4'-methoxy-1,1'-biphenyl**

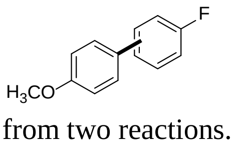

The title compound was prepared from 4-*tert*-butylbenzoic acid and fluorobenzene following general procedure A however, 2.0 equiv of **5** was used, (26.3 mg, 65%) as a mixture of C2/C3/C4 isomers (57:33:10) as colorless oil. Yield is the average from two reactions. All data was consistent with that previously reported.[33]

IR (ATR): ν (cm^{-1}): 2835, 1607, 1520, 1483, 1439, 1243, 1201, 1184, 833, 699; **R_f** = 0.4 (pentane:EtOAc, 19:1); **^{1}H NMR** (300 MHz, $CDCl_3$): δ (ppm): 7.55–7.49 (m, 2H), 7.47–7.07 (m, 4H), 7.04–6.96 (m, 2H), 3.86 (s, 3H) as a mixture of 3 compounds, 11H reported; **^{19}F NMR** (282MHz, $CDCl_3$): δ (ppm): -113.3, -116.7, -118.3.

6H-Benzo[c]chromen-6-one 6[34]

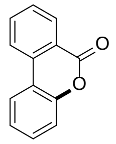

The title compound was prepared from [1,1'-biphenyl]-2-carboxylic acid following general procedure A (34.9 mg, 89%) as a white solid. Yield is the average from two reactions. All data was consistent with that previously reported.[34]

IR (ATR): ν (cm^{-1}): 3077, 3022, 1725, 1602, 1077, 745, 718, 681; $\mathbf{R_f}$ = 0.3 (pentane:EtOAc, 19:1); **^{1}H NMR** (300 MHz, $CDCl_3$): δ (ppm): 8.36 (ddd, *J* = 8.0, 1.6, 0.6 Hz, 1H), 8.10–8.05 (m, 1H), 8.01 (dd, *J* = 8.0, 1.6 Hz, 1H), 7.79 (ddd, *J* = 8.3, 7.3, 1.6 Hz, 1H), 7.55 (ddd, *J* = 8.3, 7.3, 1.1 Hz, 1H), 7.49–7.42 (m, 1H), 7.37–7.27 (m, 2H); **^{13}C NMR** (75 MHz, $CDCl_3$): δ (ppm): 161.2, 151.3, 134.9, 134.8, 130.6, 130.5, 128.9, 124.6, 122.8, 121.7, 121.3, 118.1, 117.8.

Phenyl 2-bromobenzoate (2b)

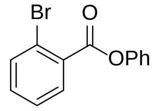

The title compound was isolated from the reaction of 2-bromobezoic acid and Br_2 in benzene. Compound **2b** was also synthesised independently from 2-bromobenzoic acid and phenol. To a stirred solution of 2-bromobenzoic acid (400 mg, 2.0 mmol, 1.0 equiv), phenol (376 mg, 4.0 mmol, 2.0 equiv) and 4-dimethylaminopyridine (200 mg, 1.6 mmol, 0.8 equiv) in methylene chloride (5 mL) at 0 °C was added a solution of N,N'-dicyclohexylcarbodiimide (460 mg, 2.2 mmol, 1.1 equiv) in methylene chloride (5 mL). The reaction was stirred at 0 °C for 2 h, then warmed to room temperature and filtered through a pad (1 cm) of Celite®, and washed with methylene chloride. The filtrate was washed with HCl (10 mL of a 1M aq. solution), $NaHCO_3$ (10 mL of a sat. aq. solution), brine (10 mL), dried with Na_2SO_4, filtered and concentrated. Purification by flash column chromatography (pentane/EtOAc, 9:1) provided the title compound (362 mg, 62%) as a clear oil.

IR (ATR): ν (cm^{-1}): 3067, 1748, 1589, 1489, 1285, 1242, 1188, 1022, 741, 687; $\mathbf{R_f}$ = 0.3 (pentane:EtOAc, 19:1); **^{1}H NMR** (300 MHz, $CDCl_3$): δ (ppm): 8.06–7.99 (m, 1H), 7.79–7.72 (m, 1H), 7.51–7.38 (m, 4H), 7.34–7.24 (m, 3H); **^{13}C NMR** (75 MHz, $CDCl_3$): δ (ppm): 164.7, 150.8, 134.8, 133.3, 131.9, 131.5, 129.7, 127.5, 126.3, 122.4, 121.7; **HRMS** (ESI): m/z calculated for $[C_{13}H_9Br^{79}NaO_2^+]$ [M+Na]: 298.9678, measured 298.9683.

8. Mechanistic studies

8a) Stern-Volmer luminescence quenching experiments

Stern-Volmer luminescence quenching studies were carried out using a 2×10^{-7} M solution of $[Ir(dF(CF_3)ppy)_2(dtbbpy)](PF_6)$ (**PC**) and variable concentrations of bromomalonate **5**, 2-bromobenzoic acid, a mixture of 2-bromobenzoic aicd and Cs_2CO_3, and tetrabutylammonium benzoate, in degassed benzene under Ar atmosphere. The samples were prepared in 1.4 mL quartz cuvettes, equipped with PTFE stoppers, and sealed with Parafilm M® inside an argon filled glove-box. The intensity of the emission peak at 472 nm (λ_{ex} = 420 nm) expressed as the ratio I_0/I, where I_0 is the emission intensity of **PC** at 472 nm in the absence of a quencher and I is the observed intensity, as a function of the quencher concentration was measured. Stern–Volmer plots for each component, along with all plots on one graph, are given in Figure S4 below.

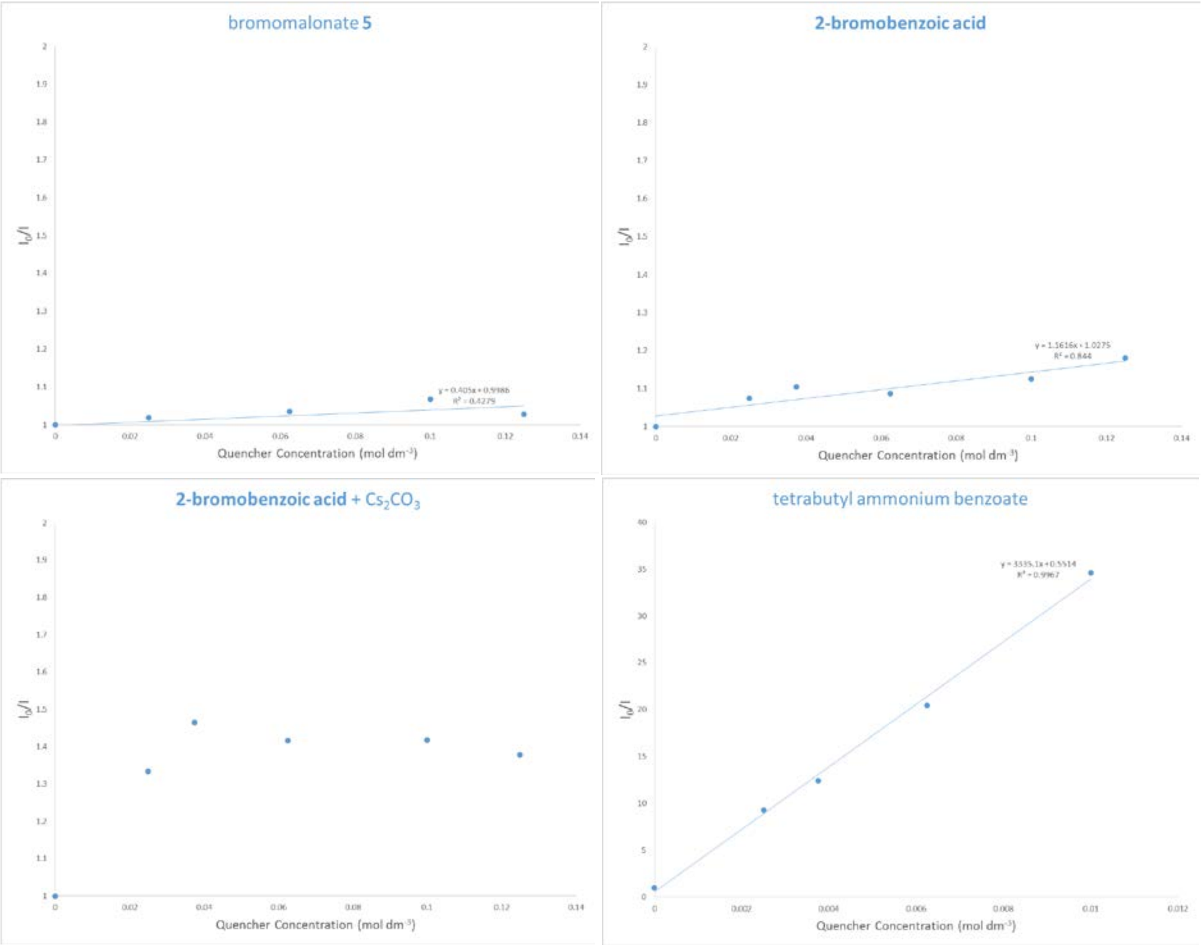

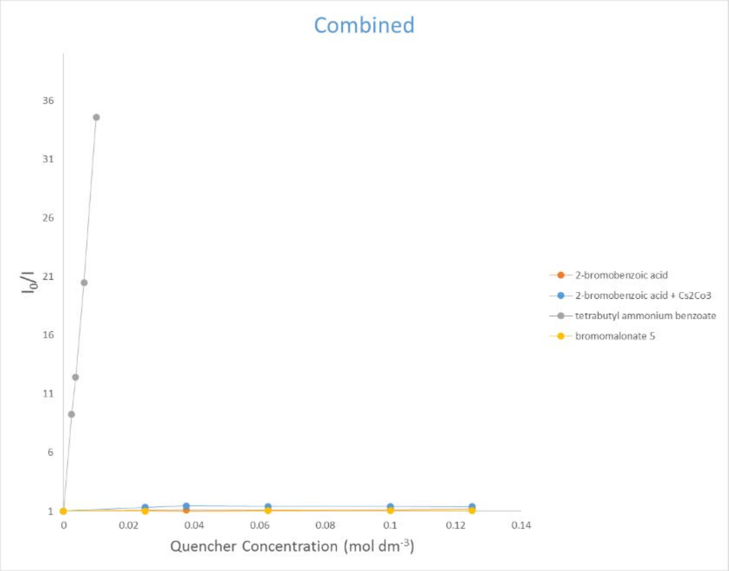

Figure S4. Stern-Volmer luminescence quenching plots examining the 472 nm emission of **PC** in benzene (0.2 µM). The quenching efficiency of bromomalonate **5**, 2-bromobenzoic acid, 2-bromobenzoic acid and Cs_2CO_3 combined, and tetrabutylammonium benzoate are represented. Additionally, all four plots are combined on one graph.

Issues arose when trying to measure luminescence quenching of 2-bromobenzoic acid and Cs_2CO_3 combined. The mixture was quite insoluble in benzene. In an initial attempt to measure luminescence quenching of the mixture, the compounds were sonicated in benzene and then the heterogeneous solution was filtered through a syringe tip filter disc. While this allowed measurement of what was in solution, we could not quantify the concentration of the cesium carboxylate. Additionally, during attempts to measure quenching, the carboxylate was observed to precipitate from the solution, further changing the concentration of the solution (see graph 2-bromobenzoic acid + Cs_2CO_3). To avoid these issues, luminescence quenching studies of tetrabutylammonium benzoate were instead undertaken.

To verify that the tetrabutylammonium cation was not quenching **PC** (rather than the benzoate anion), the luminescence quenching of tetrabutylammonium hydrogen sulfate was studied. While it was found to be a moderate quencher of **PC** (Stern Volmer constant = 12.7 $mol^{-1}dm^3$), it was not as efficient a quencher as tetrabutylammonium benzoate (Stern Volmer constant = 3335 $mol^{-1}dm^3$) (Figure S5).

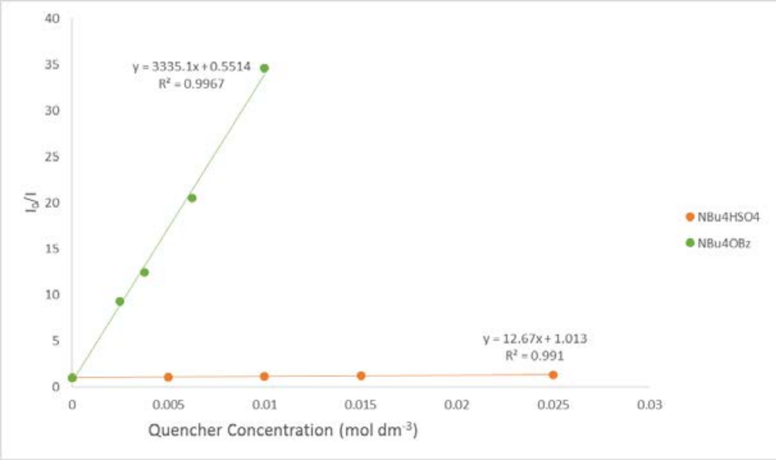

Figure S5. Stern-Volmer luminescence quenching plot examining the 472 nm emission of **PC** in benzene (0.2 µM). The quenching efficiency of tetrabutylammonium hydrogen sulfate and tetrabutylammonium benzoate.

8b) Quantum yield measurements

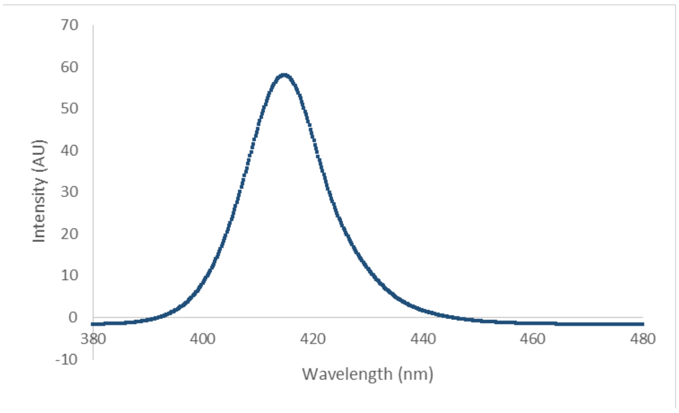

Figure S6. Emission spectrum of blue LED used for quantum yield experiments (λ_{max} = 415 nm)

Determination of the light intensity at 415 nm:

Determination of the quantum yield of the reaction was undertaken using a blue LED (λ_{max} = 415 nm) as the I_{max}(**PC**) = 380 nm.

According to the procedure of Yoon,[35] the photon flux of the LED (λ_{max} = 415 nm) was determined by standard ferrioxalate actinometry.[36,37] A 0.15 M solution of ferrioxalate was prepared by dissolving potassium ferrioxalate hydrate (0.737 g) in H_2SO_4 (10 mL of a 0.05 M solution). A buffered solution of 1,10-phenanthroline was prepared by dissolving phenanthroline (25 mg) and sodium acetate (5.63 g) in H_2SO_4 (25 mL of a 0.5 M solution). Both solutions were stored in the dark. To determine the photon flux of the LED, the ferrioxalate solution (1.0 mL) was placed in a cuvette and irradiated for 90 seconds at λ_{max} = 415 nm. After irradiation, the phenanthroline solution (0.175 mL) was added to the cuvette and the mixture was allowed to stir in the dark for 1 hour to allow the ferrous ions to completely coordinate to the phenanthroline. The absorbance of the solution was measured at 510 nm. A non-irradiated sample was also prepared and the absorbance at 510 nm was measured. Conversion was calculated using eq 5.

$$\text{mol } Fe^{2+} = \frac{V \bullet \Delta A}{l \bullet \varepsilon} \qquad (5)$$

Where V is the total volume (0.001175 L) of the solution after addition of phenanthroline, ΔA is the difference in absorbance at 510 nm between the irradiated and non-irradiated solutions, 1 is the path length (1.000 cm), and ε is the molar absorptivity at 510 nm (11,100 L $mol^{-1}cm^{-1}$). The photon flux can be calculated using eq 6.

$$\text{Photon flux} = \frac{\text{mol } Fe^{2+}}{\Phi \bullet t \bullet f} \qquad (6)$$

Where Φ is the quantum yield for the ferrioxalate actinometer (1.1 for a 0.15 M solution at λ = 420 nm),[35] t is the time (90.0 s), and f is the fraction of light absorbed at λ = 420 nm (eq 7).

The absorbance of the above ferrioxalate solution at 420 nm was measured to be >3 indicating the fraction of light absorbed is >0.999. The fraction of light absorbed (f) by this solution was calculated using eq 7, where A is the measured absorbance at 420 nm.

$$f = 1 - 10^{-A} \qquad (7)$$

The photon flux was calculated (average of three experiments) to be 3.06 x 10^{-9} einsteins s^{-1}.

Determination of quantum yield:

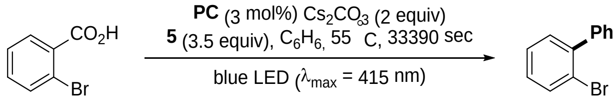

3b

To a screw-cap vial was added Cs_2CO_3 (0.6 mmol, 2 equiv), carboxylic acid (0.2 mmol, 1 equiv), **PC** (3 mol%), bromomalonate **5** (0.70 mmol, 3.5 equiv), benzene (5.0 mL), and decane (0.2 mmol, 1 equiv) as internal standard. A needle was then inserted into the septum and the solution was purged with argon for 10 minutes. After this time, 1 mL of the reaction mixture was taken, and filtered through a syringe-tip filter (to remove any solids) into a quartz cuvette. The cuvette was capped with a PTFE stopper and sealed with Parafilm M®. The sample was stirred and irradiated (λ_{max} = 415 nm) for 33390 sec. After irradiation, the yield of product formed was determined by GC-MS analysis to be 23% based on the decane standard. The quantum yield was determined using eq 8. Essentially all incident light (f > 0.999) is absorbed by **PC** under the reaction conditions described.

$$\Phi = \frac{\text{mol prod}}{\text{flux} \bullet \text{t} \bullet \text{f}} \qquad (8)$$

The quantum yield Φ(6%) for the reaction was calculated to be 0.14.

8c) Reaction kinetics

The reaction of 2-bromobenzoic acid with benzene was run as per general procedure A, using decane (1 equiv) as an internal standard to enable analysis of the yield using a calibrated GC-MS. The experimental values are an average of two experimental runs.

Table S3. Yield of the reaction with respect to time.

time (h)	yield (%)
1	5.3
2	12.2
3	19.7
4	27.1
5	33
6	39.7
7	46.2
22	89.7

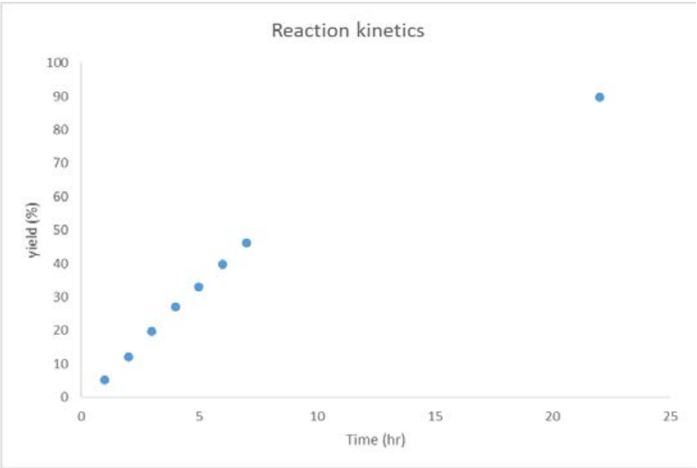

Chart S2. Plot of the yield of the reaction with respect to time.

8d) Analysis of radical intermediates

To determine whether the reaction was proceeding via the intermediacy of an aryl radical, the selectivity of the reaction with a monosubstituted arene was studied. It was reasoned that if the reaction was proceeding via the aryl radical, then the selectivity obtained would match the selectivity observed for the same aryl radical reacting with the monosubstituted arene. To this end, 4-methoxybenzoic acid was reacted with fluorobenzene under the standard reaction conditions (eq. 9). Following the reaction, the ratio of C2/C3/C4 isomers was determined from the crude reaction mixture using ^{19}F NMR spectroscopic analysis to be 1.0:0.58:0.17 (=57:33:10).

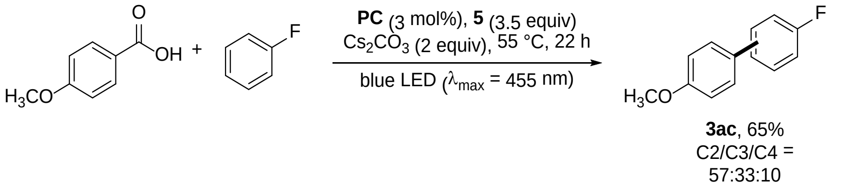

(9)

The isomeric ratio of the biaryl product was compared to those reported by Studer and co-workers for the base promoted homolytic aromatic substitution of 4-iodoanisol with fluorobenzene. The reported C2/C3/C4 isomeric ratio was reported to be identical (C2/C3/C4 = 1.0:0.58:0.17) to our observed selectivity.[33]

8e) Investigation of alternative reaction pathways

To determine whether bromomalonate **5** was acting as an oxidant for the PC, the reaction of benzoic acid 4a was undertaken under the standard reaction conditions, however **5** was replaced with ammonium persulfate (eq. 10) . When the reaction was heated to 55 °C only 1% of biaryl 3b was observed. When the same reaction was performed at 130 °C the product was formed in 27%.

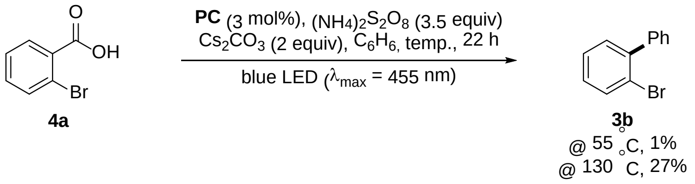

(10)

To ensure that ammonium persulfate is a competent oxidant at 55 °C, the reaction of acid 4b was undertaken. Lactone 6 was afforded in 34% yield (eq. 11).

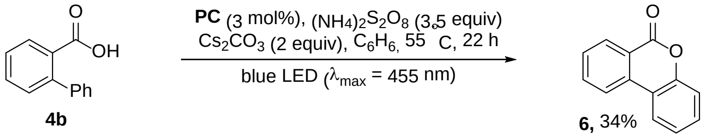

(11)

Crossover experiments

Experiment 1: Investigation into whether the aryl bromide is an intermediate.

While unlikely due to redox potentials of the **PC** and aryl bromides, it was confirmed that the reaction was not proceeding via decarboxylative bromination, followed by single electron reduction of the resultant aryl bromide. This was achieved by performing a crossover experiment. 2-Bromobenzoic acid was reacted in benzene under the standard reaction conditions in the presence of aryl bromide **S6** (eq. 12). It was proposed that if the reaction was proceeding via the formation of an intermediate aryl bromide, then the formation of biaryl product resulting from the reaction of **S6** with benzene would be observed. These cross-products were not detected, hence this result in combination with the reported redox potentials of **PC** and aryl bromides, suggests that aryl bromides are not intermediates in this reaction.

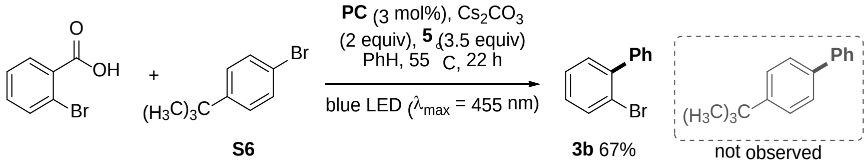

(12)

Experiment 2: Investigation into whether aryl benzoate is an intermediate.

A second cross-over experiment was undertaken exclude the possibility that benzoate esters, such as **2b**, are intermediates in the biaryl synthesis. Benzoate ester **2b** was observed in the thermolysis of acyl hypobromite **1** in benzene, and was detected as a by-product of the decarboxylation reaction during optimization studies. Thus, benzoate **2b** and 2-chlorobenzoic acid were reacted under the optimized reaction conditions (eq. 13). While 2-chlorobenzoic acid was found to provide biaryl **3d** in acceptable yield, reaction of benzoate **2b** to provide biaryl **3b** was not observed suggesting that phenyl benzoates are not intermediates in the reaction.

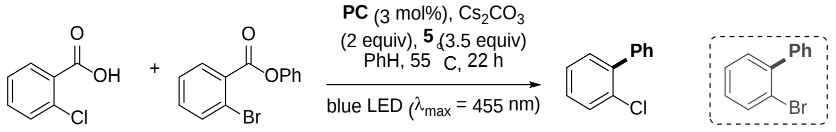

2b 3d 64% 3b not observed (13)

8f) Qualitative detection of CO_2

Experiments to validate the extrusion of CO_2 were undertaken. This was complicated because the optimal base for the reaction is Cs_2CO_3, and when this deprotonates the carboxylic acid, CO_2 is formed. To allow us to detect the CO_2 formed from the decarboxylation of the benzoic acid, benzoic acid-α-^{13}C **S1d** (99 atom % ^{13}C) was employed in the reaction, and the extrusion of ^{13}C CO_2 was analyzed by ^{13}C NMR spectroscopy.

To analyze the ^{13}C-CO_2 formed in the reaction, the reaction was performed in a sealed J. Young® NMR tube. As a consequence of this, the reaction could not be stirred, and therefore a solubility was a key concern. To overcome solubility issues, and eliminate other sources of CO_2 in the reaction, tetrabutylammonium benzoate-α-^{13}C was employed (eq. 14).

To a 8 mL screw-capped vial with septum (not dried), [Ir(dF(CF_3)ppy)$_2$(dtbbpy)](PF_6) (**PC**) (3 mol%), carboxylate **S7** (0.1 mmol, 1.0 equiv), C_6D_6 (2.0 mL) and diethyl 2-bromo-2-methylmalonate (**5**) (0.35 mmol, 3.5 equiv) were added. A needle was then inserted into the septum and the solution was purged with argon for 10 minutes. Following this, an aliquot (1 mL) of the reaction mixture was transferred to the NMR tube and the reaction was heated to 80 °C in a silicon oil bath while irradiated with 34W Blue LED lamp (Kessil KSH150B LED Grow Light) for 22 h. Following the reaction, ^{13}C NMR spectroscopic analysis of the crude mixture was performed and a signal for CO_2 was clearly observed at 124.8 ppm (Figure S7). This was in accordance with the reported chemical shift of CO_2 in C_6D_6.[38]

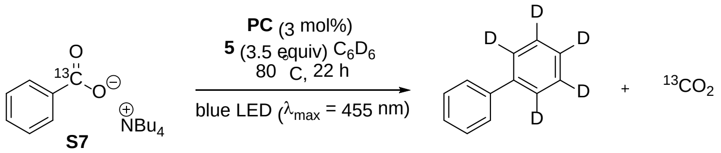

S7 (14)

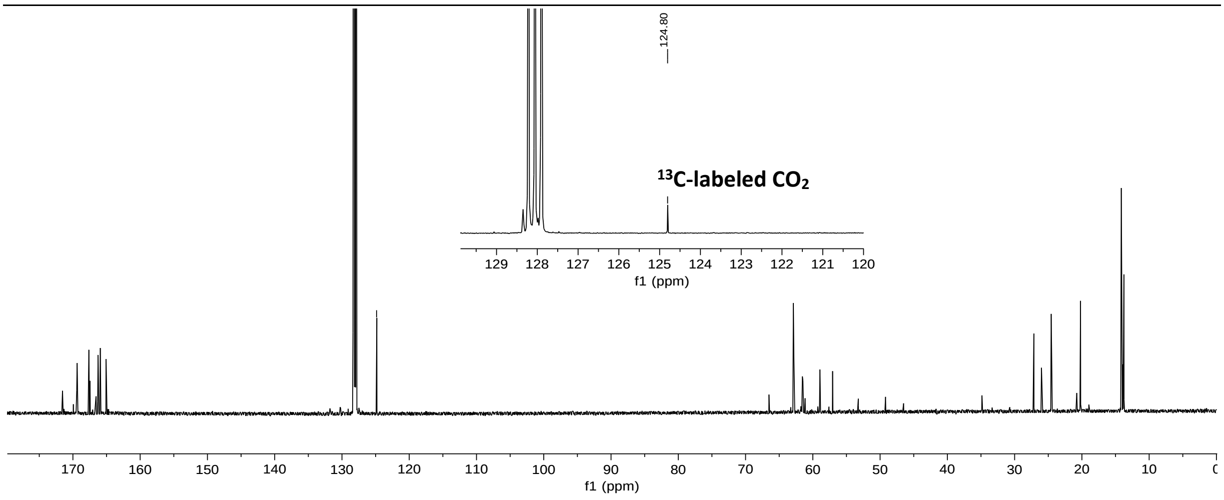

Figure S7. ^{13}C NMR of reaction mixture following the extrusion of ^{13}C-labeled CO_2 (151 MHz, C_6D_6).

8g) Theoretical computations for the decarboxylation of III

Considering the lack of precedent mechanism for the decarboxylation of a hypobromite radical anion, we were interested to see if a plausible transition state could be located with DFT computations.

The computations were carried out with the Gaussian 09 suite at the B2PLYP-D3/6-311+G(d,p)//B3LYP/6-31+G(d,p) level including IEFPCM(benzene) to account for solvation. The optimization was carried out without symmetrical or internal constraints and frequencies were calculated at the same level as the optimization to confirm the absence of imaginary frequencies for minima and the presence of a single imaginary frequency for the transition state. Natural population analysis was performed with the Gaussian NBO version 3.1 module.

The decarboxylation of the hypobromite radical anion can occur through a viable transition state with ΔG = 21.5 kcal mol–1 relative to **III**, featuring concerted C–C and O–Br cleavages. Natural population analysis revealed a delocalization of the extra charge and spin over Br and the connecting O in **III**. Furthermore, the products of the concerted dissociation of **III** are phenyl radical, neutral CO_2 and Br^- and the electronic structure in the transition state already reflects this charge and spin distribution (eq 15). When considering the possibility of a non-concerted decarboxylation pathway, rather a stepwise mesoylsis of O–Br bond followed by breaking of the C–C bond, it was found that mesolysis of the O–Br bond of **III** would provide benzoate anion and a bromide radical. However, no transition state for this mesolysis could be located.

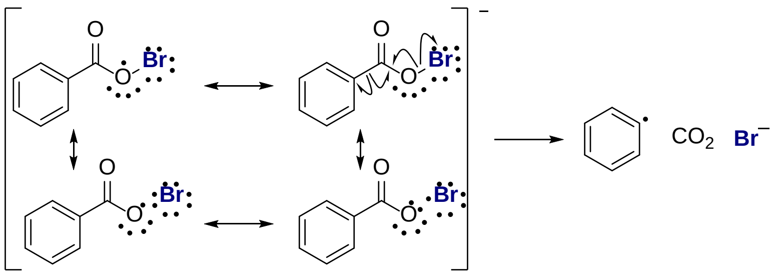

(15)

Minimum energy structures of the discussed ground state of III and the transition state for the decarboxylation of III. The numbers are charge and spin distribution by NPA.

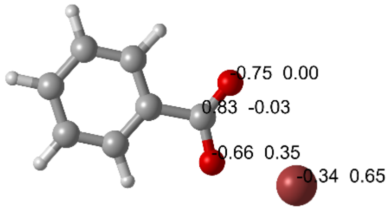

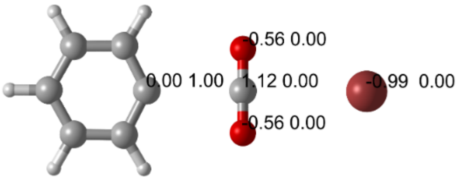

III **III TS**

Cartesian coordinates of minimum energy structures

III

Number of imaginary frequencies = 0

HF(B3LYP/6-31+G(d,p)) = -2992.068357

G(B3LYP/6-31+G(d,p)) = -2992.002955

MP2(B2PLYP/6-311+G(d,p)) = -2993.736826

-1 2

C	4.24534	-0.43777	-0.01888
C	3.81090	0.89209	-0.04512
C	2.44495	1.18541	-0.02815
C	1.49920	0.15233	0.01144
C	1.93879	-1.17879	0.03513
C	3.30484	-1.47257	0.02230
H	5.30817	-0.66556	-0.03089
H	4.53737	1.70023	-0.07872
H	2.08914	2.21033	-0.04624
H	1.19776	-1.96978	0.06442
H	3.63528	-2.50804	0.04364
C	0.00887	0.50327	0.02695
O	-0.32479	1.69555	0.07719
O	-0.74673	-0.54090	-0.01718
Br	-3.19180	-0.16766	-0.01298

III TS

Number of imaginary frequencies = 1

HF(B3LYP/6-31+G(d,p)) = -2992.021919

G(B3LYP/6-31+G(d,p)) = -2991.962608

MP2(B2PLYP/6-311+G(d,p)) = -2993.696400

-1 2

C	-4.67409	-0.00016	0.00019
C	-3.97848	1.21390	0.00005
C	-2.57377	1.22492	-0.00014
C	-1.93414	0.00005	-0.00020
C	-2.57358	-1.22492	-0.00007
C	-3.97829	-1.21412	0.00012
H	-5.76049	-0.00025	0.00036
H	-4.52206	2.15564	0.00011
H	-2.01388	2.15435	-0.00023
H	-2.01354	-2.15426	-0.00012
H	-4.52172	-2.15594	0.00022
C	0.20894	0.00022	-0.00009
O	0.43875	1.16811	-0.00002
O	0.43878	-1.16766	-0.00018
Br	3.68091	-0.00007	0.00006

10. References

1. M. A. Ishay, Z. Lu and T. P. Yoon, *J. Am. Chem. Soc.* **2010**, *132*, 8572.
2. D. P. Rillema, G. Allen, T. J. Meyer and D. Conrad, *Inorg. Chem.* **1983**, *22*, 1617.
3. a) S. Sprouse, K. A. King, P. J. Spellane and R. J. Watts, *J. Am. Chem. Soc.* **1984**, *106*, 6647; b) J. D. Slinker, A. A. Gorodetsky, M. S. Lowry, J. Wang, S. Parker, R. Rohl, S. Bernhard and G. G. Malliaras, *J. Am. Chem. Soc.* **2004**, *126*, 2763.
4. D. Hanss, J. C. Freys, G. Bernardinelli and O. S. Wegner, *Eur. J. Inorg. Chem.* **2009**, 4850.
5. T. Voigt, C. Gerding-Reimers, T. T. Ngoc Tran, S. Bergmann, H. Lachance, B. Schölermann, A. Brockmeyer, P. Janning, S. Ziegler and H. Waldmann, H. *Angew. Chem. Int. Ed.* **2013**, *52*, 410.
6. A. C. Spivey L. Shukla and J. F. Hayler *Org. Lett.* **2007**, *9*, 891.
7. M. D. Elliot and C. J. Goddard *Synth. Commun.* **1989**, *19*, 1505.
8. K. Chen and P. S. Baran, *Nature* **2009**, *459*, 824.
9. F. Estevan, S. Ibáñez, A. Ofori, P. Hirva, M. Sanaú and M. A. Úbeda, *Eur. J. Inorg. Chem.* **2015**, 2822.
10. Predicted NMR data calculated using Advanced Chemistry Development, Inc. (ACD/Labs) Software V11.01 (© 1994-2016 ACD/Labs).
11. M. Srebnik, *Synth. Commun*. **1989**, *19*, 197, and references therein.
12. A. Kumar and B. A. Shah, *Org. Lett*. **2015**, *17*, 5232.
13. J. Yu, J. Liu, G. Shi, C. Shao and Y. Zhang, *Angew. Chem. Int. Ed*. *2015*, **54**, 4079.
14. W.-J. Zhou, K.-H. Wang and J.-X. Wang, *Adv. Synth. Catal*. **2009**, *351*, 1378.
15. I. Bonnaventure and A. B. Charette, *J. Org. Chem*. **2008**, *73*, 6330.
16. J. L. Bolliger and C. M. Frech, *Adv. Synth. Catal*. **2010**, *352*, 1075.
17. . J. Kan, S. Huang, J. Lin, M. Zhang and W. Su, *Angew. Chem. Int. Ed*. **2015**, *54*, 2199.
18. F.-X. Felpin and E. Fouquet, *Adv. Synth. Catal*. **2008**, *350*, 863.
19. L. Bai and J.-L. Wang, *Adv. Synth. Catal*. **2008**, *350*, 315.
20. H. Li, C.-L. Sun, M. Yu, D.-G. Yu, B.-J. Li and Z.-J. Shi, *Chem. Eur. J*. **2011**, *17*, 3593.
21. E. Alacid and C. Nájera, *Org. Lett*. **2008**, *10*, 5011
22. S. Thapa, P. Basnet, S. K. Gurung and R. Giri, *Chem. Commun*. **2015**, *51*, 4009.
23. S.-M. Wang, X.-Y. Wang, H.-L. Qin and C.-P. Zhang, *Chem. Eur. J*. **2016**, *22*, 6542.
24. S. L. Gibson, J. J. Holt, M. Ye, D. J. Donnelly, T. Y. Ohulchanskyy, Y. You and M. R. Detty, *Bioorg. Med. Chem. Lett*. **2005**, *13*, 6394.
25. G. Cahiez, D. Luart and F. Lecomte, *Org. Lett*. **2004**, *6*, 4395.
26. G. Karig, J. A. Spencer and T. Gallagher, *Org. Lett*. **2001**, *3*, 835.
27. Y. Liang, W. Geng, J. Wei, K. Ouyanga and Z. Xi, *Org. Biomol. Chem.* **2012**, *10*, 1537.
28. D. Leifert and A. Studer, *Org. Lett*. **2015**, *17*, 386.
29. T. Heesgaard Jepsen, M. Larsen, M. Jørgensen, K. A. Solank, A. D. Bond, A. Kadziola and M. Brøndsted Nielsen, *Eur. J. Org. Chem*, **2011**, 23.
30. L. Lunazzi, M. Mancinelli, A. Mazzanti, S. Lepri, R. Ruzziconi and M. Schlosser, *Org. Biomol. Chem.* **2012**, *10*, 1847.
31. O. Kobayashi, D. Uraguchi and T. Yamakawa, *Org. Lett*. **2009**, *12*, 2679.
32. M. S. Lowry, W. R. Hudson, R. A. Pascal Jr. and S. Bernhard, *J. Am. Chem. Soc*. **2004**, *126*, 14129.
33. A. Dewanji, S. Murarka, D. P. Curran and A. Studer, *Org. Lett*. **2013**, *15*, 6102.
34. N. P .Ramirez, I. Bosque and J. C. Gonzalez-Gomez, *Org. Lett*. **2015**, *17*, 4550.
35. M. A. Cismesia and T. P. Yoon, *Chem. Sci*. **2015**, *6*, 5426.
36. C. G. Hatchard and C. A. Parker, *Proc. Roy. Soc. (London)* **1956**, *A23*, 518.
37. (a) H. J. Kuhn, S. E. Braslavsky and R. Schmidt, *Pure Appl. Chem*. **2004**, *76*, 2105; (b) M. Monalti, *et. al*. Chemical Actinometry. *Handbook of Photochemistry*, 3rd Ed; Taylor & Francis Group, LLC. Boca Raton, FL, **2006**, 601.
38. G. R. Fulmer, A. J. M. Miller, N. H. Sherden, H. E. Gottlieb, A. Nudelman, B. M. Stoltz, J. E. Bercaw and K. I. Goldberg, *Organometallics* **2010**, *29*, 2176.

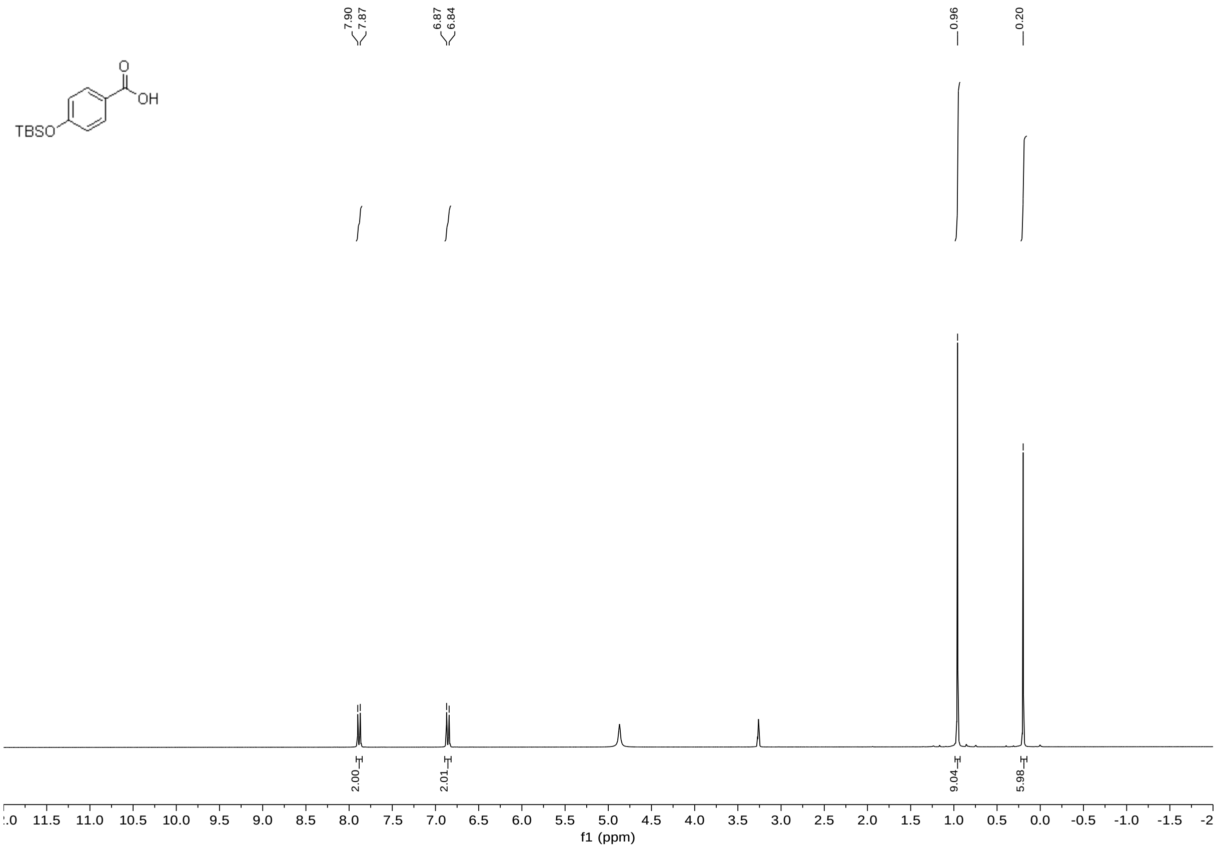

7.90
7.87
6.87
6.84
0.96
0.20
OH
TBSO
2.00
2.01
9.04
5.98
f1 (ppm)

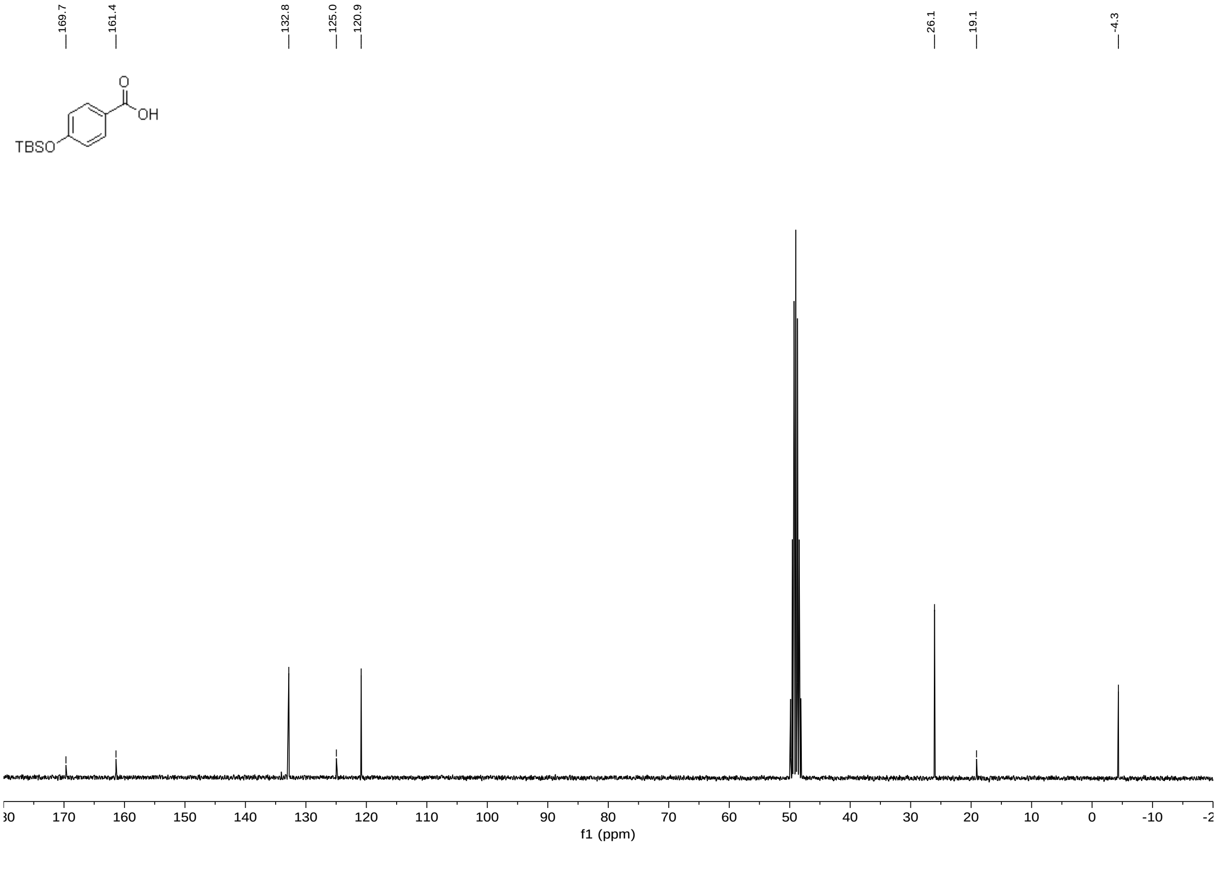

169.7
161.4
132.8
125.0
120.9
26.1
19.1
-4.3
OH
TBSO
f1 (ppm)

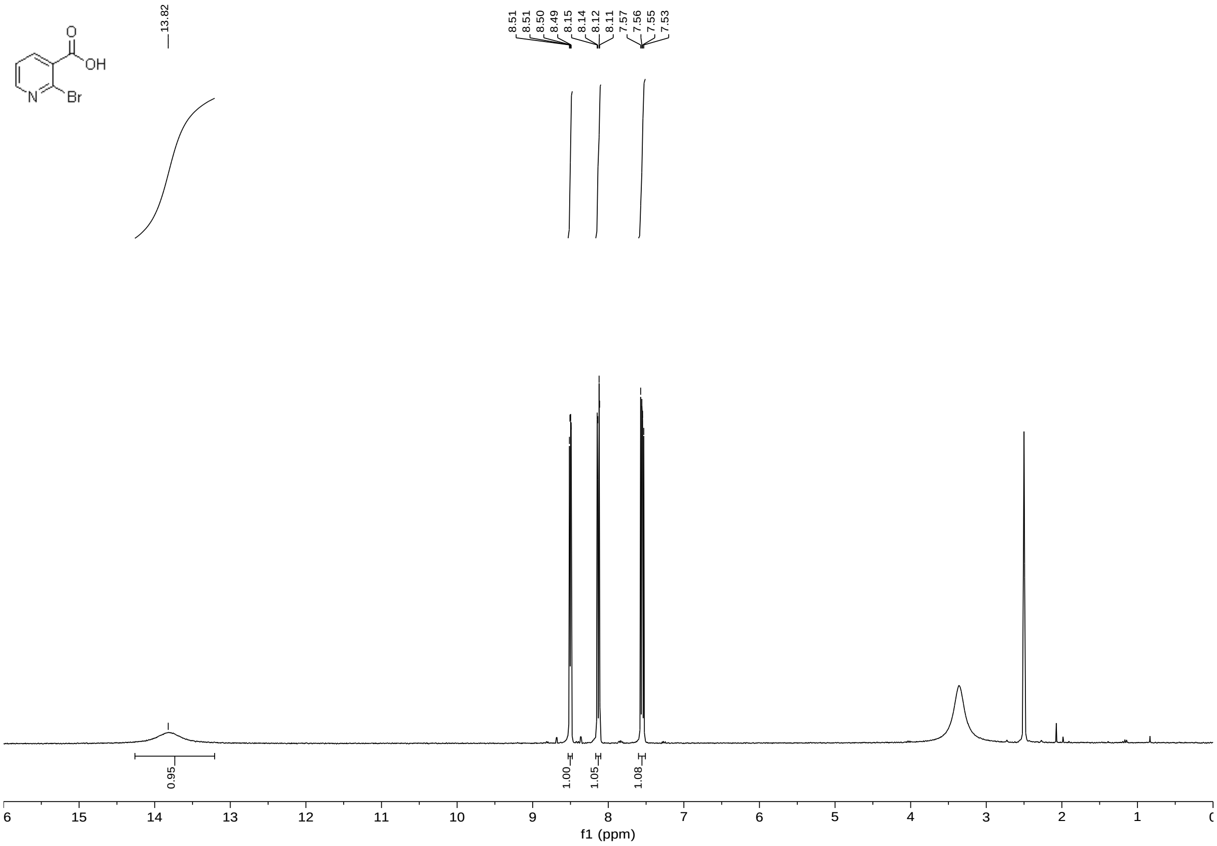
O
OH
N
Br
13.82
8.51
8.51
8.50
8.49
8.15
8.14
8.12
8.11
7.57
7.56
7.55
7.53
0.95
1.00
1.05
1.08
f1 (ppm)

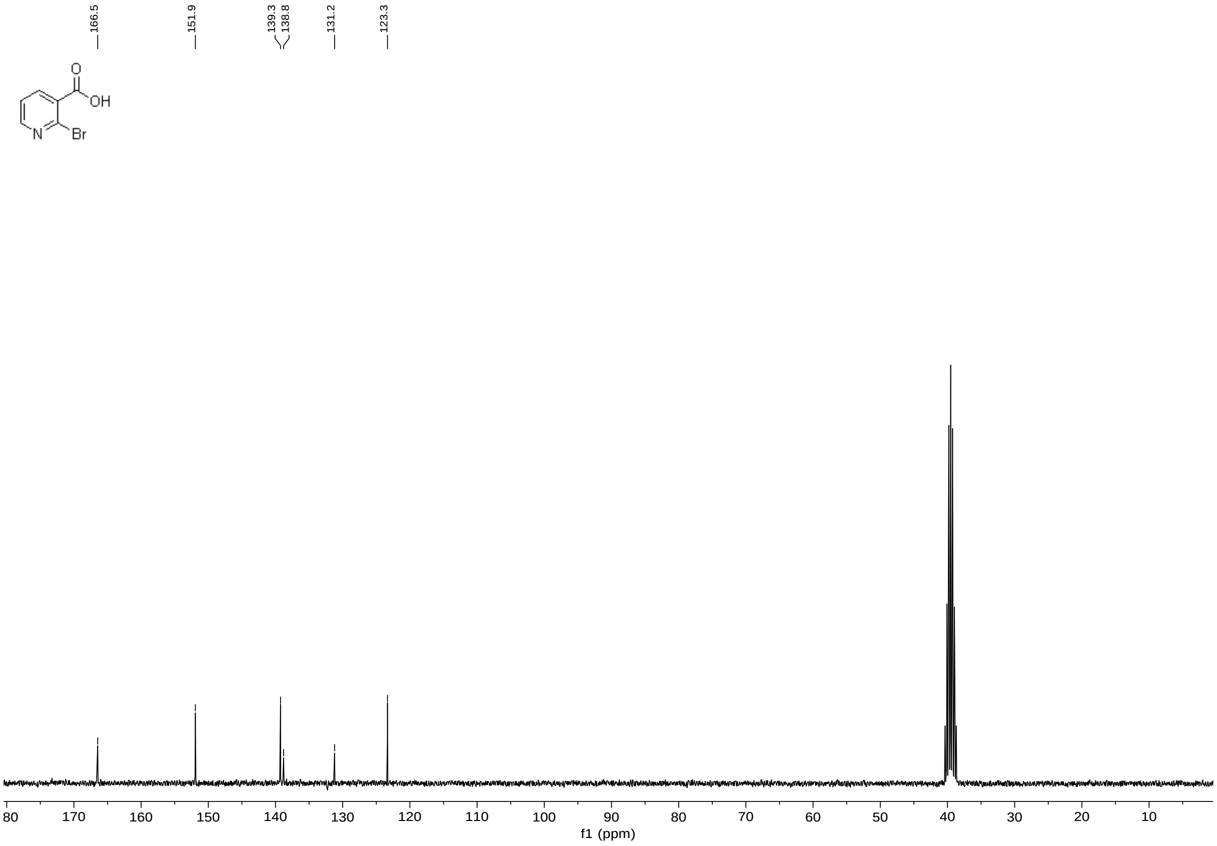
166.5
151.9
139.3
138.8
131.2
123.3
O
OH
N
Br
f1 (ppm)

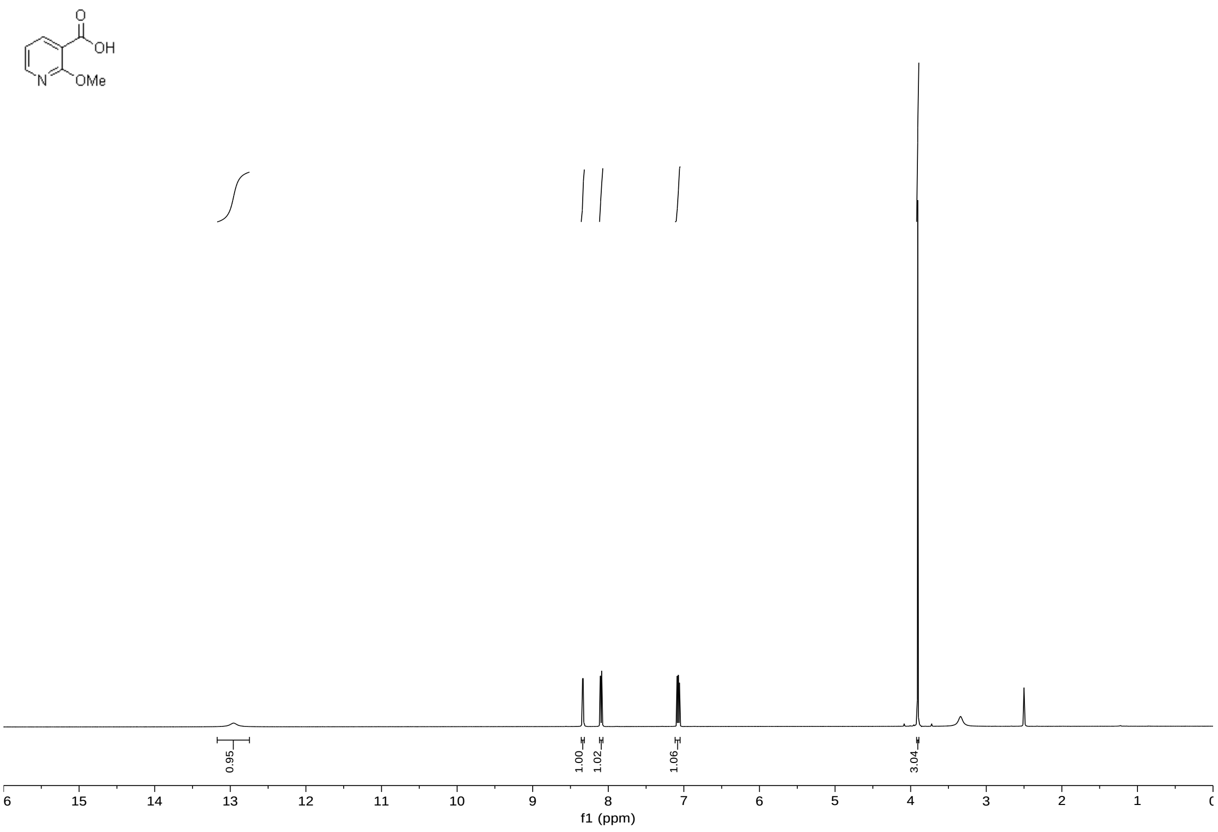
OH
OMe
N
O
0.95
1.00
1.02
1.06
3.04
f1 (ppm)

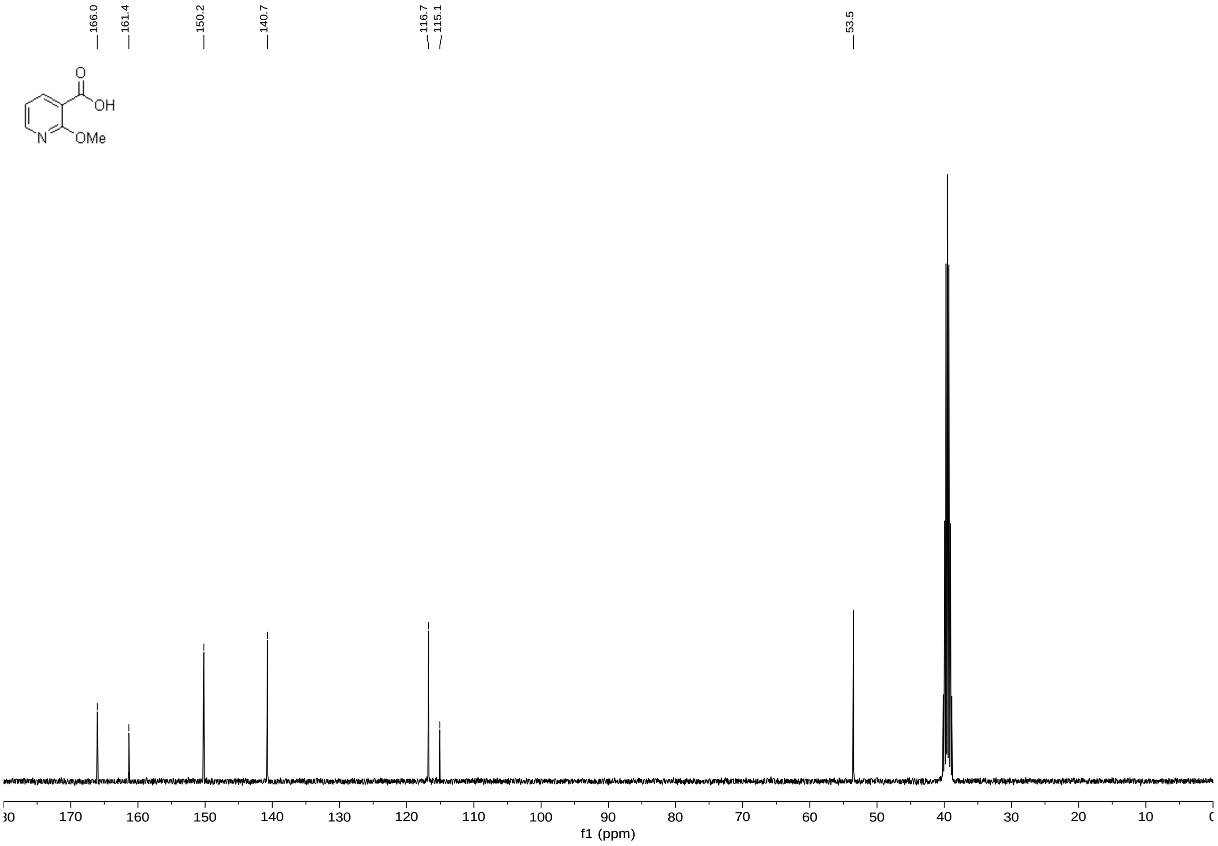
166.0
161.4
150.2
140.7
116.7
115.1
53.5
OH
OMe
N
O
f1 (ppm)

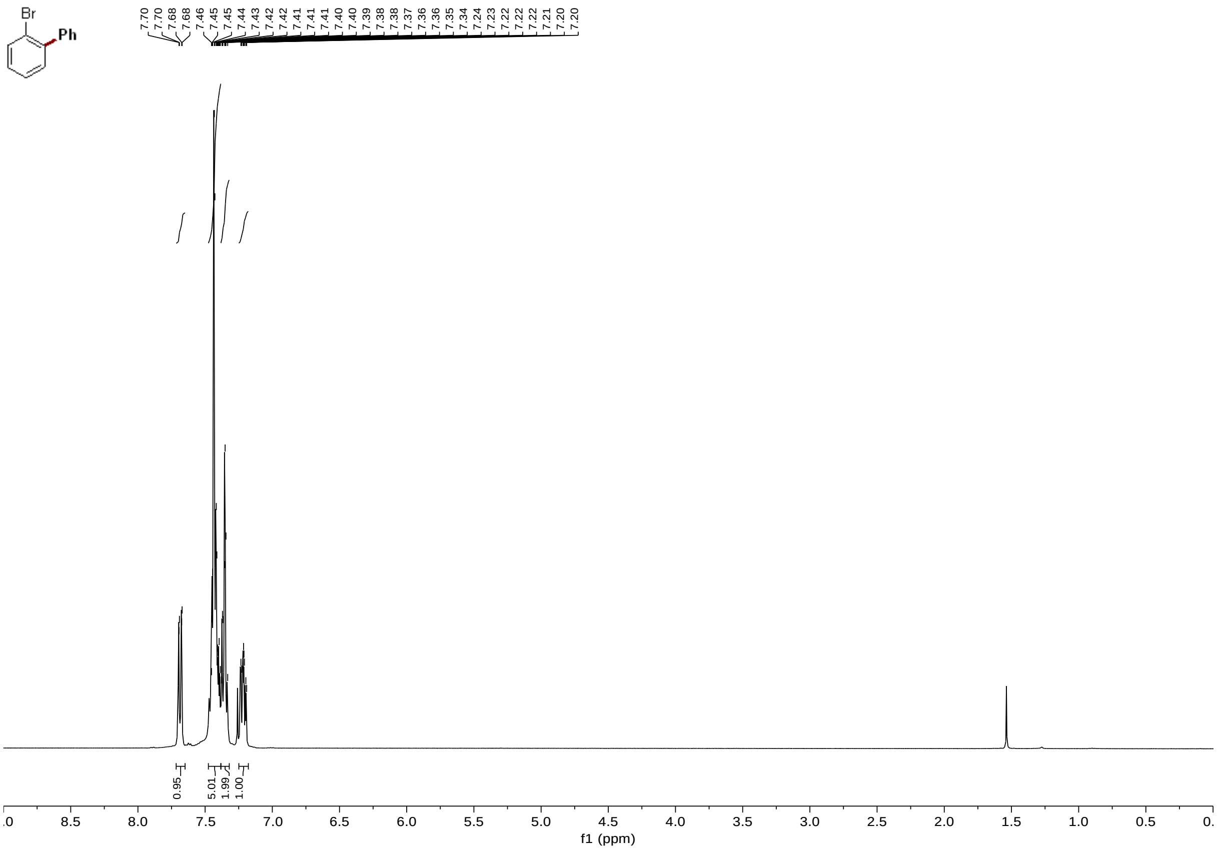
Br
Ph
7.70
7.70
7.68
7.68
7.46
7.45
7.45
7.44
7.43
7.42
7.42
7.41
7.41
7.41
7.40
7.40
7.39
7.38
7.38
7.37
7.36
7.36
7.35
7.34
7.24
7.23
7.22
7.22
7.22
7.21
7.20
7.20
0.95
5.01
1.99
1.00
f1 (ppm)

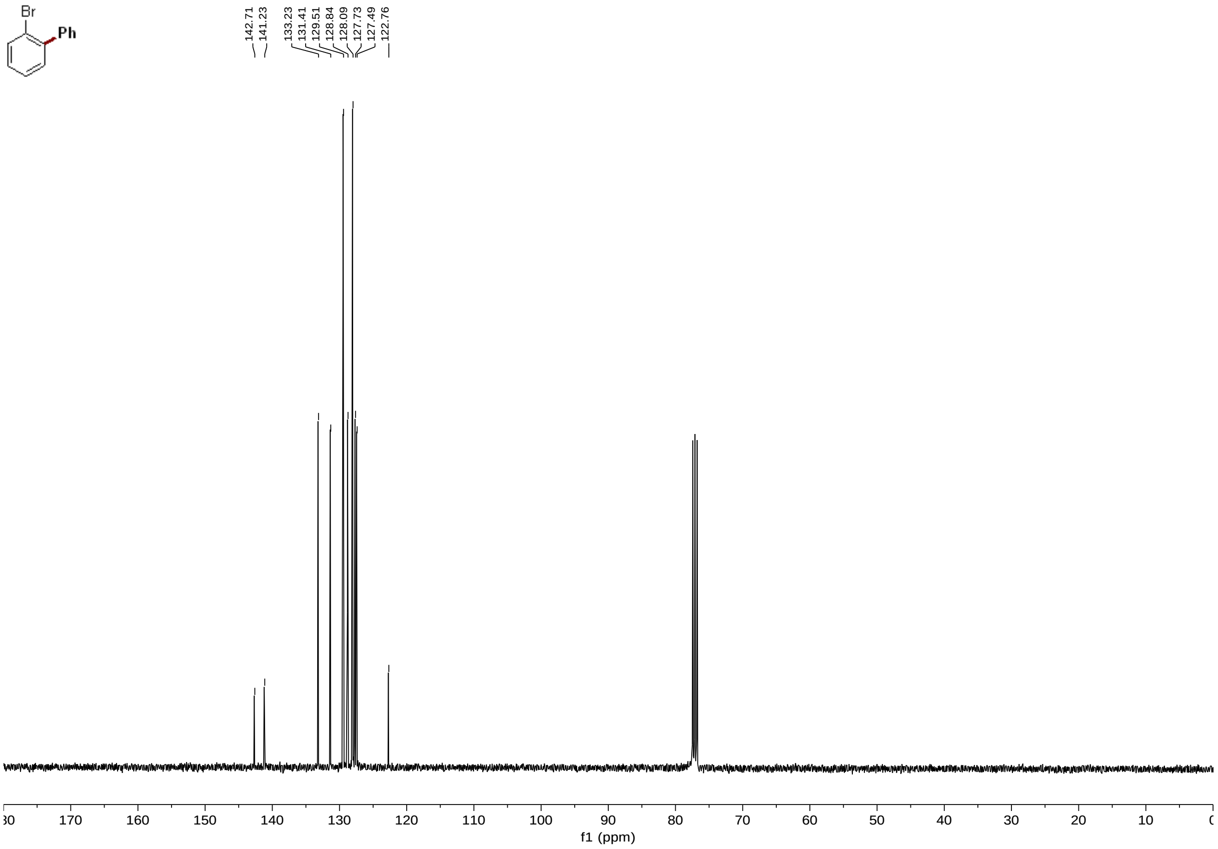
Br
Ph
142.71
141.23
133.23
131.41
129.51
128.84
128.09
127.73
127.49
122.76
f1 (ppm)

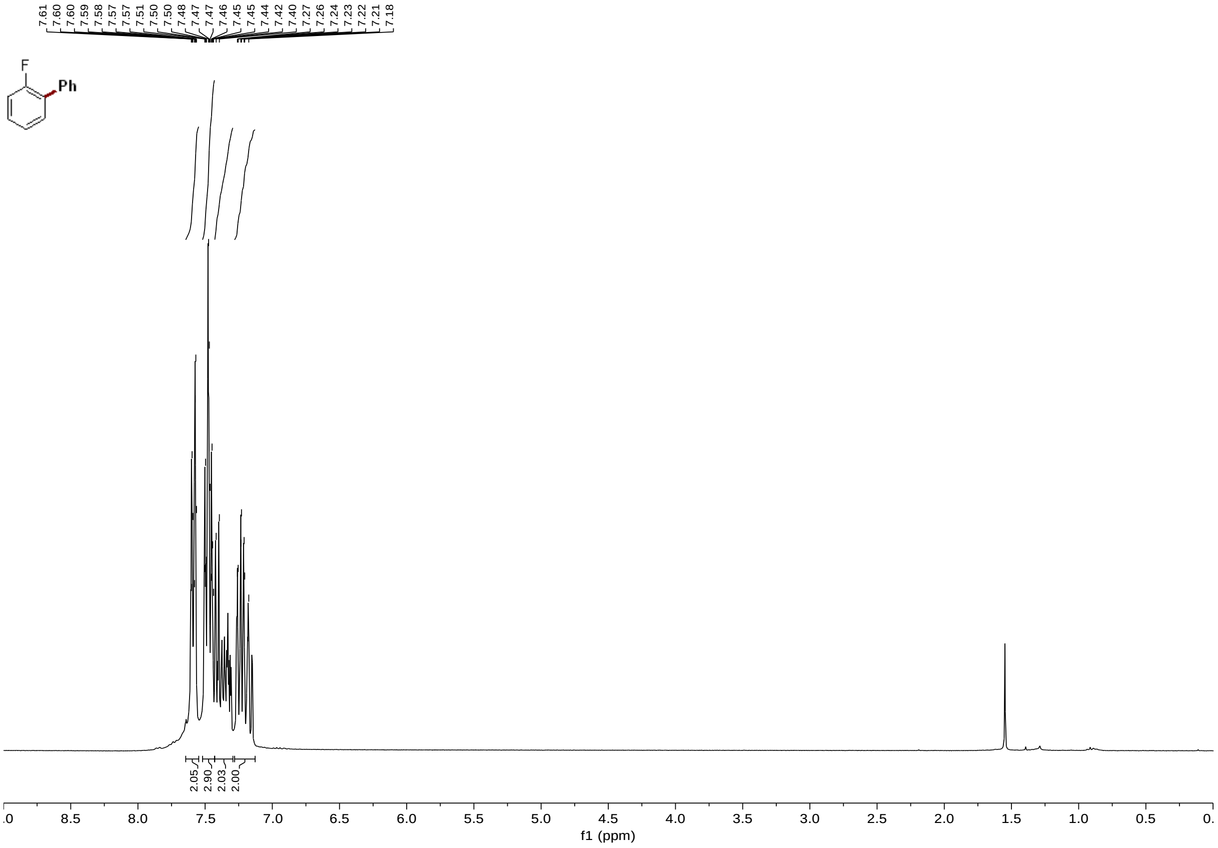

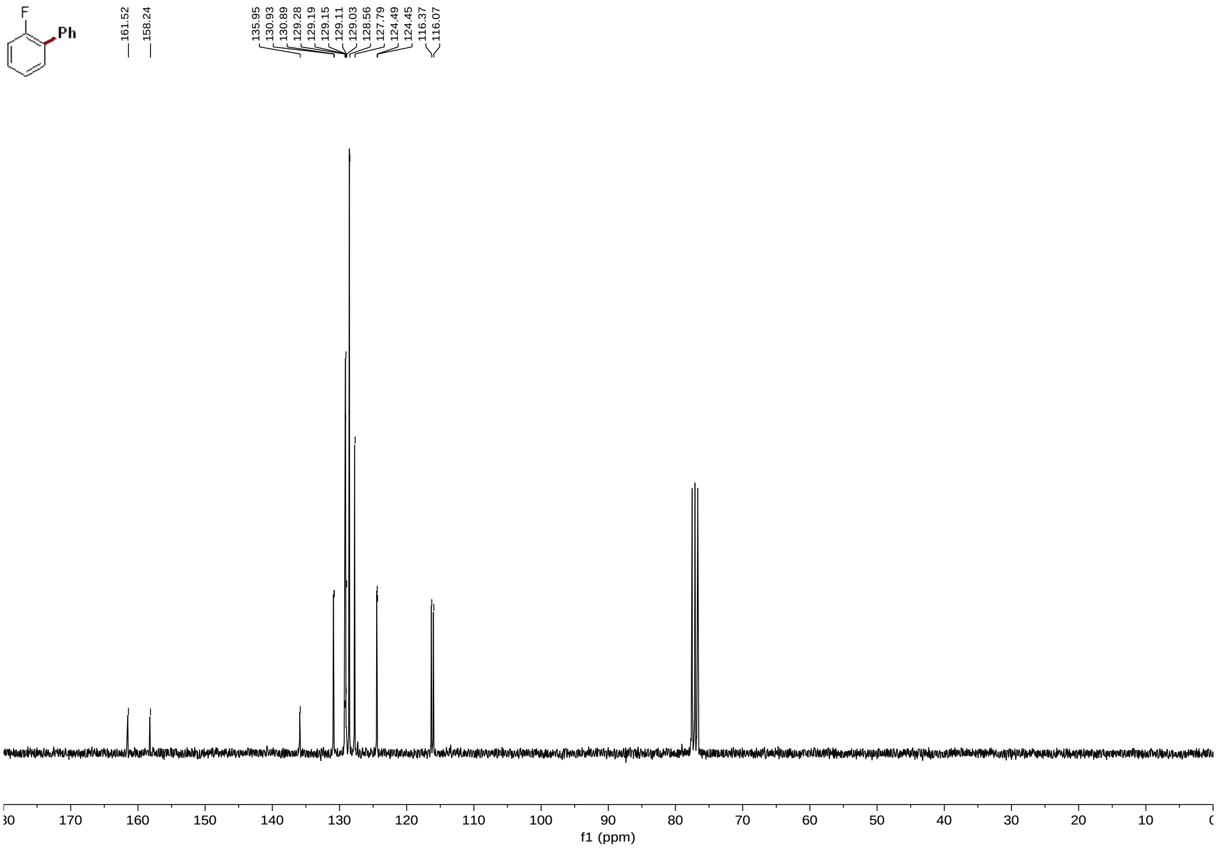

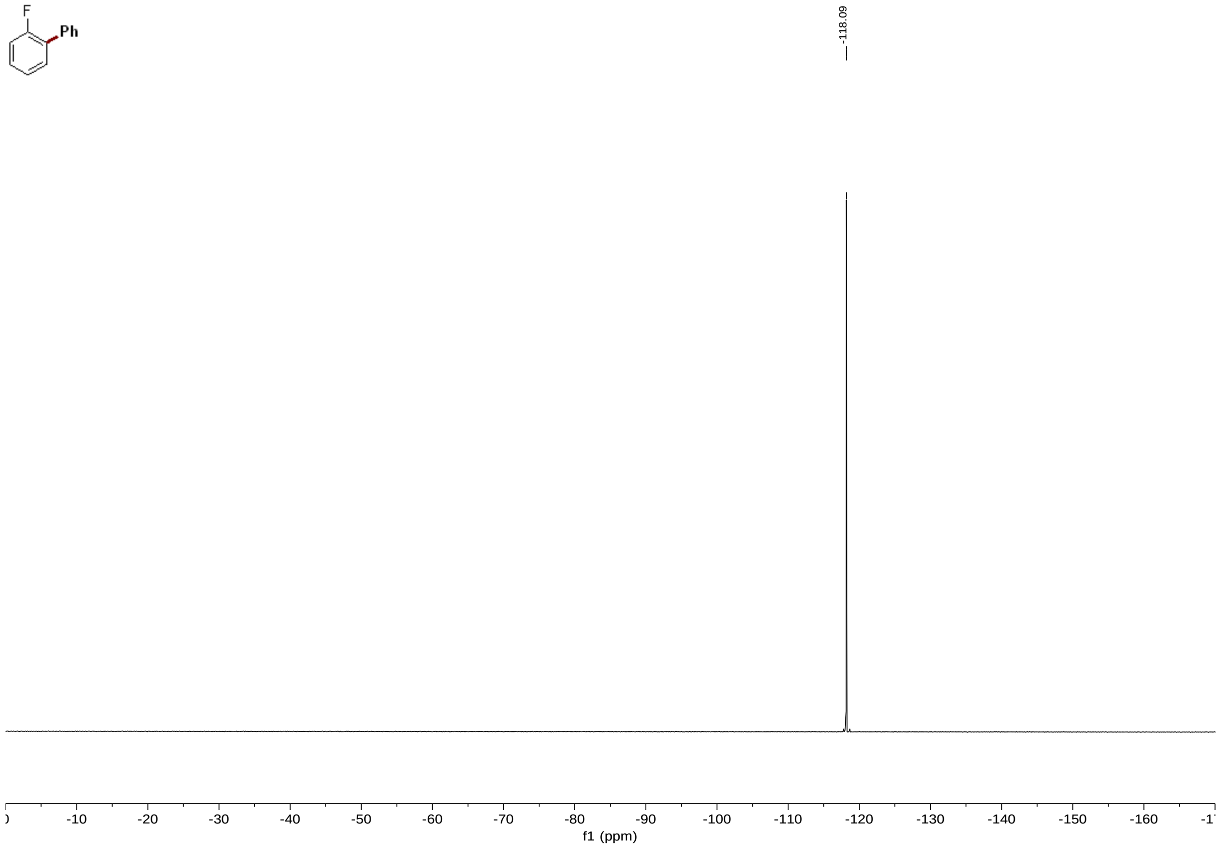

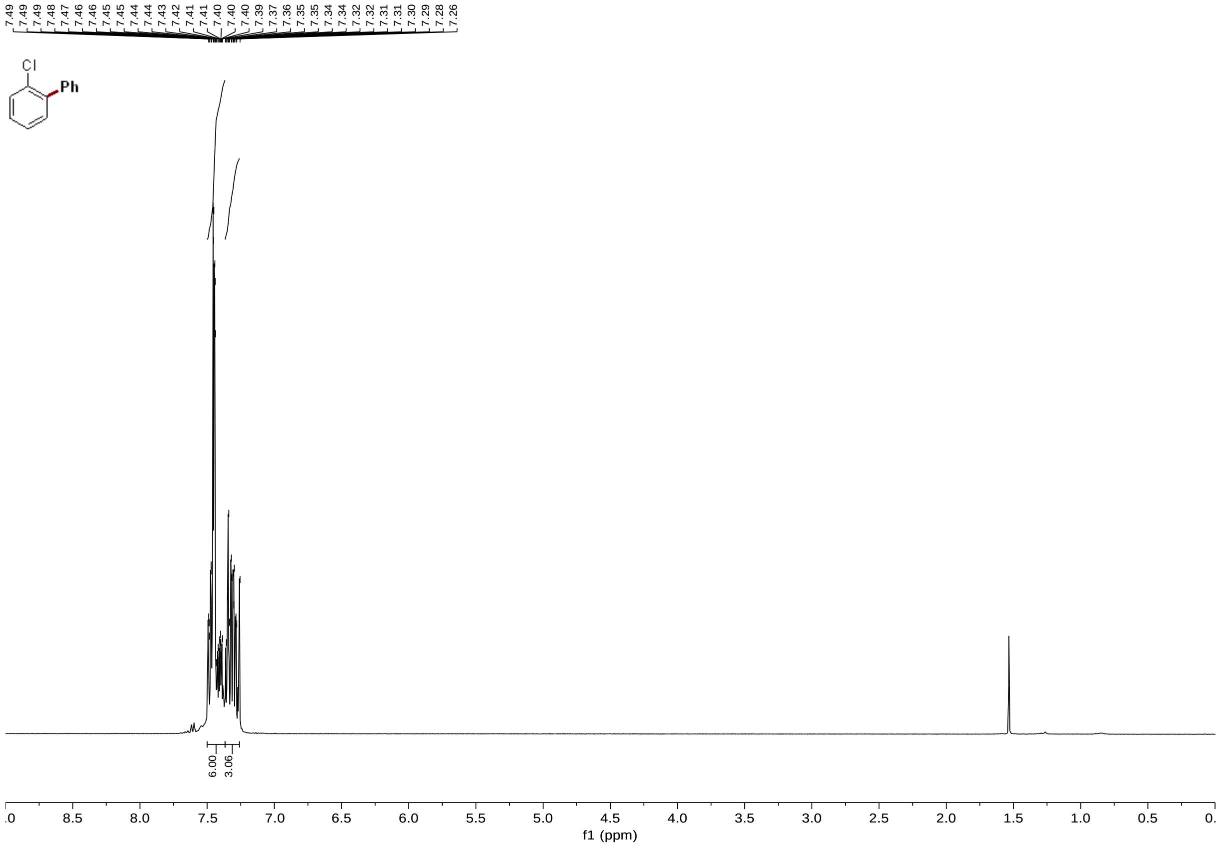

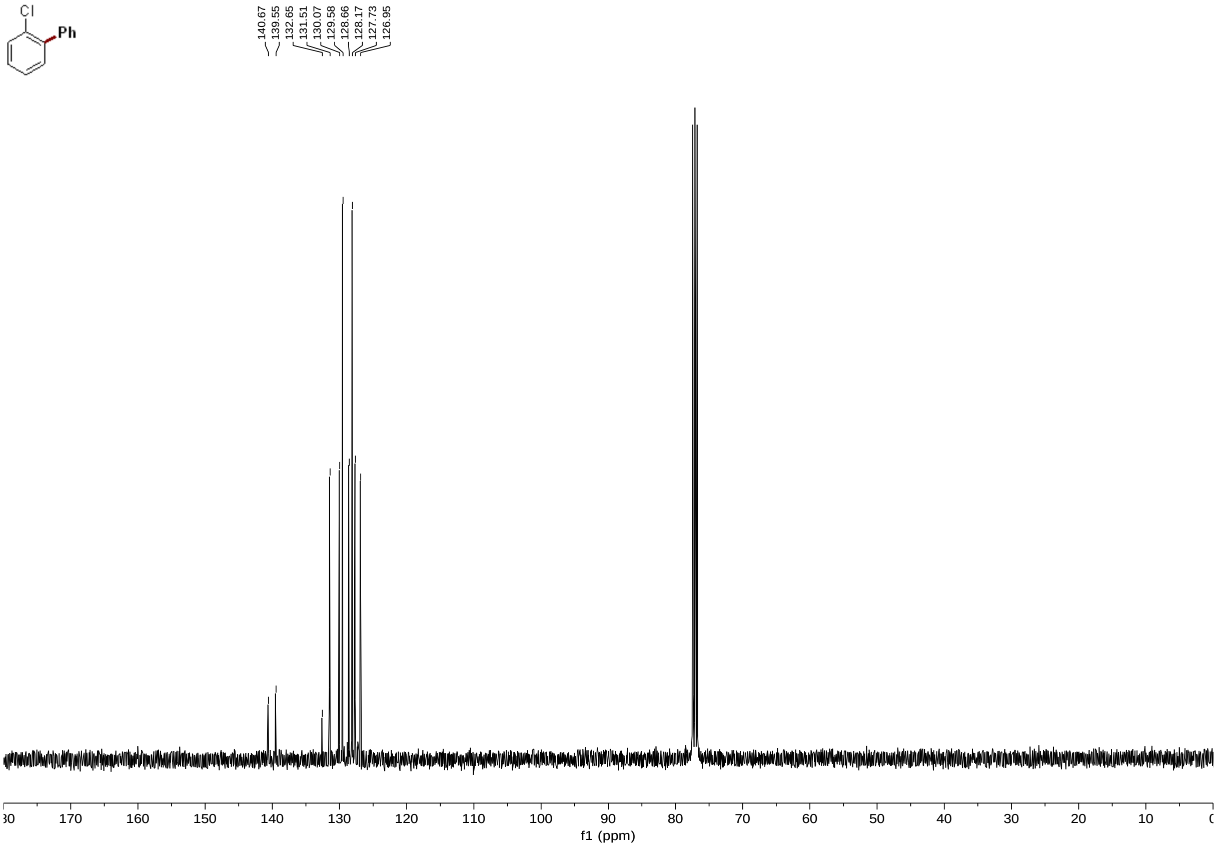
Cl
Ph
140.67
139.55
132.65
131.51
130.07
129.58
128.66
128.17
127.73
126.95
f1 (ppm)

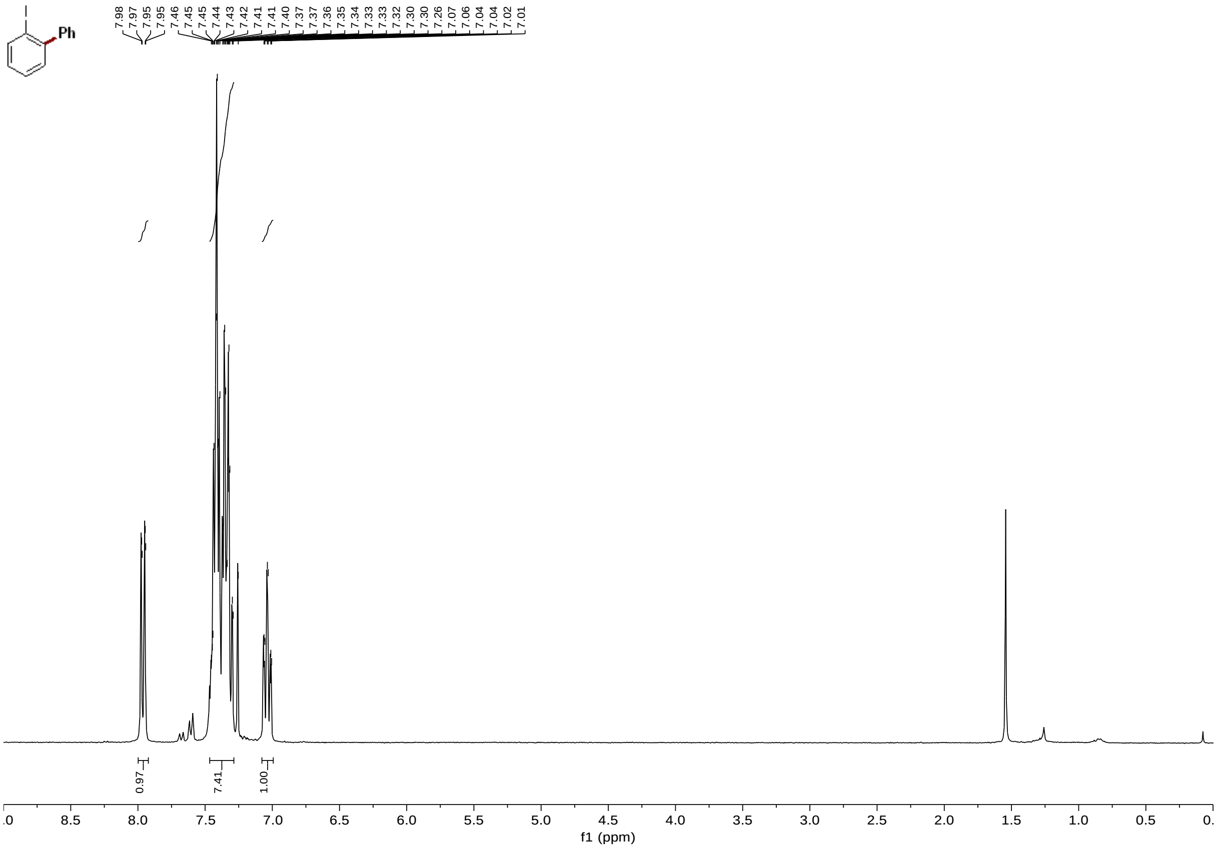
I
Ph
0.97
7.41
1.00
f1 (ppm)

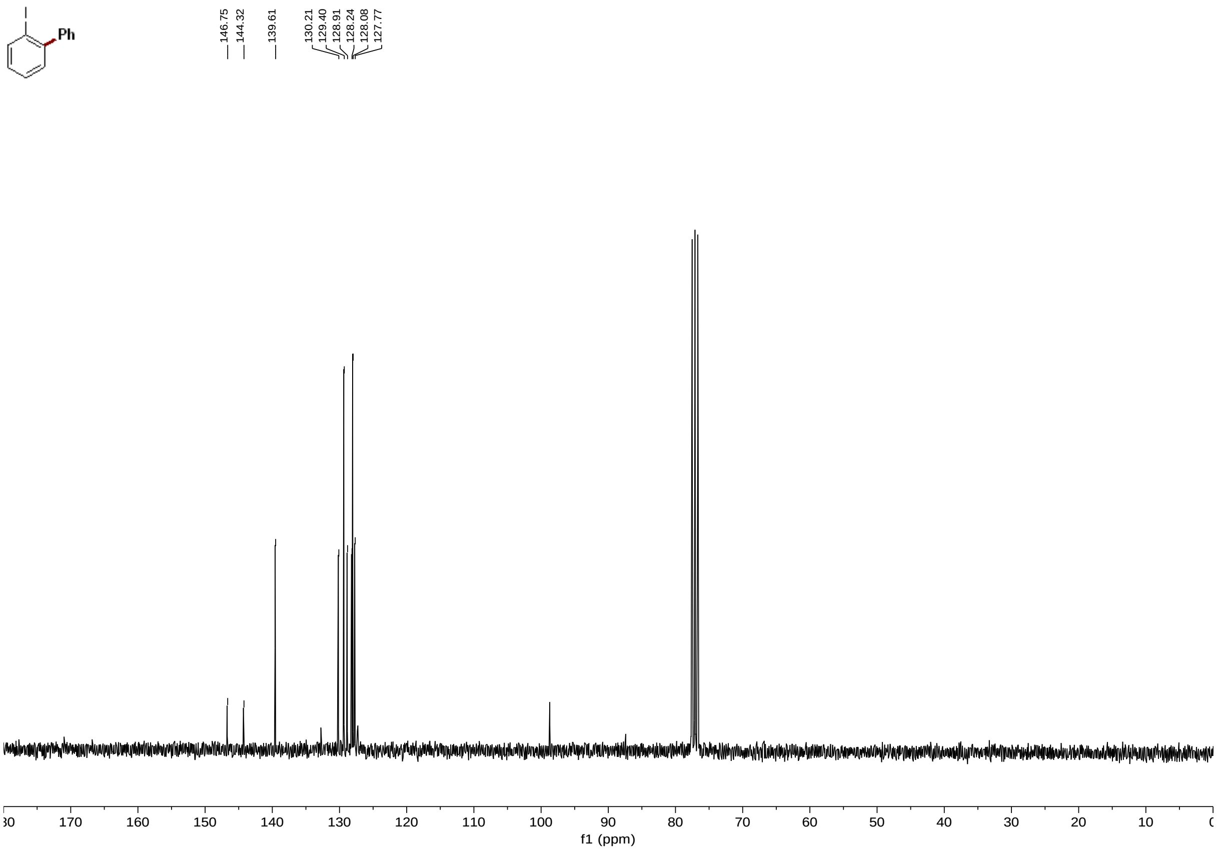
Ph
146.75
144.32
139.61
130.21
129.40
128.91
128.24
128.08
127.77
f1 (ppm)

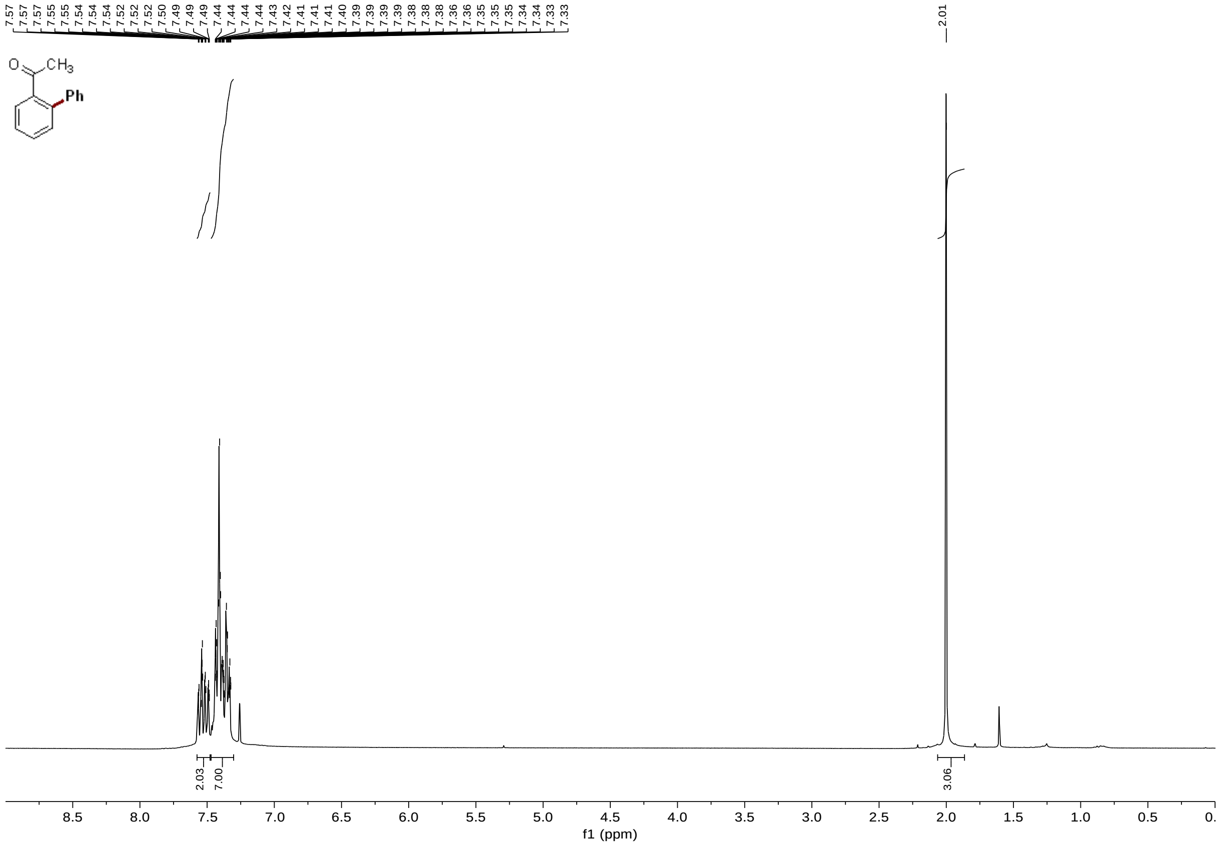
O
CH_3
Ph
7.57
7.57
7.57
7.55
7.55
7.54
7.54
7.54
7.52
7.52
7.52
7.50
7.49
7.49
7.49
7.44
7.44
7.44
7.44
7.43
7.42
7.41
7.41
7.41
7.40
7.39
7.39
7.39
7.39
7.38
7.38
7.38
7.36
7.36
7.35
7.35
7.35
7.34
7.34
7.33
7.33
2.01
2.03
7.00
3.06
f1 (ppm)

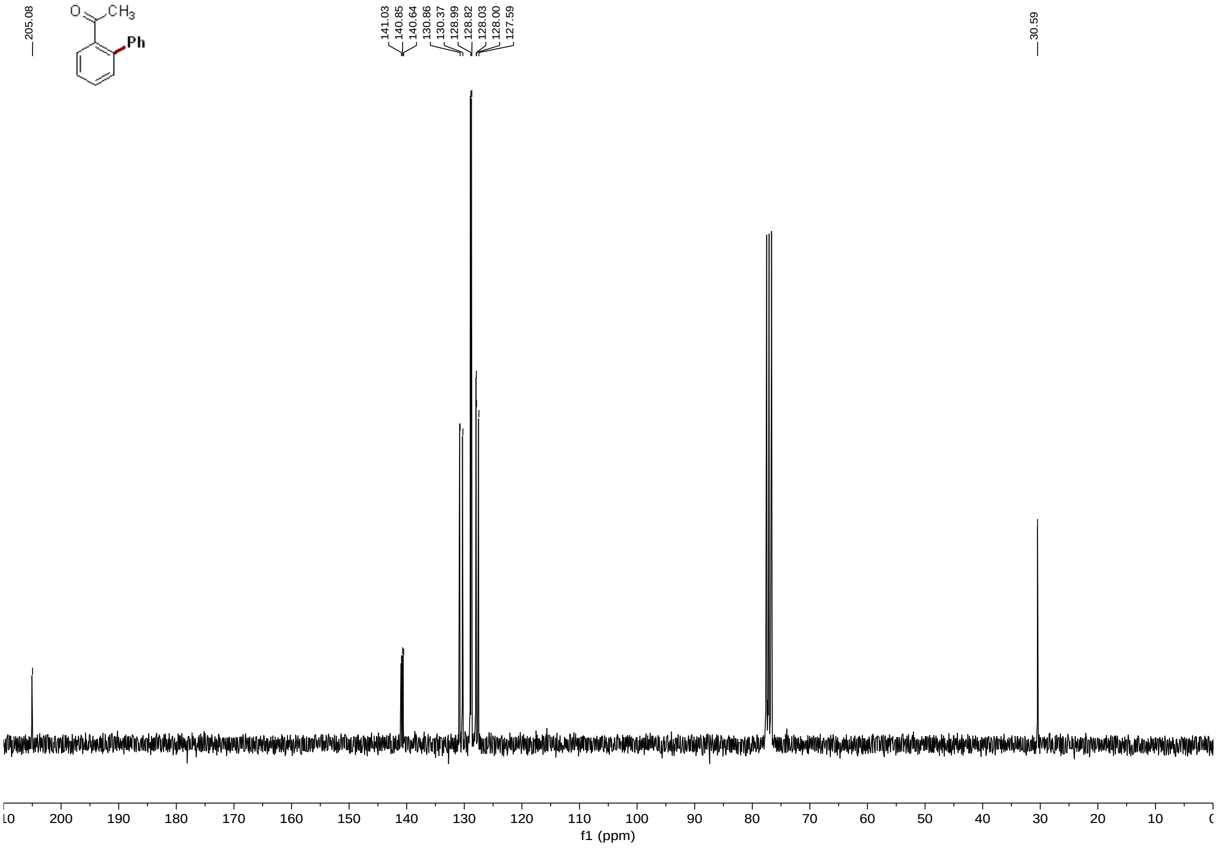
205.08
O
CH3
Ph
141.03
140.85
140.64
130.86
130.37
128.99
128.82
128.03
128.00
127.59
30.59
f1 (ppm)

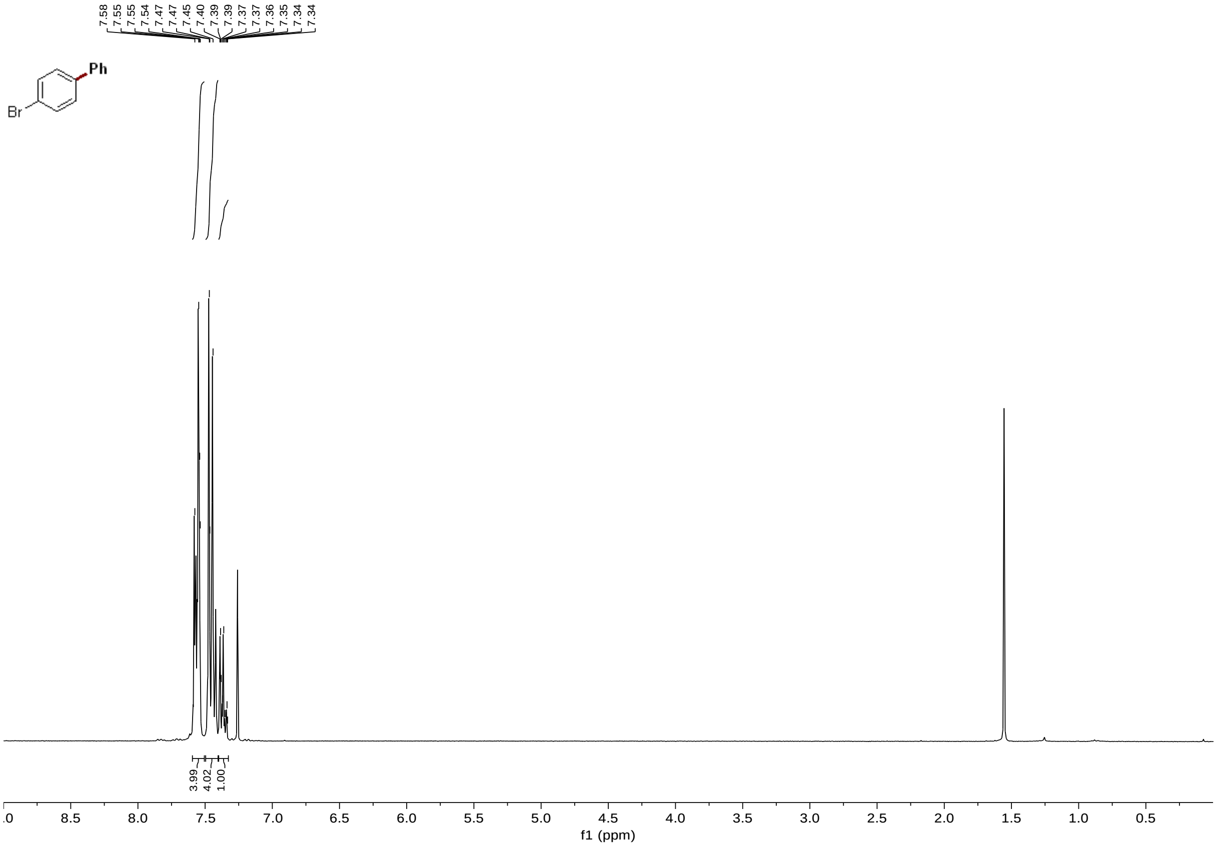
7.58
7.55
7.55
7.54
7.47
7.45
7.40
7.39
7.39
7.37
7.37
7.36
7.35
7.34
7.34
Ph
Br
3.99
4.02
1.00
f1 (ppm)

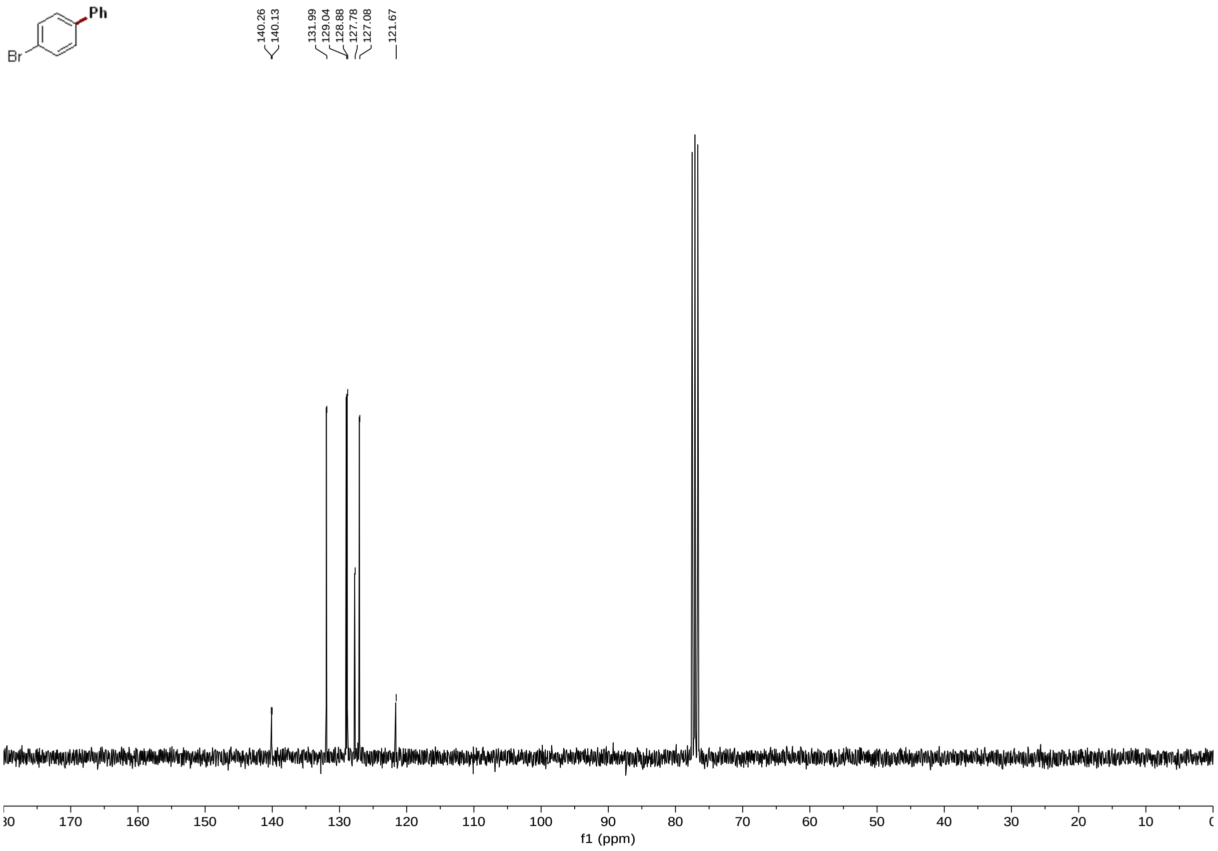
Ph
Br
140.26
140.13
131.99
129.04
128.88
127.78
127.08
121.67
f1 (ppm)

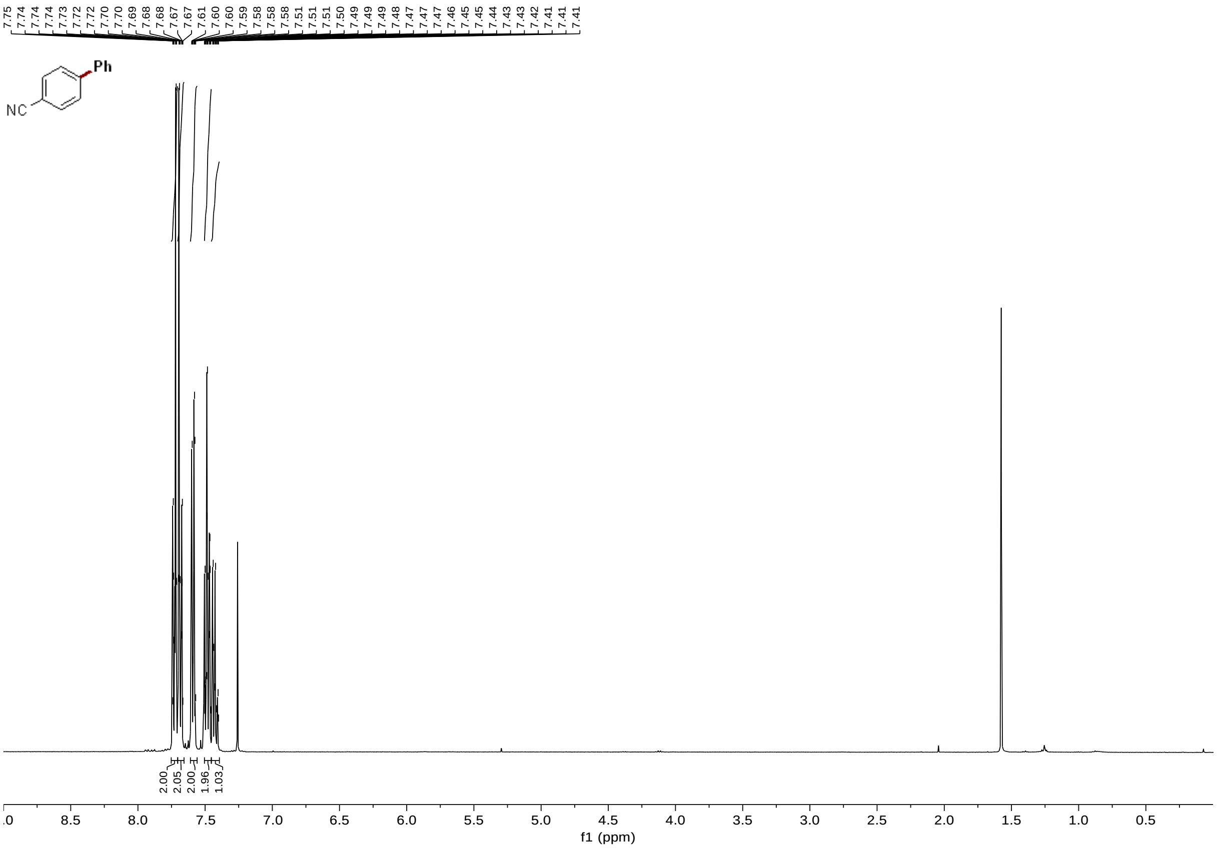
7.75 7.74 7.74 7.74 7.73 7.72 7.72 7.70 7.70 7.69 7.68 7.68 7.67 7.67 7.61 7.60 7.60 7.59 7.58 7.58 7.58 7.51 7.51 7.51 7.50 7.49 7.49 7.49 7.48 7.47 7.47 7.47 7.46 7.45 7.45 7.44 7.43 7.43 7.42 7.41 7.41 7.41
Ph
NC
2.00
2.05
2.00
1.96
1.03
f1 (ppm)

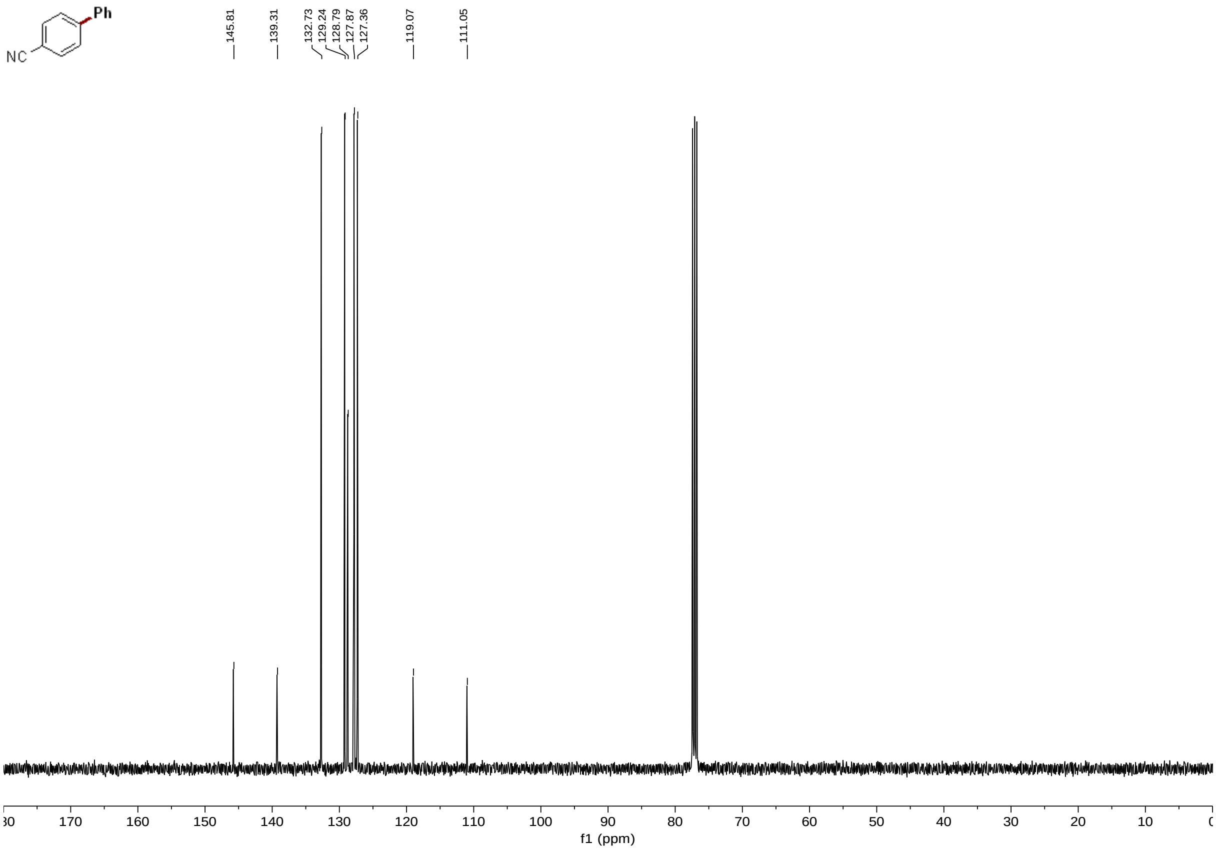
Ph
NC
145.81
139.31
132.73
129.24
128.79
127.87
127.36
119.07
111.05
f1 (ppm)

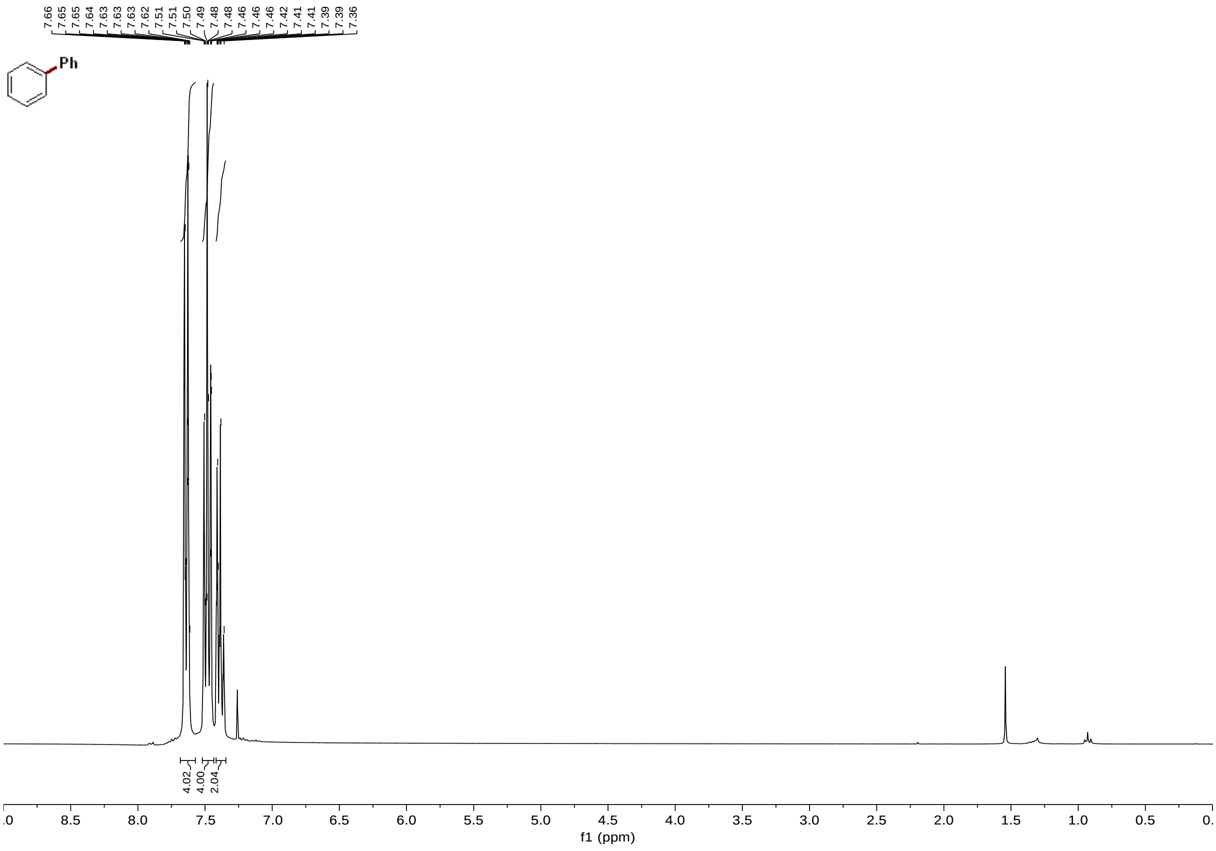
Ph
7.66
7.65
7.65
7.64
7.63
7.63
7.63
7.62
7.51
7.50
7.49
7.48
7.48
7.46
7.46
7.46
7.42
7.41
7.41
7.39
7.36
4.02
4.00
2.04
f1 (ppm)

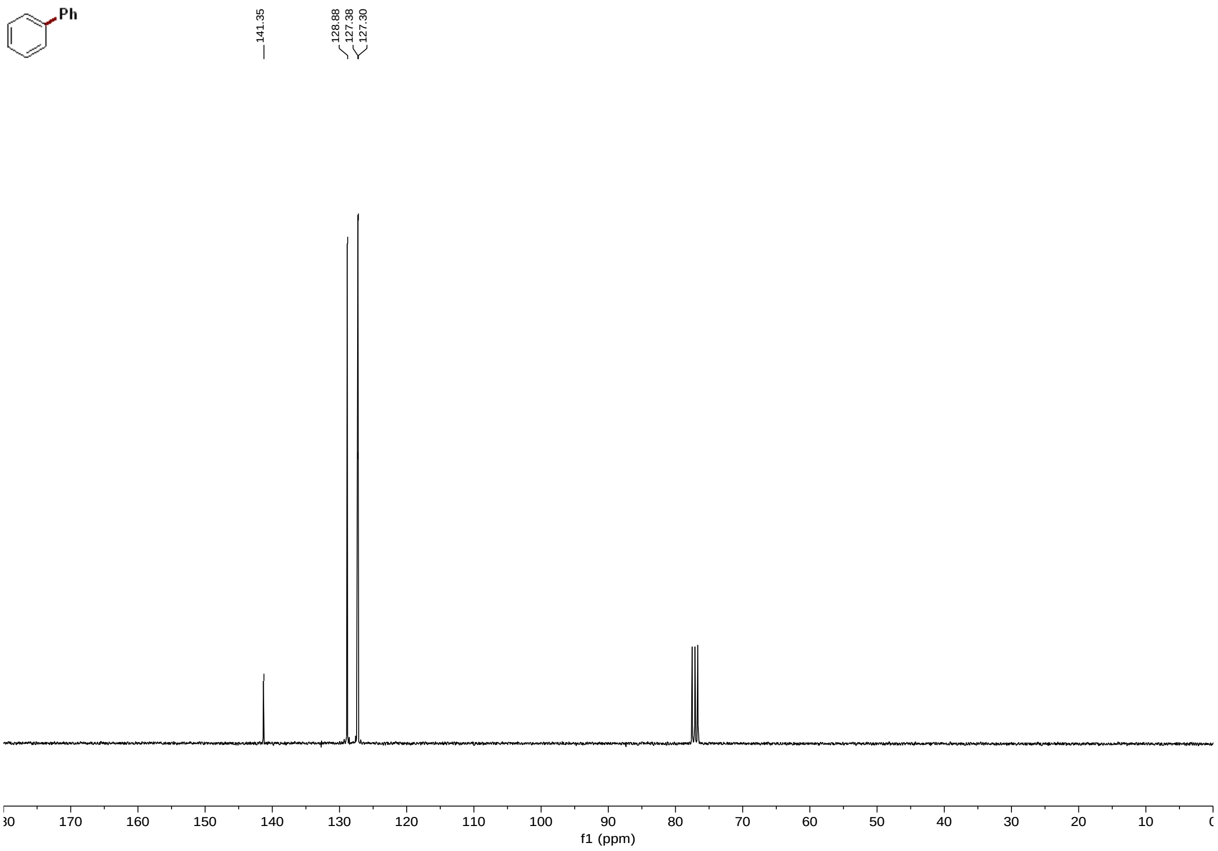
Ph
141.35
128.88
127.38
127.30
f1 (ppm)

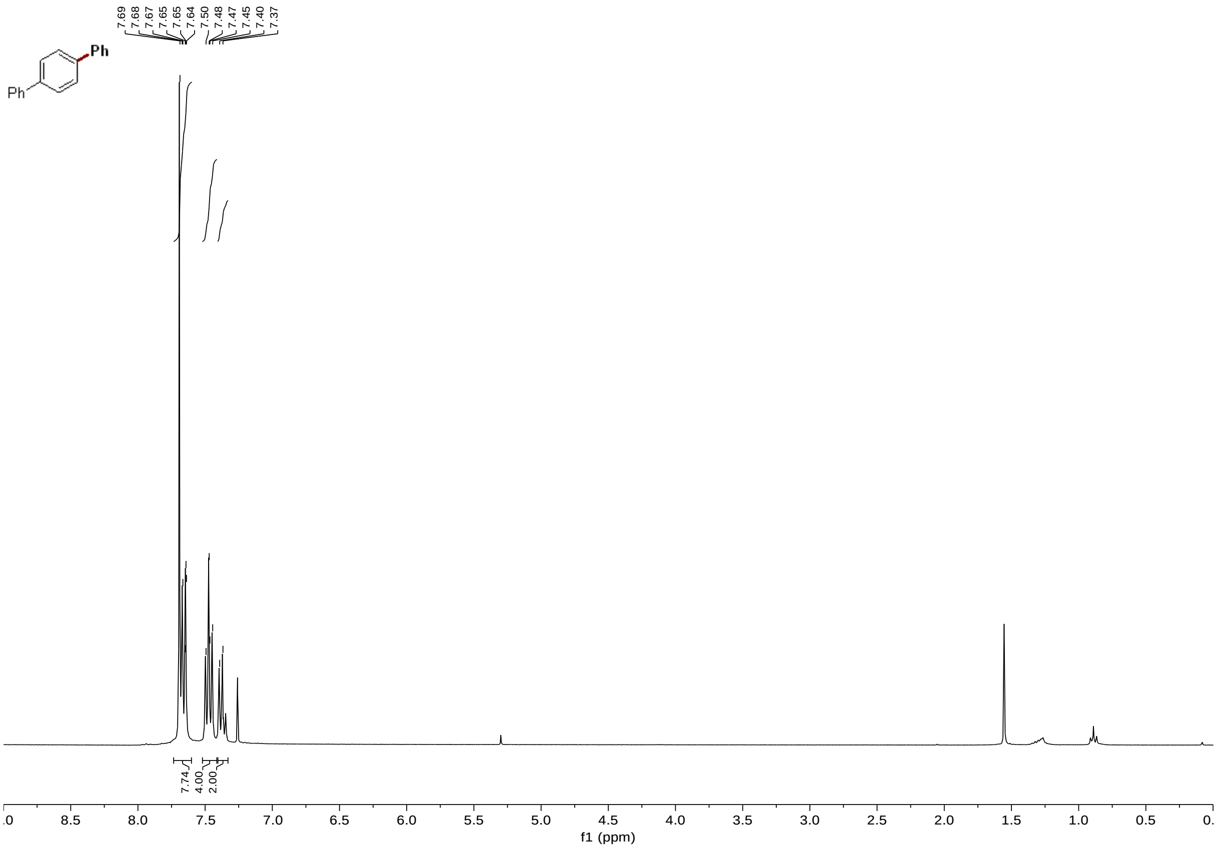
7.69
7.68
7.67
7.65
7.65
7.64
7.50
7.48
7.47
7.45
7.40
7.37
Ph
Ph
7.74
4.00
2.00
f1 (ppm)

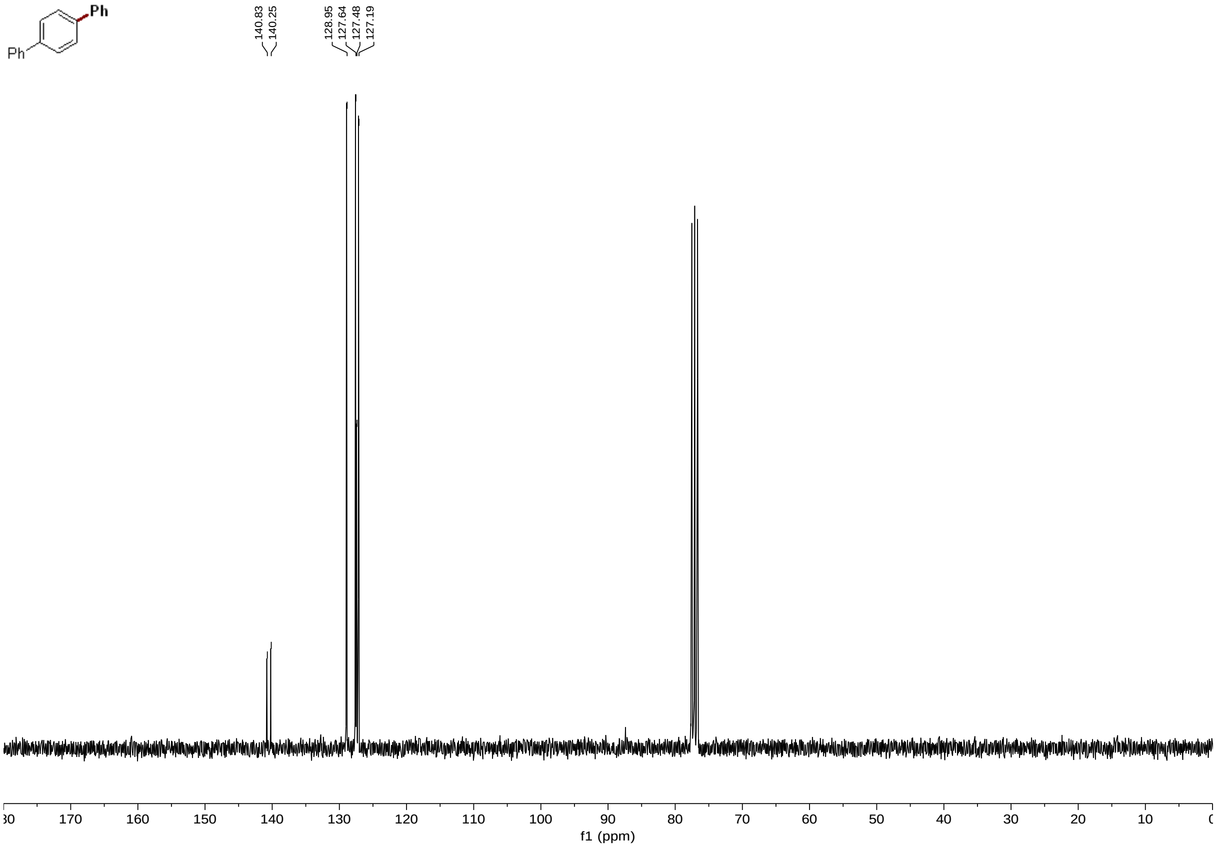
Ph
Ph
140.83
140.25
128.95
127.64
127.48
127.19
f1 (ppm)

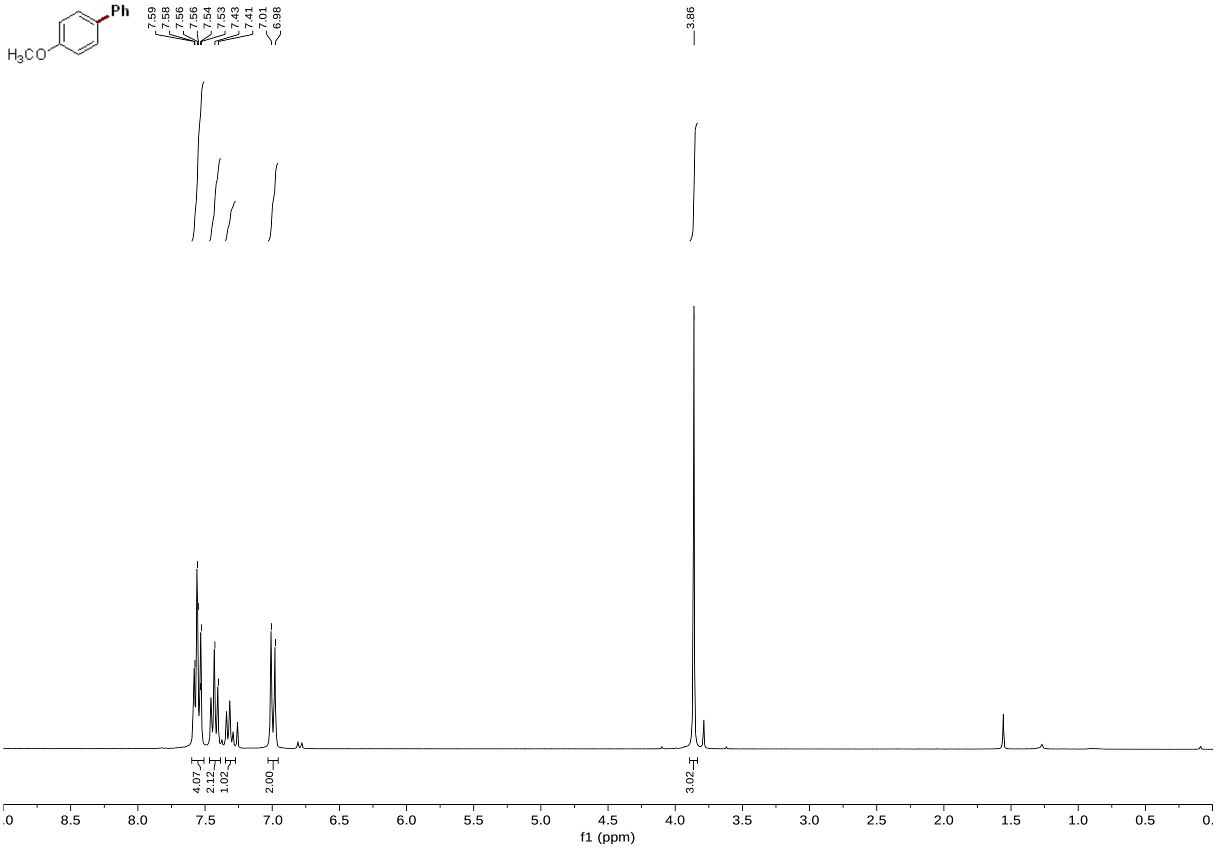
Ph
H3CO
7.59
7.58
7.56
7.56
7.54
7.53
7.43
7.41
7.01
6.98
3.86
4.07
2.12
1.02
2.00
3.02
f1 (ppm)

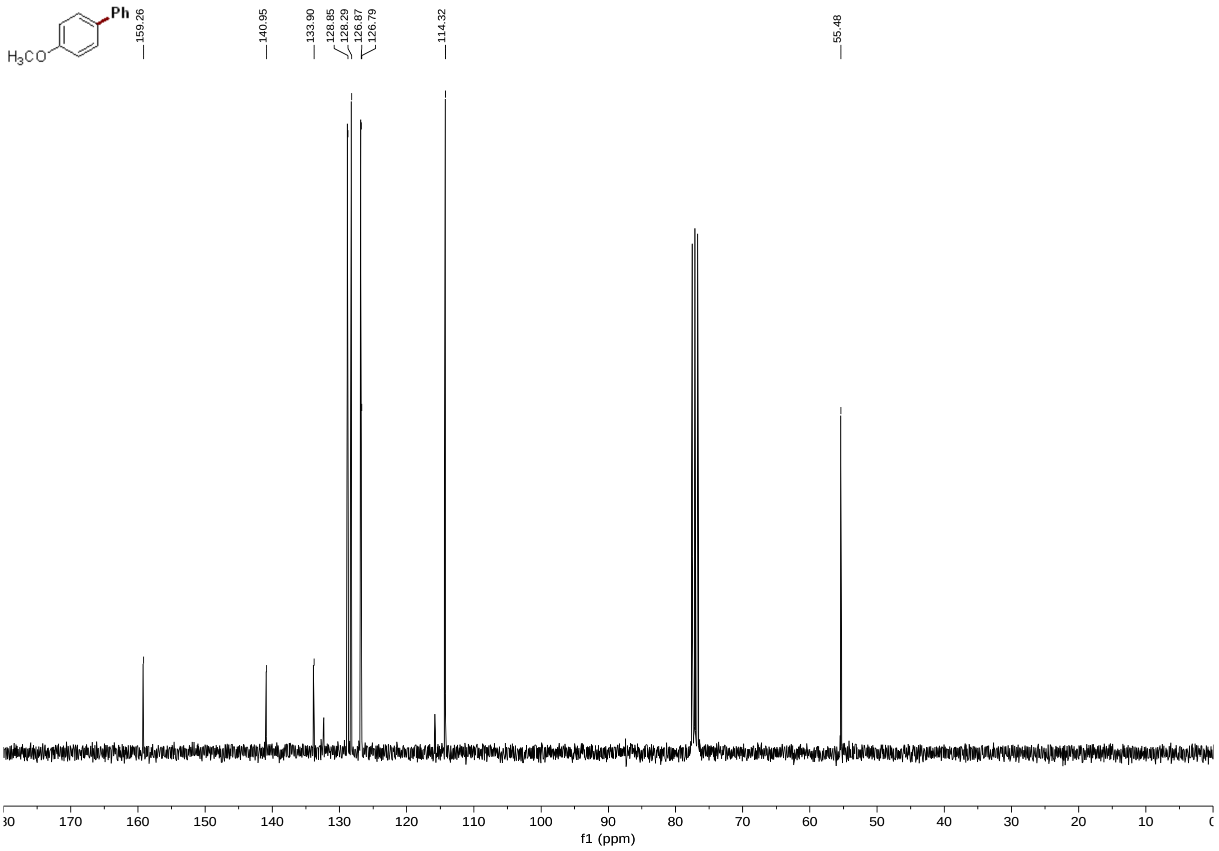
Ph
H3CO
159.26
140.95
133.90
128.85
128.29
126.87
126.79
114.32
55.48
f1 (ppm)

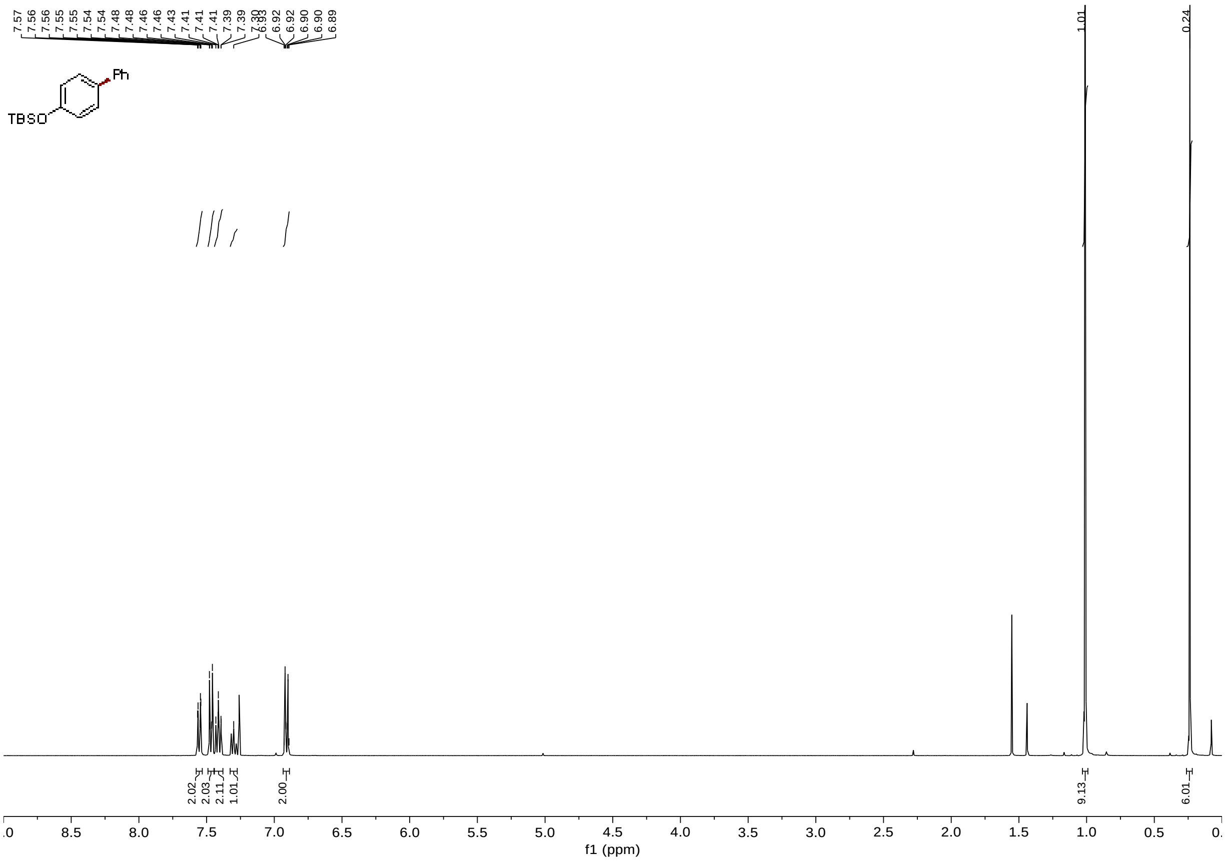
Ph
TBSO
7.57
7.56
7.56
7.55
7.55
7.54
7.54
7.48
7.48
7.46
7.46
7.43
7.41
7.41
7.41
7.39
7.39
6.92
6.92
6.90
6.90
6.89
1.01
0.24
2.02
2.03
2.11
1.01
2.00
9.13
6.01
f1 (ppm)

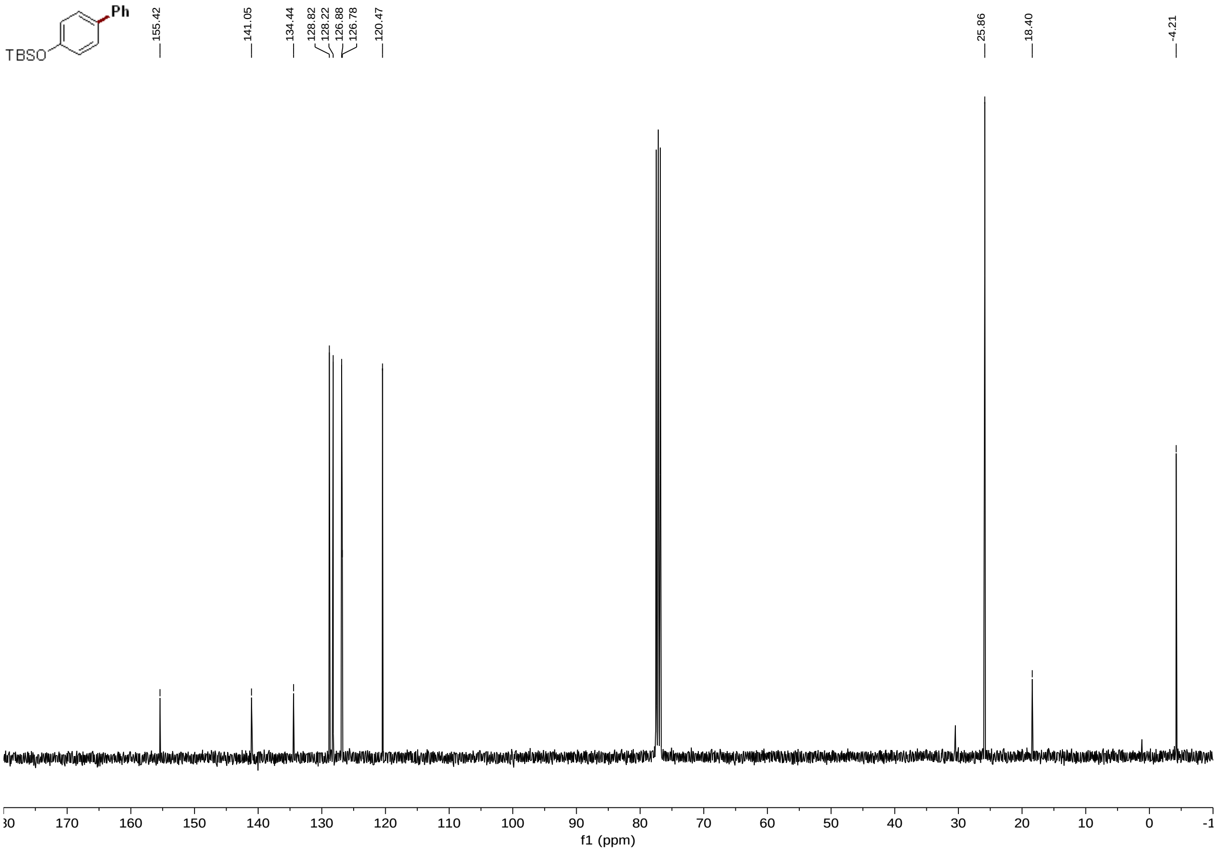
TBSO
Ph
155.42
141.05
134.44
128.82
128.22
126.88
126.78
120.47
25.86
18.40
-4.21
f1 (ppm)

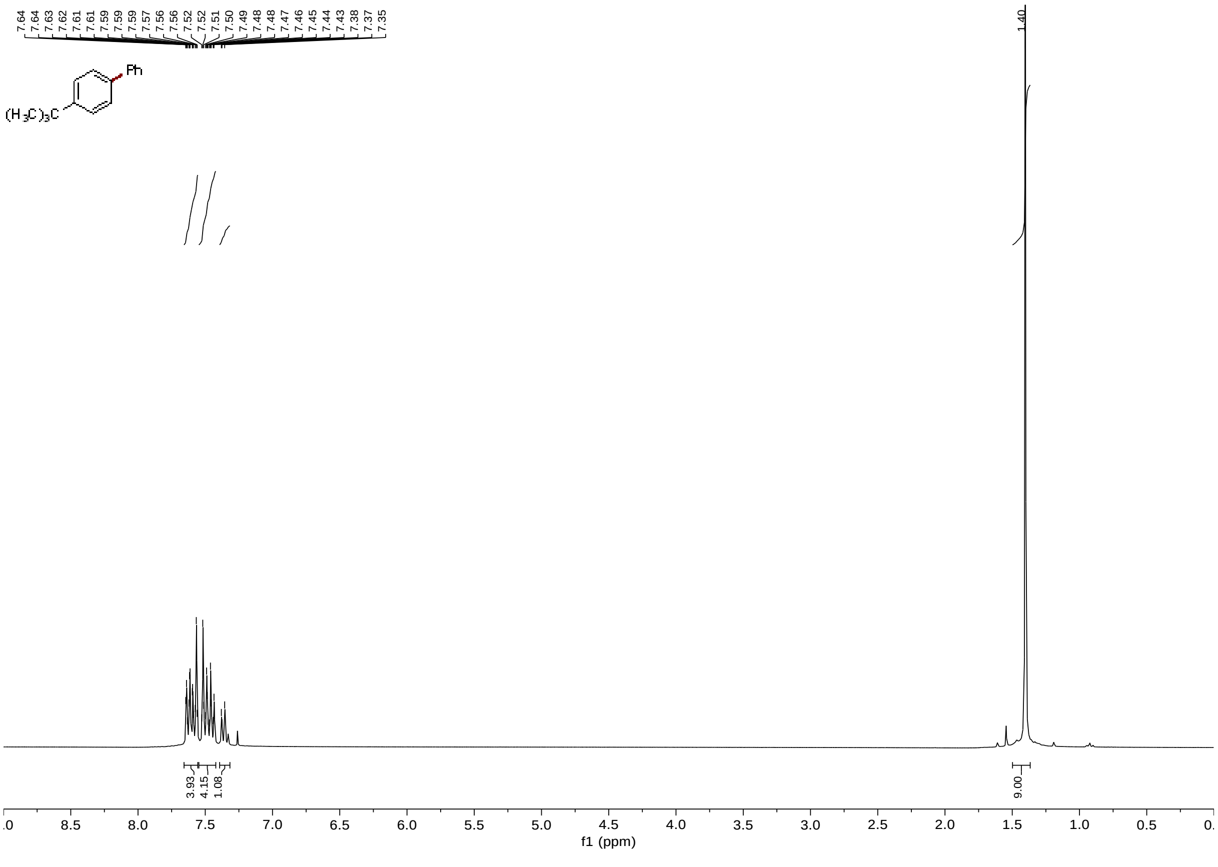
(H3C)3C
Ph
7.64 7.64 7.63 7.62 7.61 7.61 7.59 7.59 7.59 7.57 7.56 7.56 7.52 7.52 7.51 7.50 7.49 7.48 7.48 7.47 7.46 7.45 7.44 7.43 7.38 7.37 7.35
1.40
3.93
4.15
1.08
9.00
f1 (ppm)

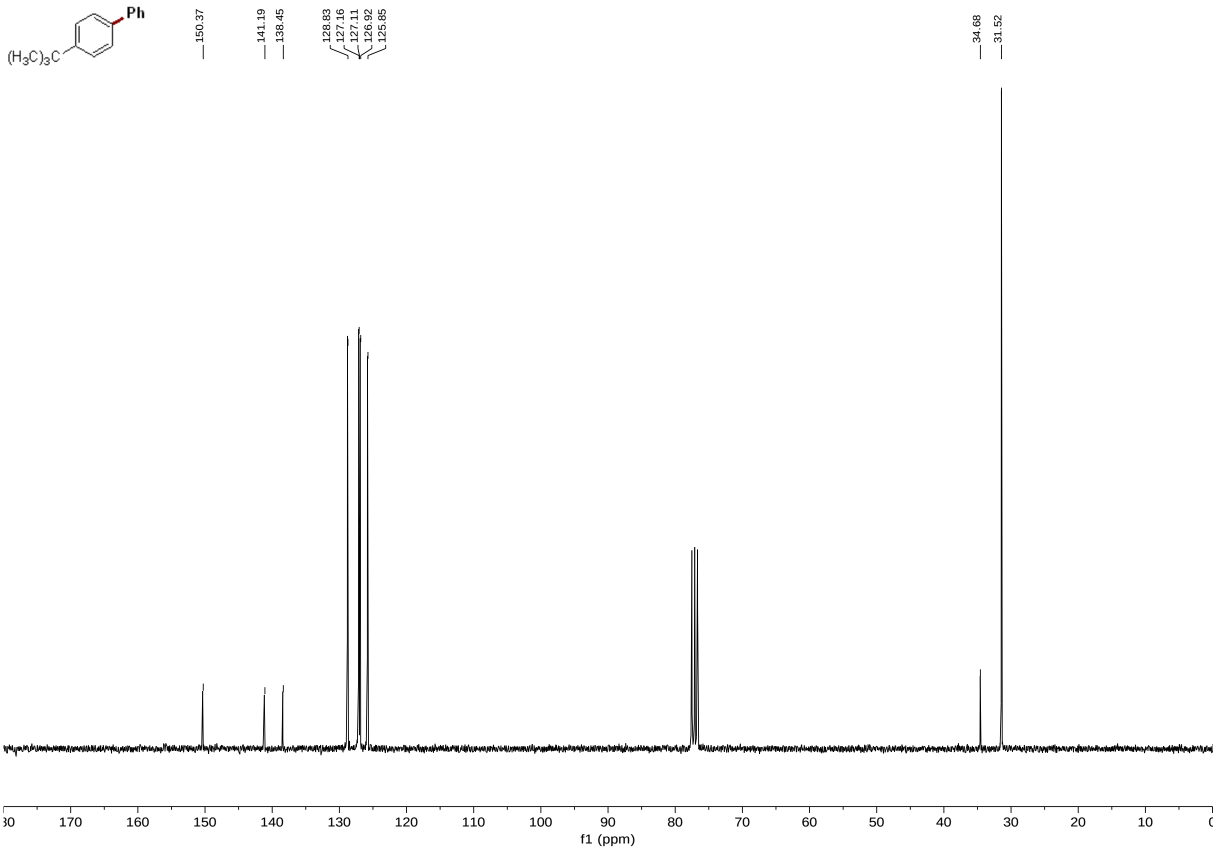
Ph
(H3C)3C
150.37
141.19
138.45
128.83
127.16
127.11
126.92
125.85
34.68
31.52
f1 (ppm)

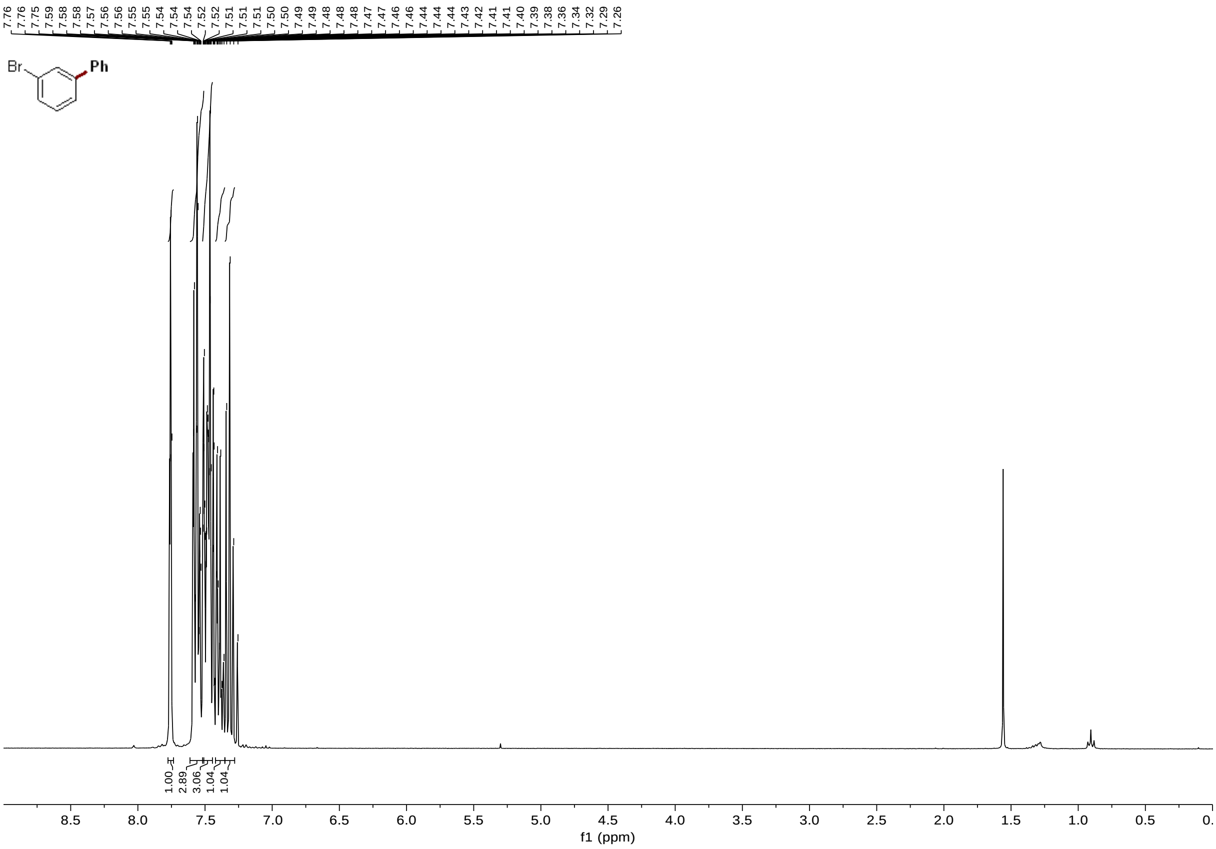
7.76 7.76 7.75 7.59 7.58 7.58 7.57 7.56 7.56 7.55 7.55 7.54 7.54 7.54 7.52 7.52 7.51 7.51 7.51 7.50 7.50 7.49 7.49 7.48 7.48 7.48 7.47 7.46 7.46 7.44 7.44 7.44 7.43 7.42 7.41 7.41 7.40 7.39 7.38 7.36 7.34 7.32 7.29 7.26
Br
Ph
1.00
2.89
3.06
1.04
1.04
f1 (ppm)

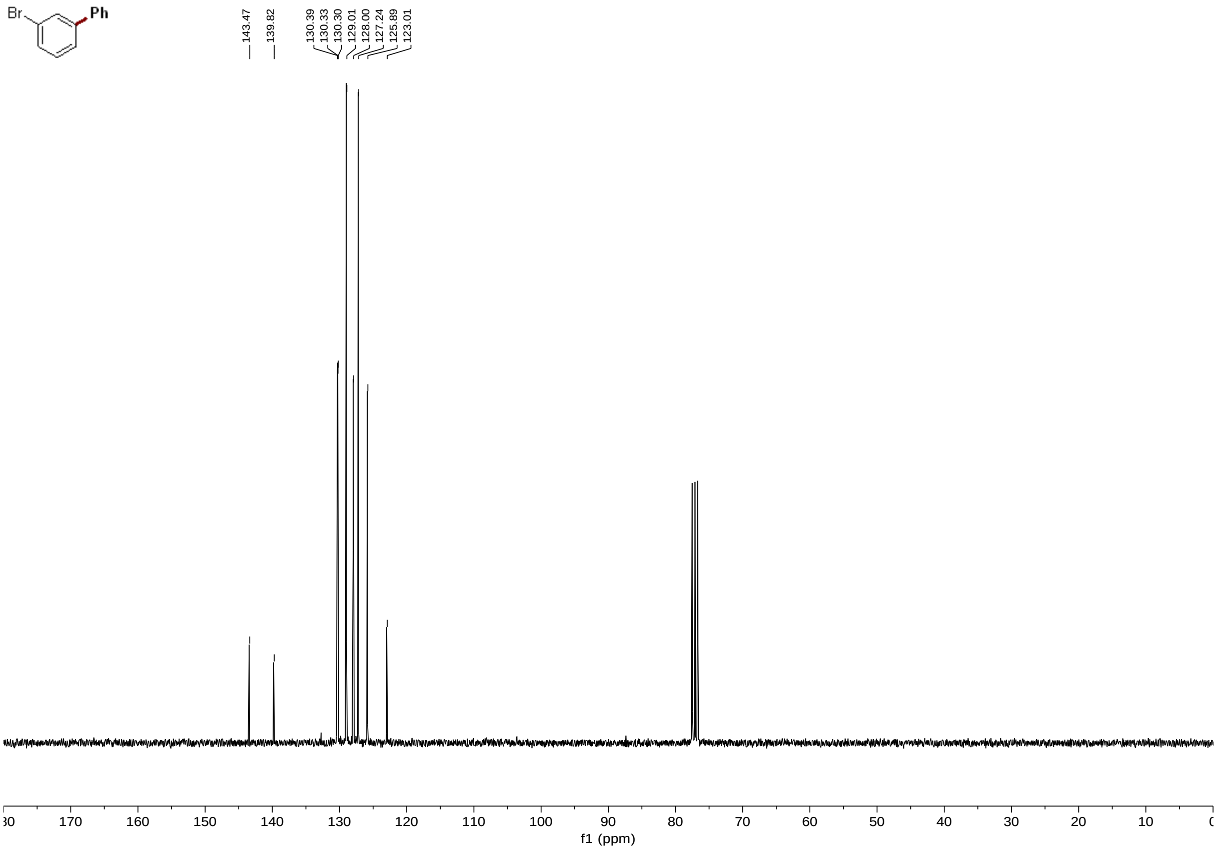

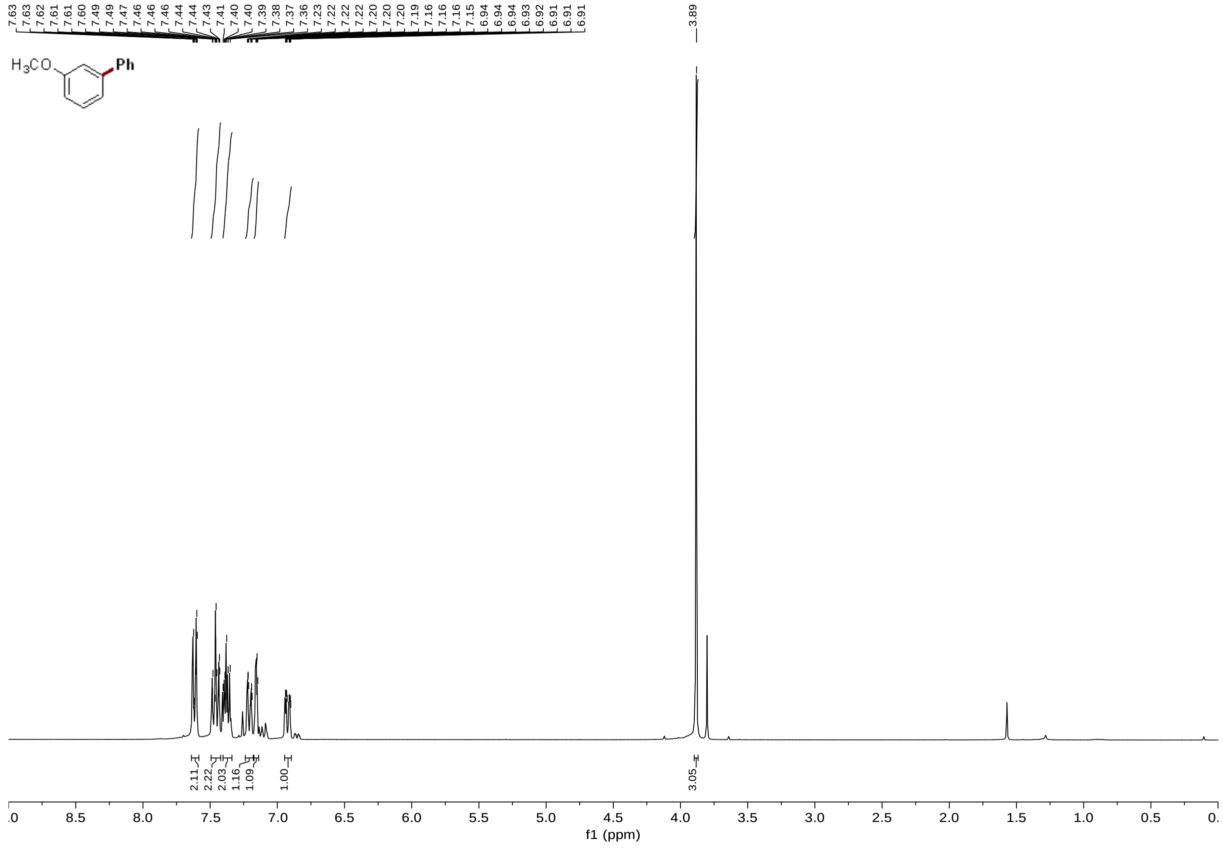

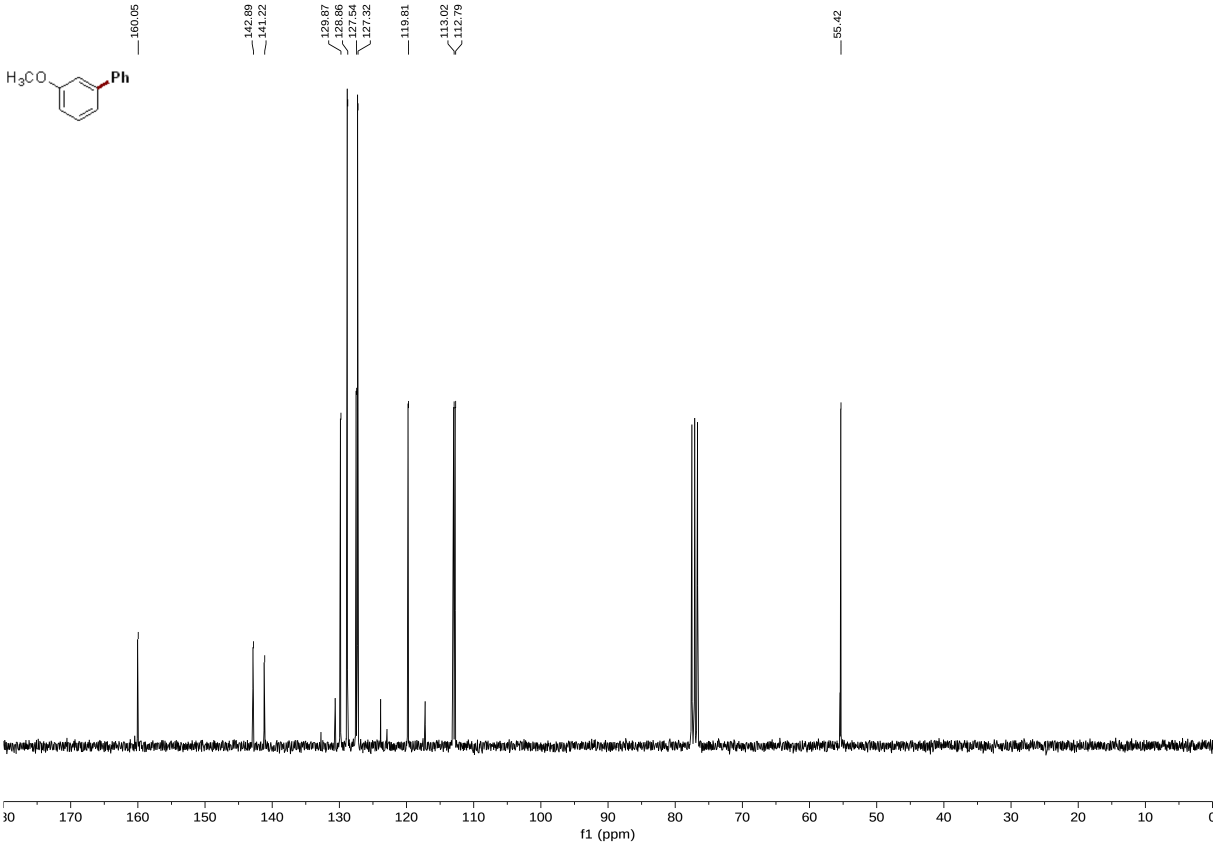
160.05
142.89
141.22
129.87
128.86
127.54
127.32
119.81
113.02
112.79
55.42
H3CO
Ph
170 160 150 140 130 120 110 100 90 80 70 60 50 40 30 20 10
f1 (ppm)

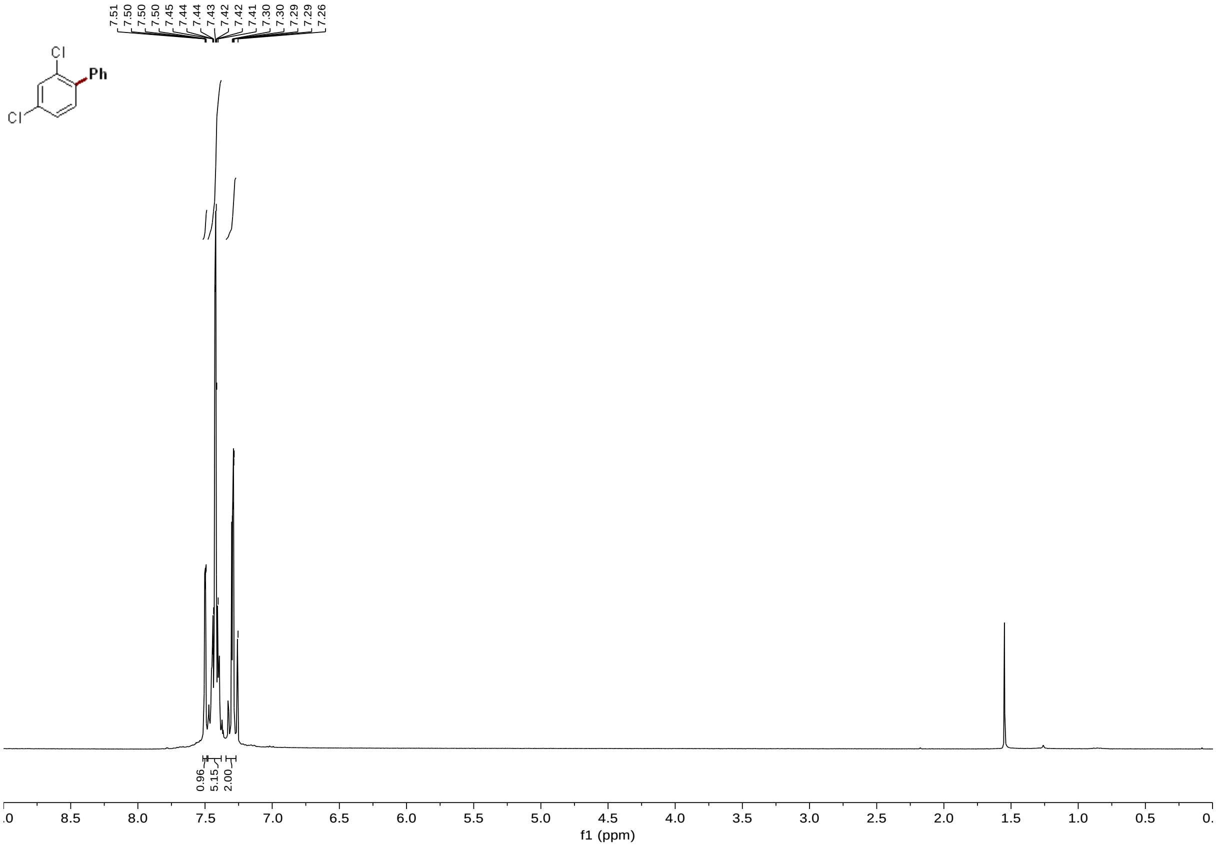
7.51
7.50
7.50
7.50
7.45
7.44
7.44
7.43
7.42
7.42
7.41
7.30
7.30
7.29
7.29
7.26
Cl
Ph
Cl
0.96
5.15
2.00
8.5 8.0 7.5 7.0 6.5 6.0 5.5 5.0 4.5 4.0 3.5 3.0 2.5 2.0 1.5 1.0 0.5
f1 (ppm)

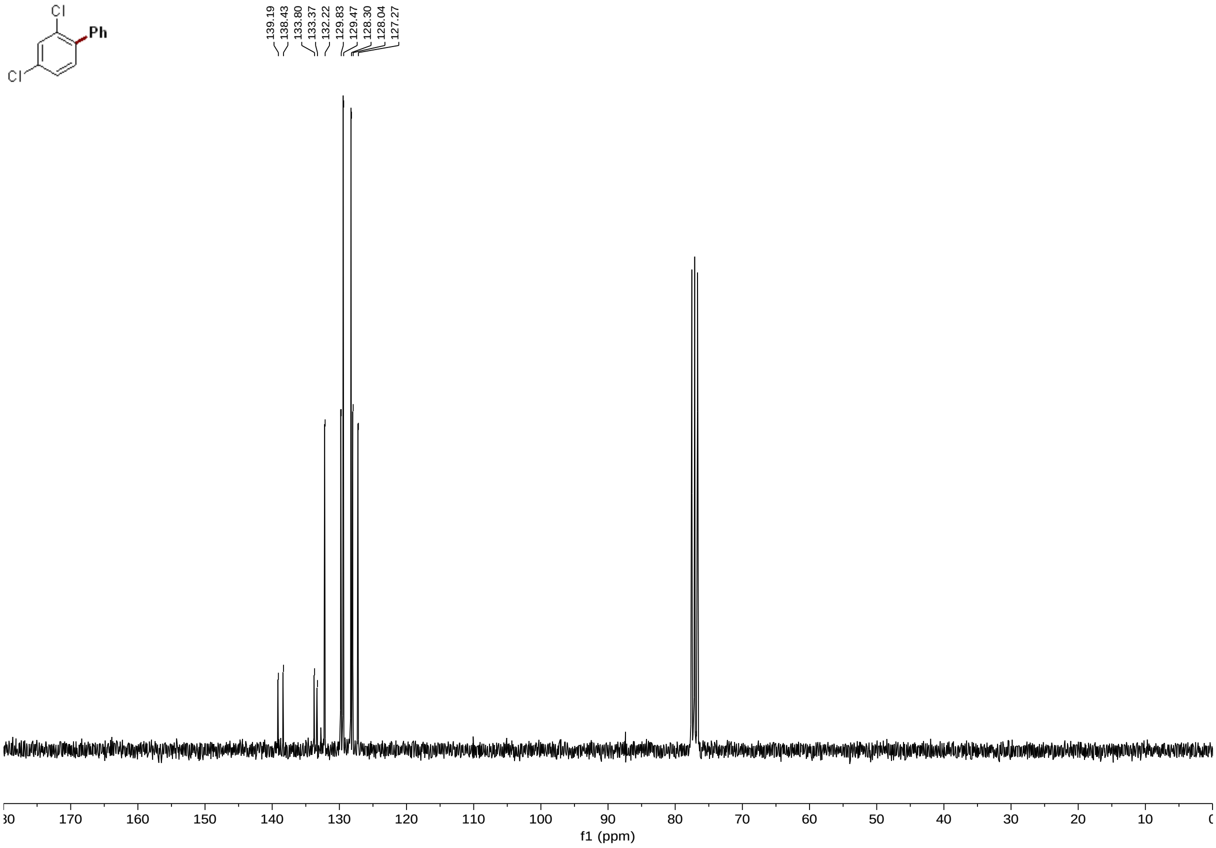
Cl
Ph
Cl
139.19
138.43
133.80
133.37
132.22
129.83
129.47
128.30
128.04
127.27
f1 (ppm)

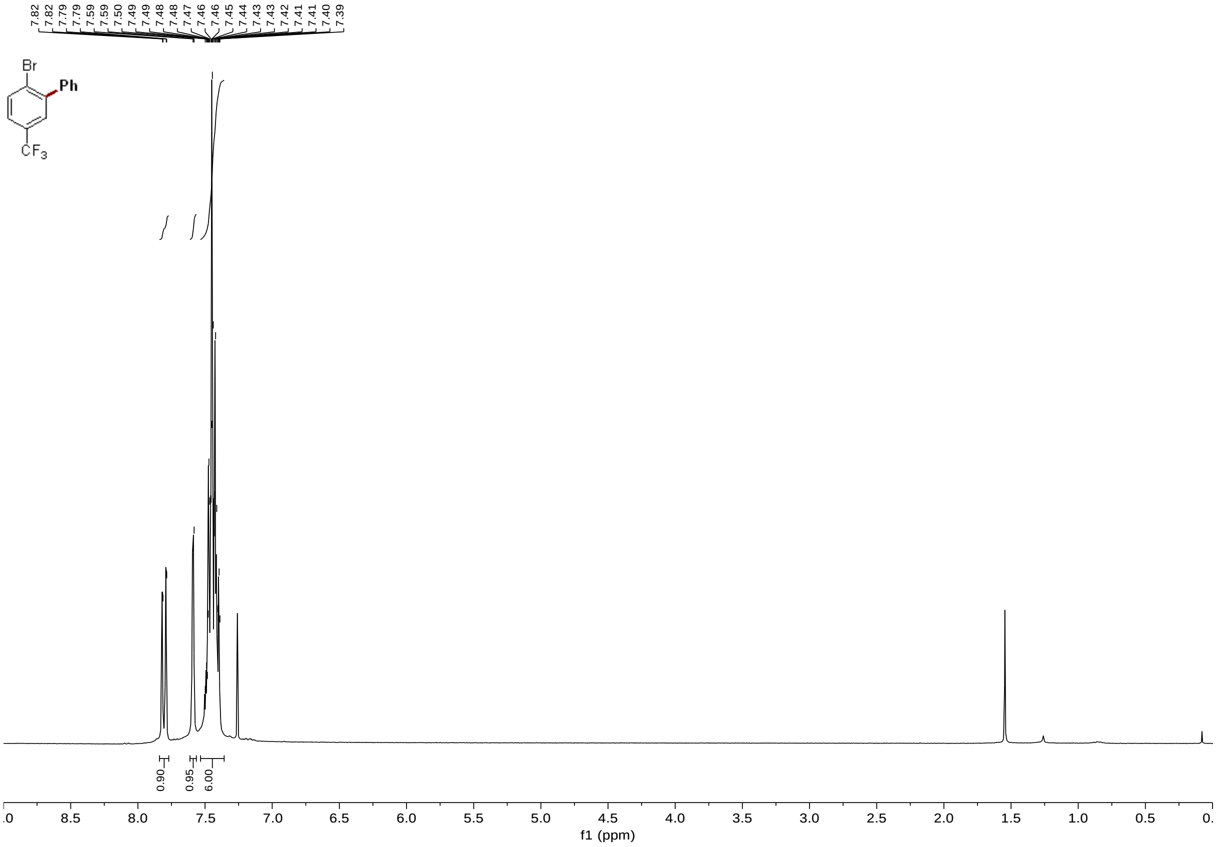
Br
Ph
CF3
7.82
7.82
7.79
7.79
7.59
7.59
7.50
7.49
7.49
7.48
7.48
7.47
7.46
7.46
7.45
7.44
7.43
7.43
7.42
7.41
7.41
7.40
7.39
0.90
0.95
6.00
f1 (ppm)

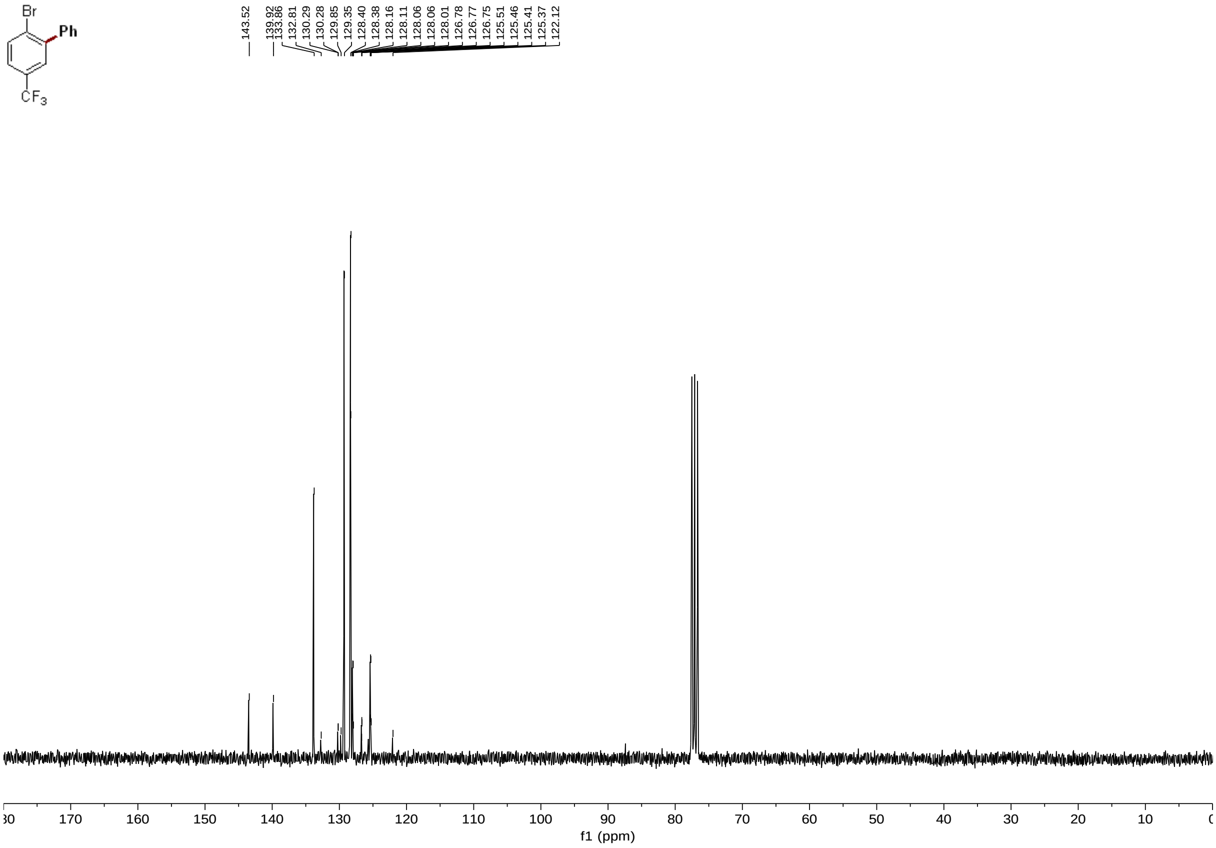
Br
Ph
CF3
143.52
139.92
133.86
132.81
130.29
130.28
129.85
129.35
128.40
128.38
128.16
128.11
128.06
128.06
128.01
126.78
126.77
126.75
125.51
125.46
125.41
125.37
122.12
f1 (ppm)

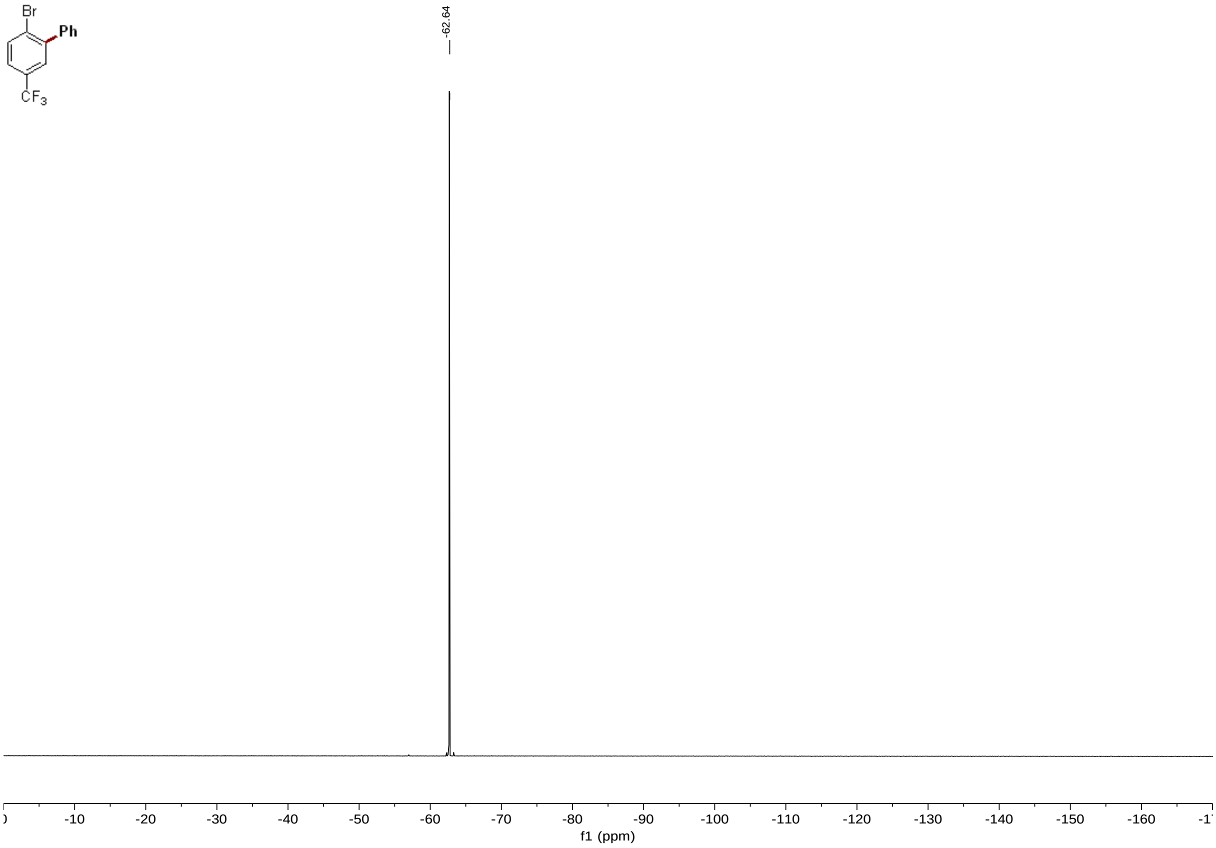
Br
Ph
CF3
-62.64
f1 (ppm)

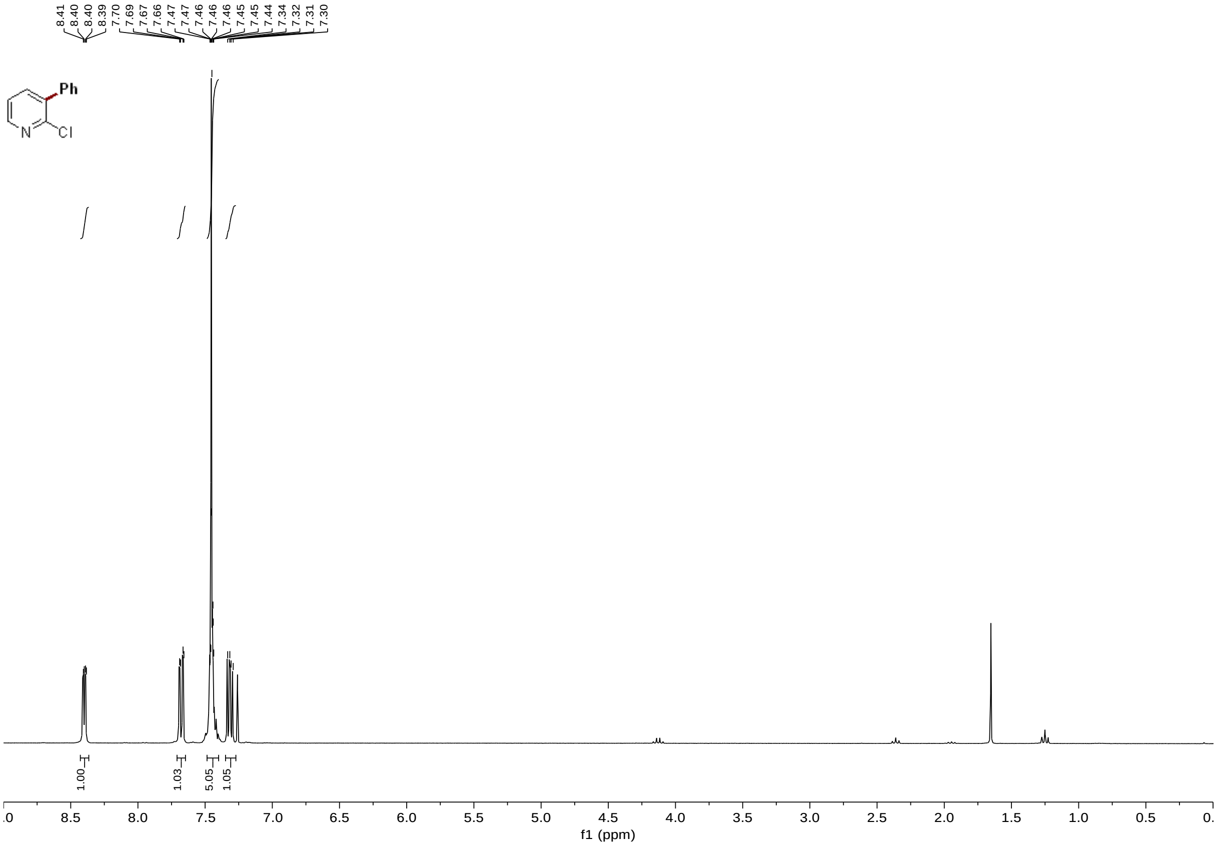
8.41
8.40
8.40
8.39
7.70
7.69
7.67
7.66
7.47
7.47
7.46
7.46
7.46
7.45
7.45
7.44
7.34
7.32
7.31
7.30
Ph
N
Cl
1.00
1.03
5.05
1.05
f1 (ppm)

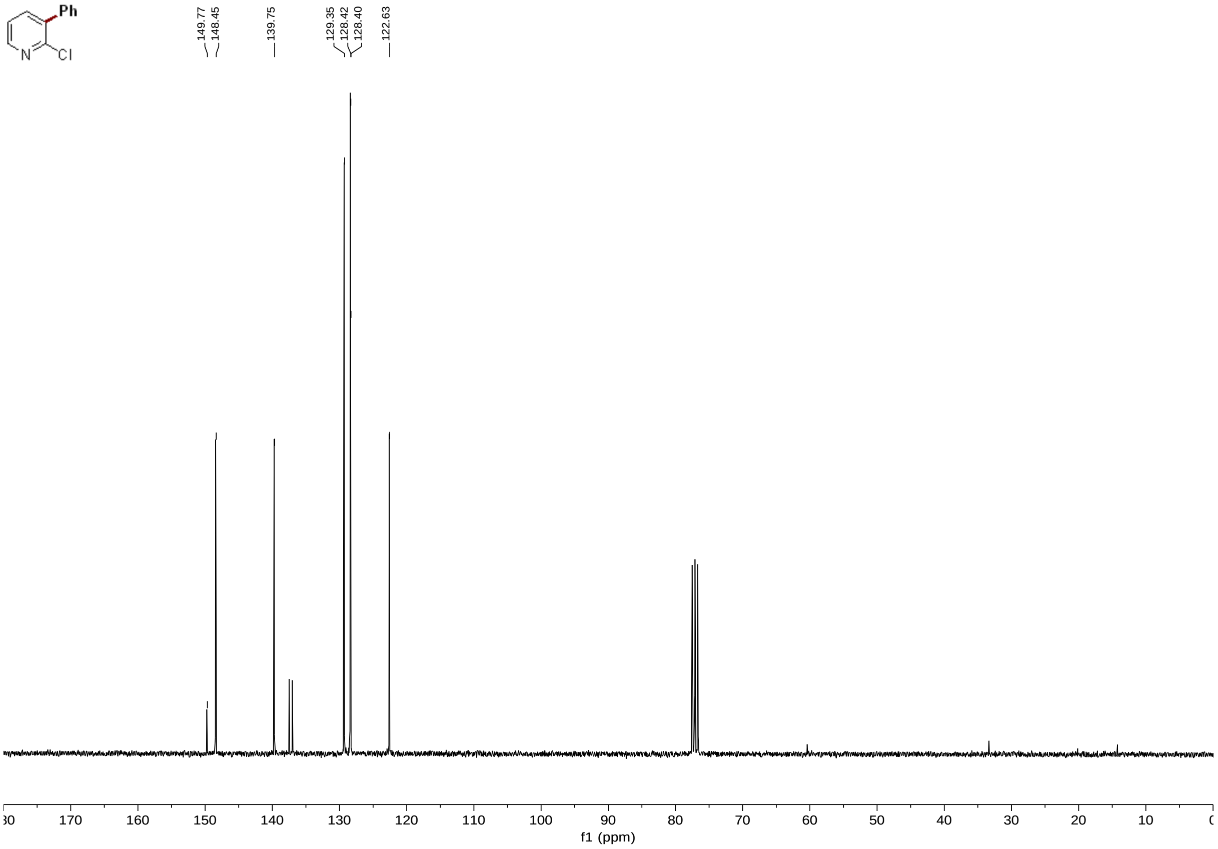
Ph
N
Cl
149.77
148.45
139.75
129.35
128.42
128.40
122.63
f1 (ppm)

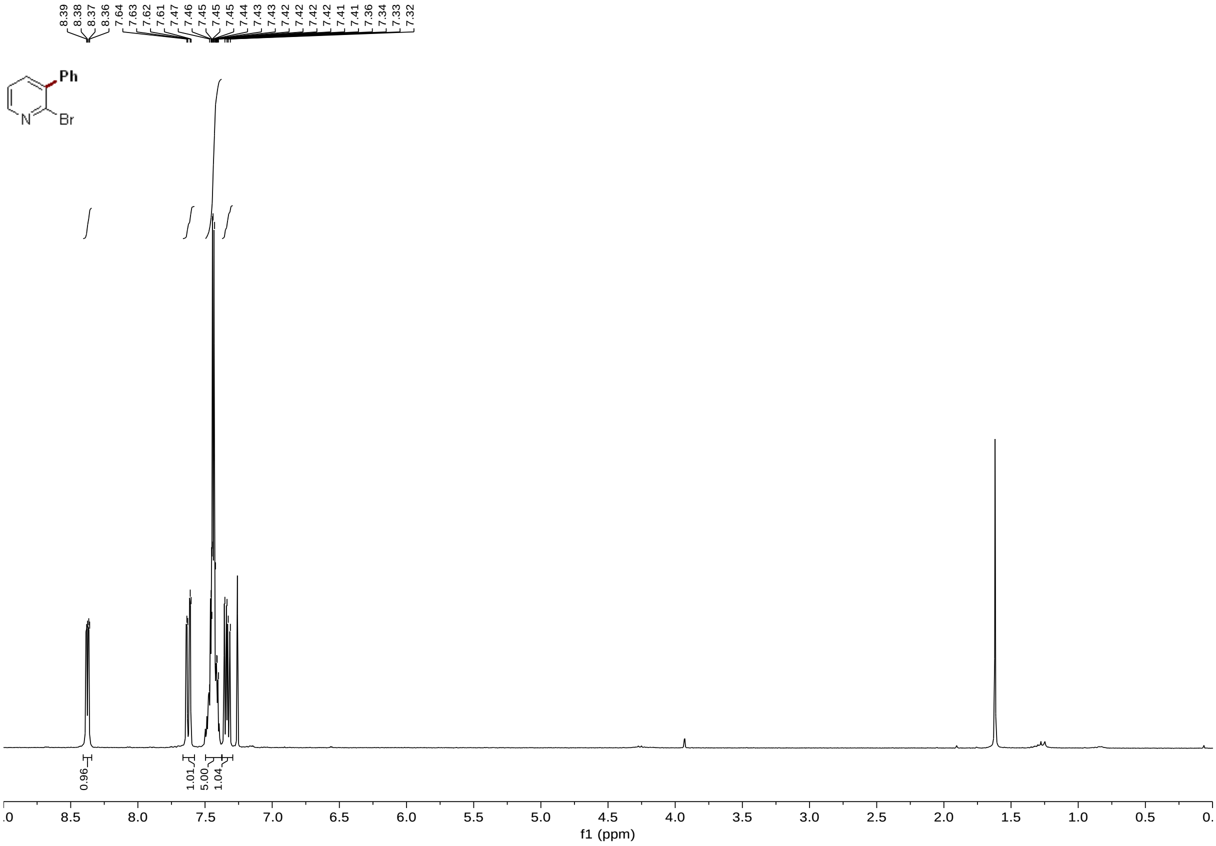
8.39
8.38
8.37
8.36
7.64
7.63
7.62
7.61
7.47
7.46
7.45
7.45
7.45
7.44
7.43
7.43
7.42
7.42
7.42
7.42
7.41
7.41
7.36
7.34
7.33
7.32
Ph
N
Br
0.96
1.01
5.00
1.04
8.5
8.0
7.5
7.0
6.5
6.0
5.5
5.0
4.5
4.0
3.5
3.0
2.5
2.0
1.5
1.0
0.5
f1 (ppm)

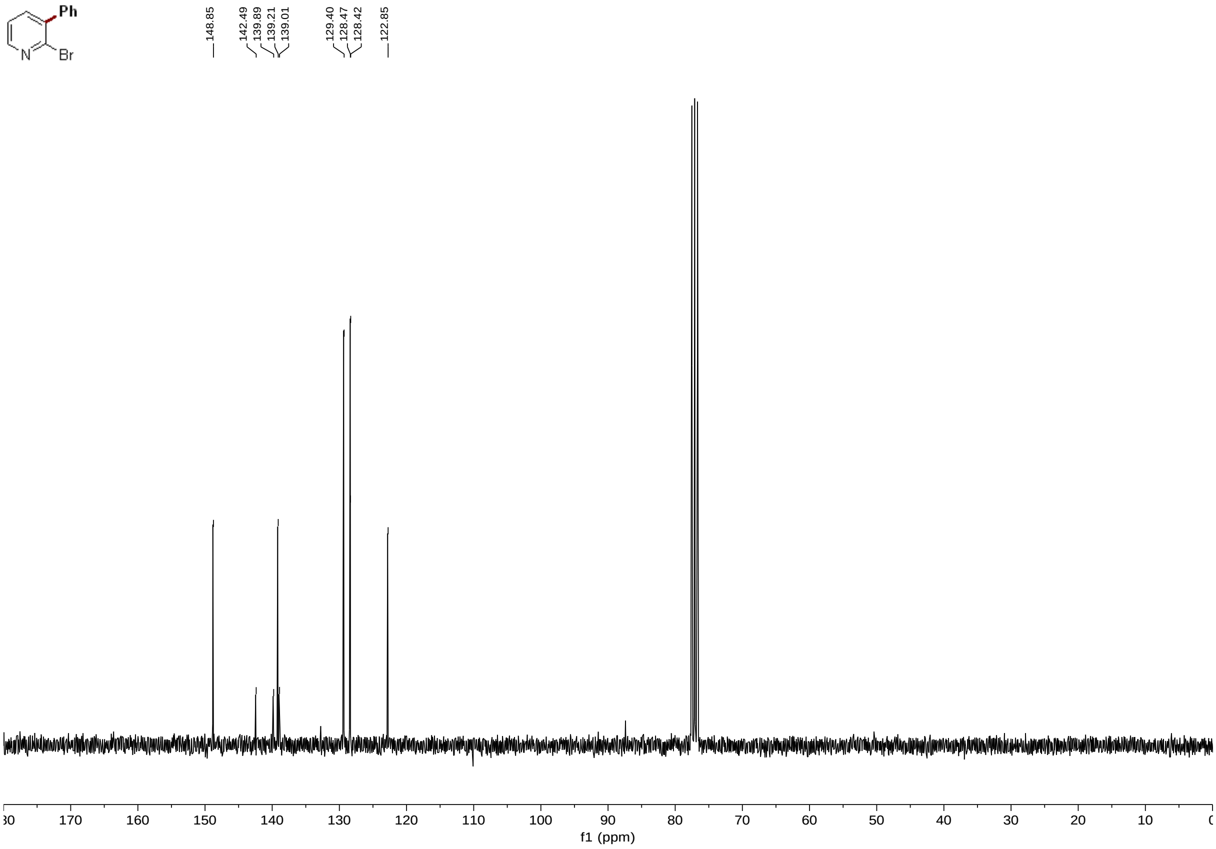
Ph
N
Br
148.85
142.49
139.89
139.21
139.01
129.40
128.47
128.42
122.85
170
160
150
140
130
120
110
100
90
80
70
60
50
40
30
20
10
f1 (ppm)

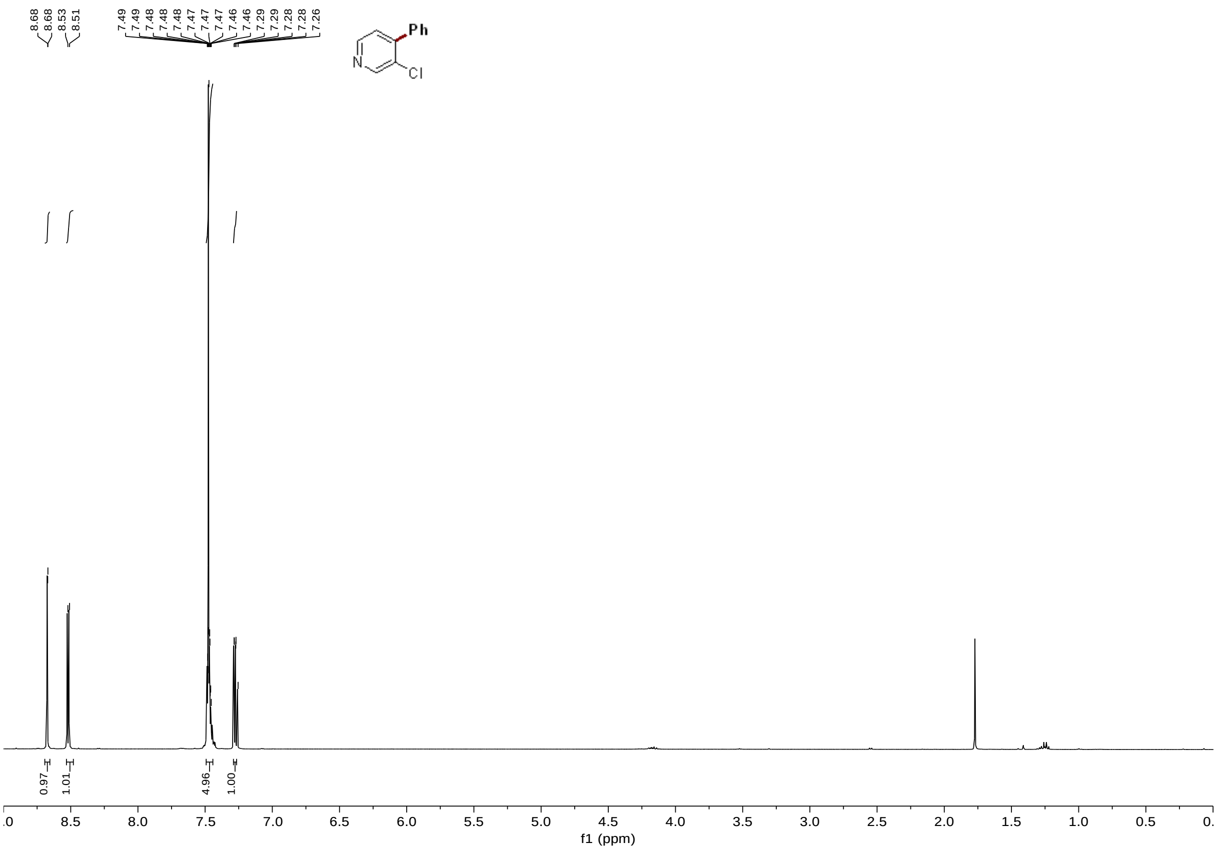

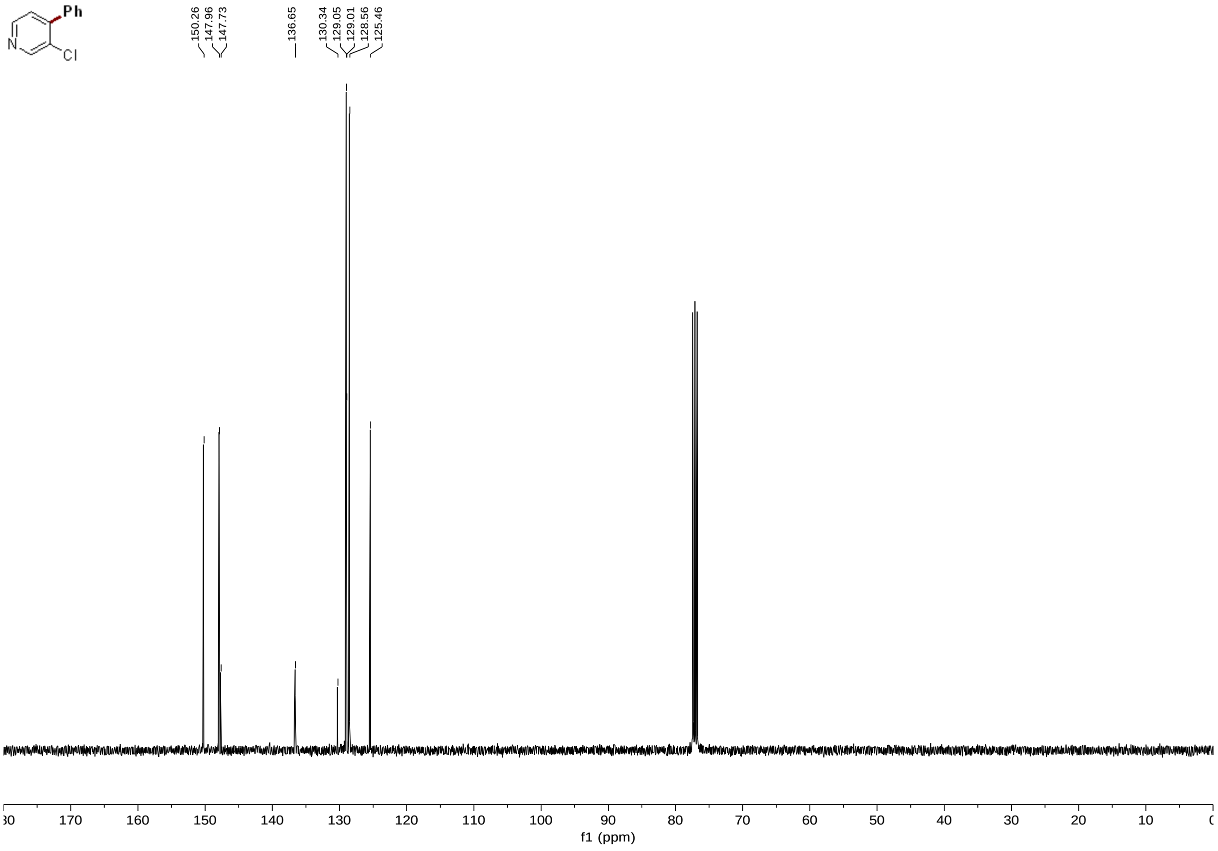

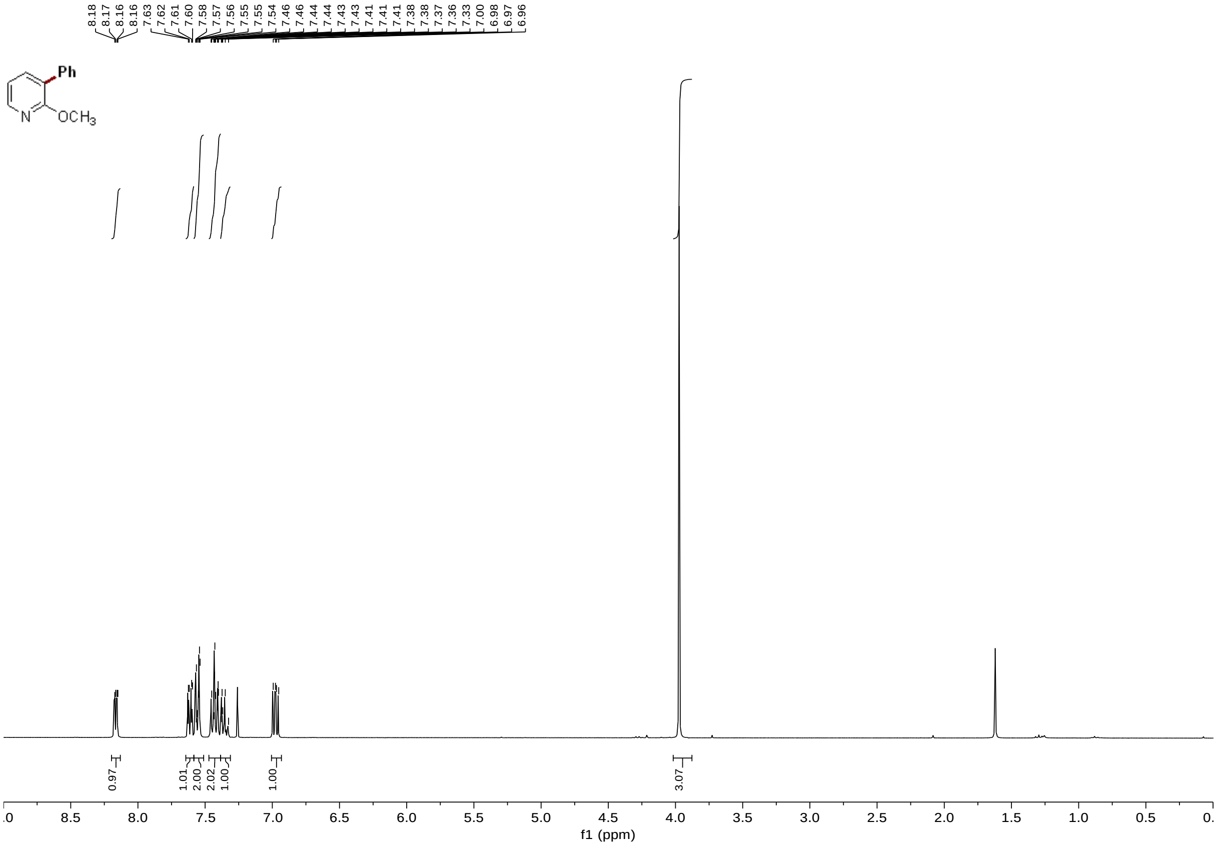
Ph
N
OCH3
8.18
8.17
8.16
8.16
7.63
7.62
7.61
7.60
7.58
7.57
7.56
7.55
7.55
7.54
7.46
7.46
7.44
7.44
7.43
7.43
7.41
7.41
7.38
7.38
7.37
7.36
7.33
7.00
6.98
6.97
6.96
0.97
1.01
2.00
2.02
1.00
1.00
3.07
f1 (ppm)

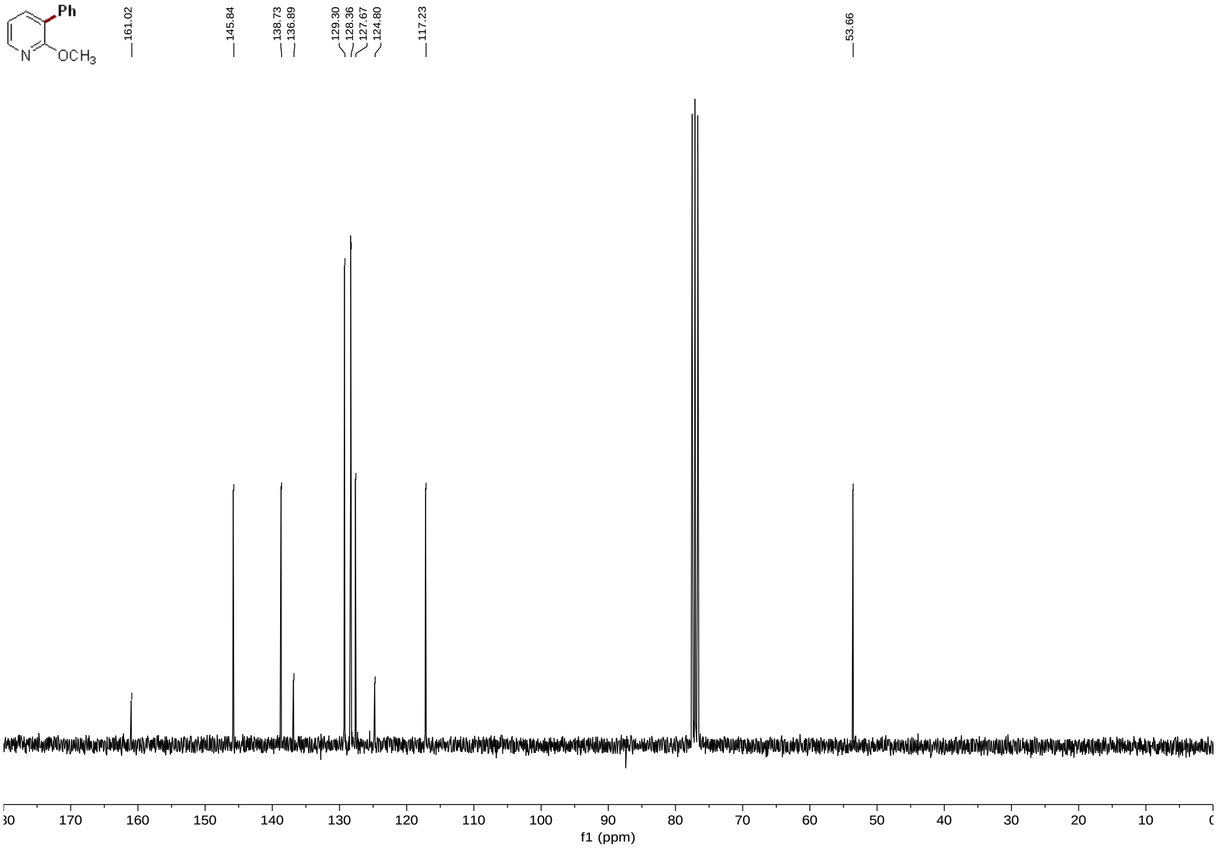
Ph
N
OCH3
161.02
145.84
138.73
136.89
129.30
128.36
127.67
124.80
117.23
53.66
f1 (ppm)

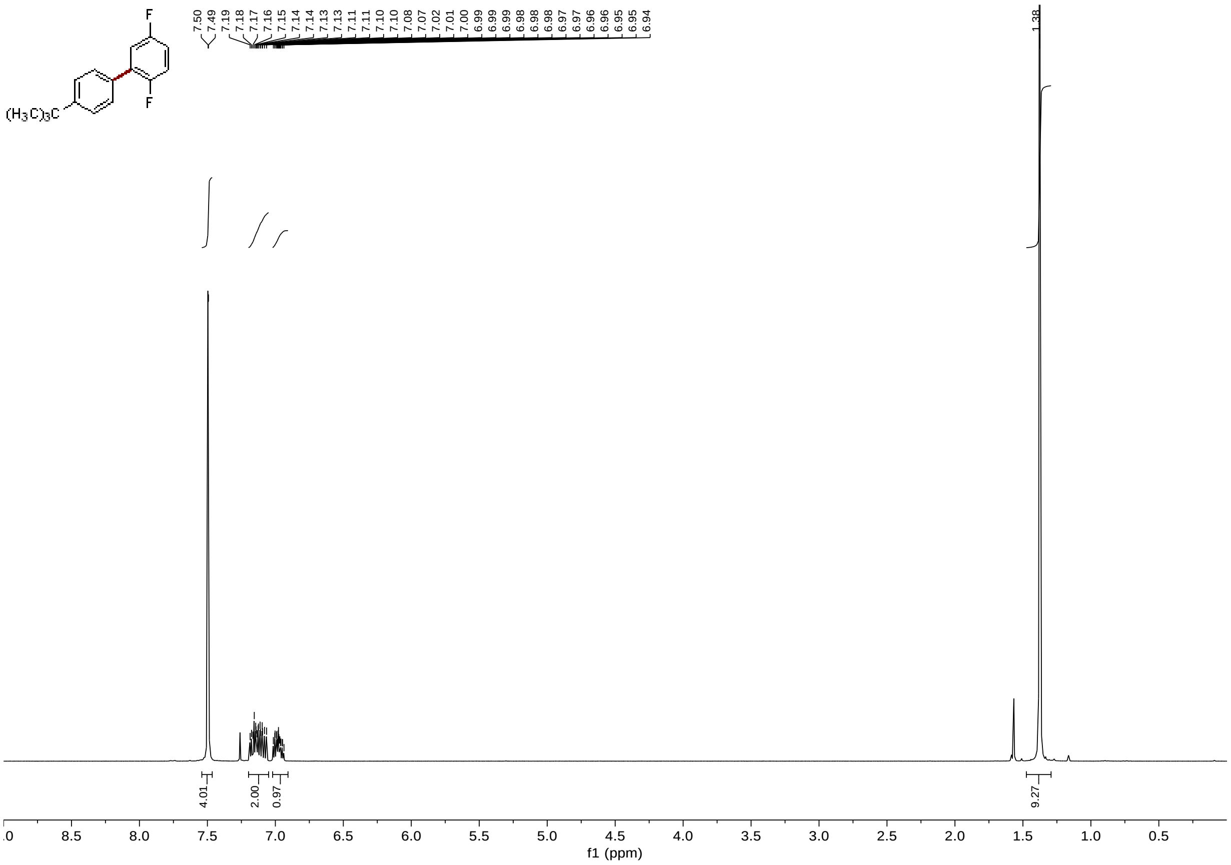
7.50
7.49
7.19
7.18
7.17
7.16
7.15
7.14
7.14
7.13
7.13
7.13
7.11
7.11
7.10
7.10
7.08
7.07
7.02
7.01
7.00
6.99
6.99
6.99
6.98
6.98
6.98
6.97
6.97
6.96
6.96
6.95
6.95
6.94
1.38
4.01
2.00
0.97
9.27
8.5
8.0
7.5
7.0
6.5
6.0
5.5
5.0
4.5
4.0
3.5
3.0
2.5
2.0
1.5
1.0
0.5
f1 (ppm)

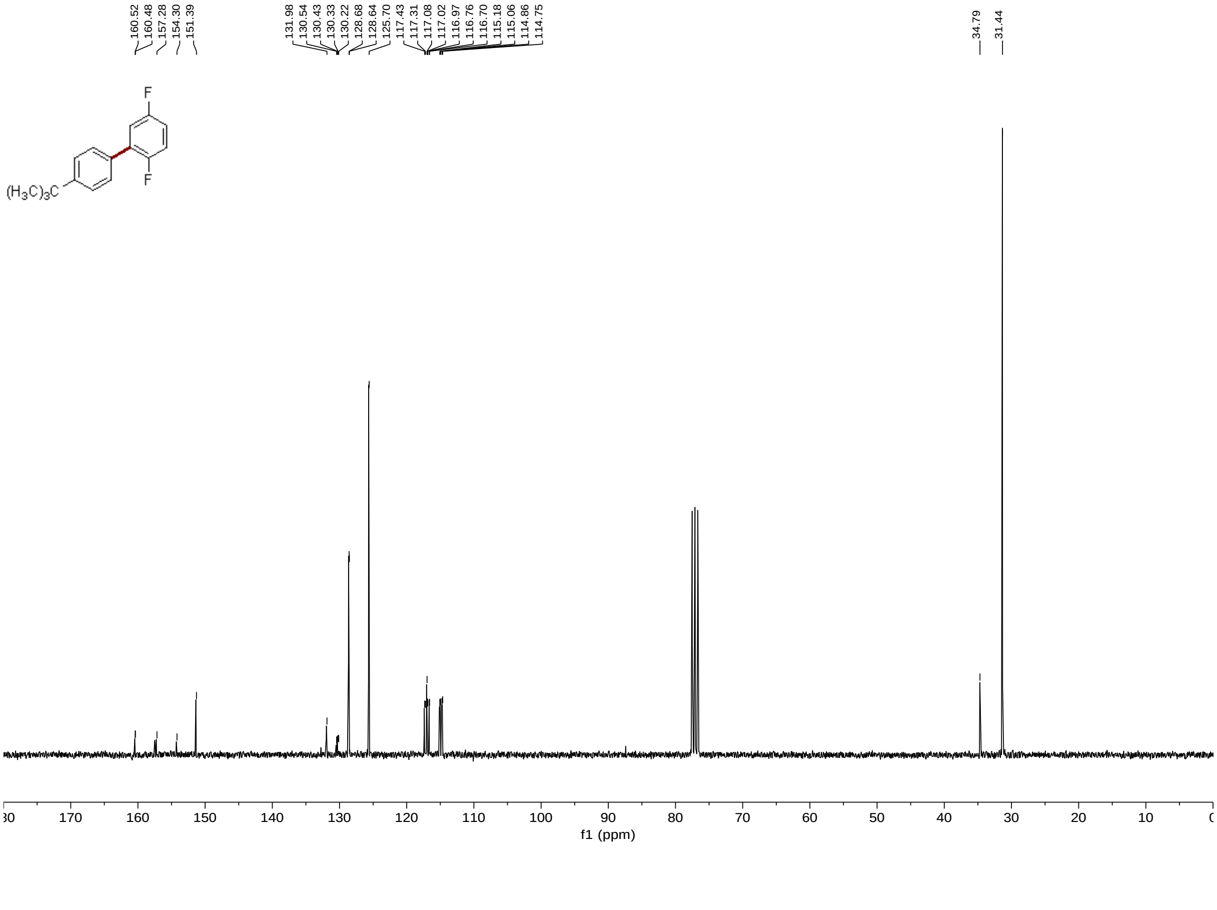
160.52
160.48
157.28
154.30
151.39
131.98
130.54
130.43
130.33
130.22
128.68
128.64
125.70
117.43
117.31
117.08
117.02
116.97
116.76
116.70
115.18
115.06
114.86
114.75
34.79
31.44
170
160
150
140
130
120
110
100
90
80
70
60
50
40
30
20
10
f1 (ppm)

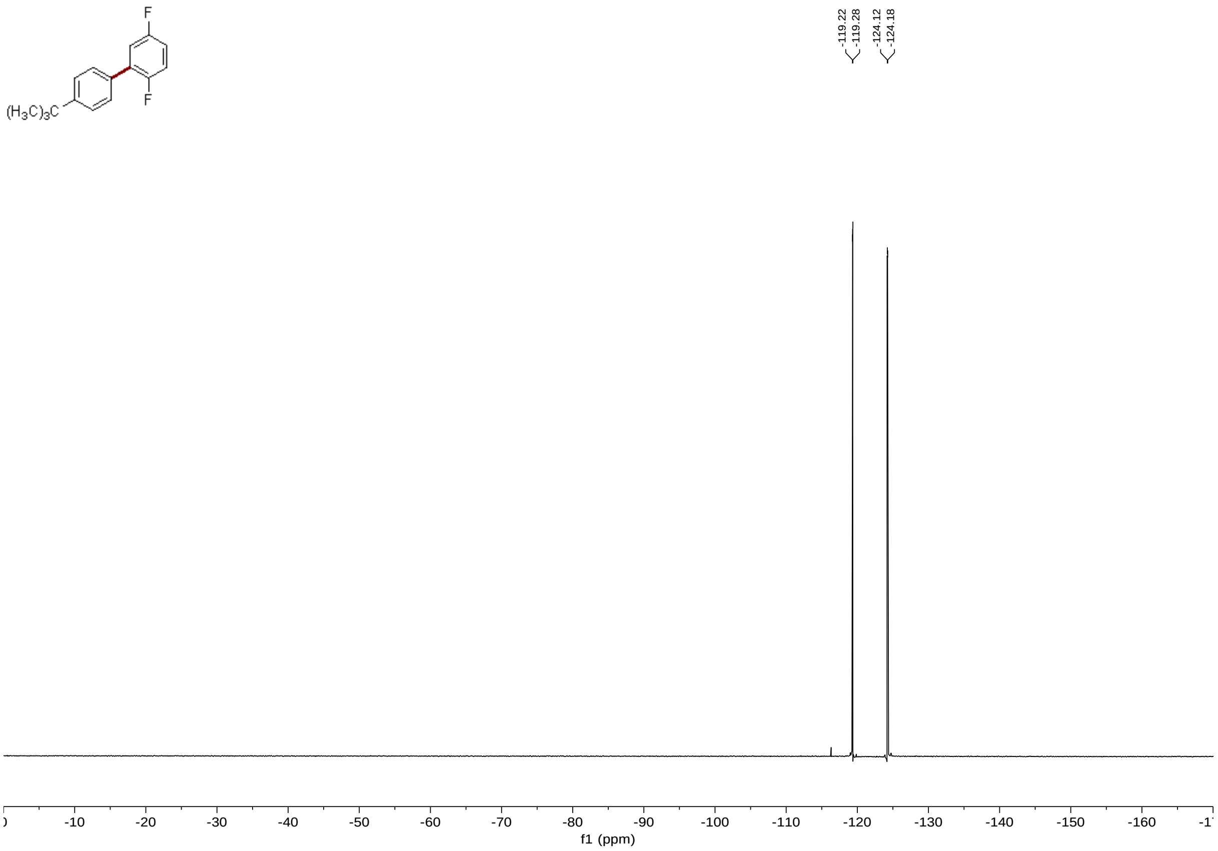

F
F
(H3C)3C
-119.22
-119.28
-124.12
-124.18
f1 (ppm)

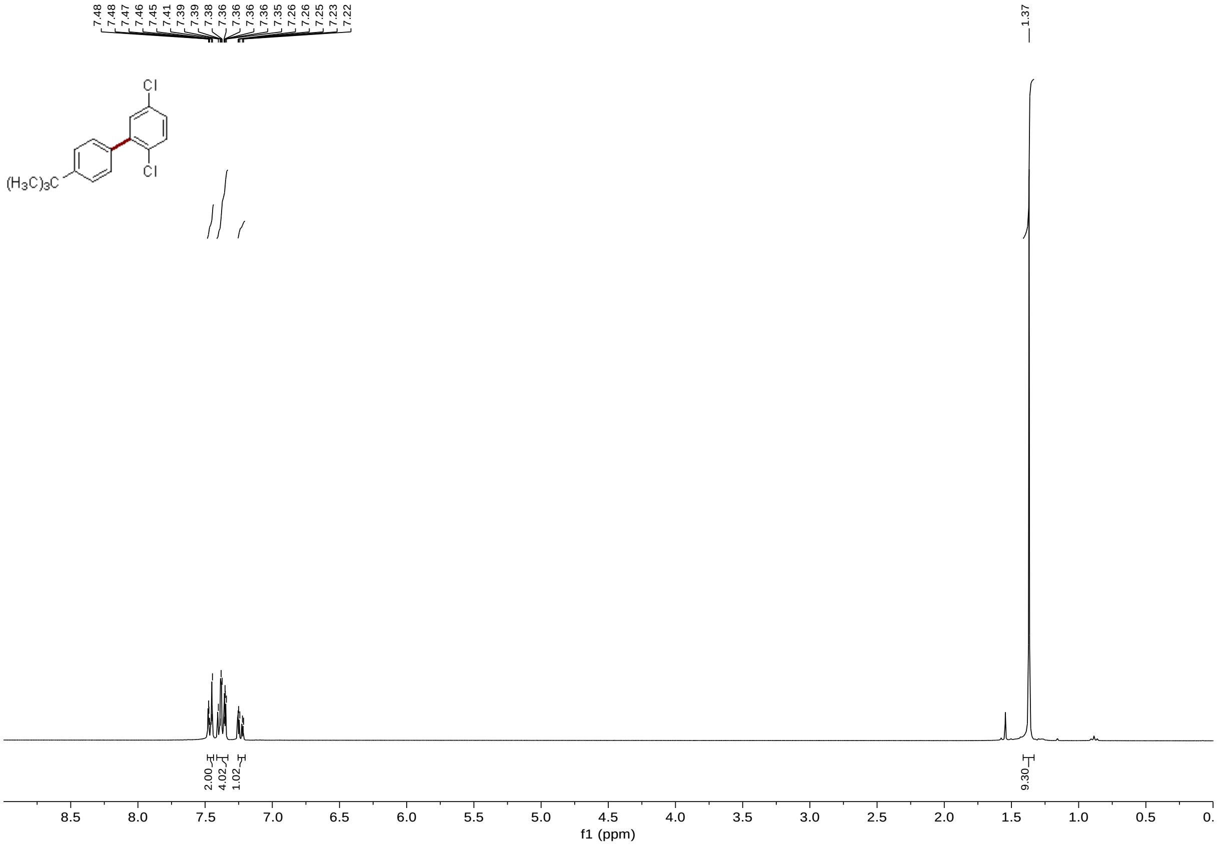

Cl
Cl
(H3C)3C
7.48 7.48 7.47 7.46 7.45 7.41 7.39 7.39 7.38 7.36 7.36 7.36 7.36 7.35 7.26 7.26 7.25 7.23 7.22
1.37
2.00
4.02
1.02
9.30
f1 (ppm)

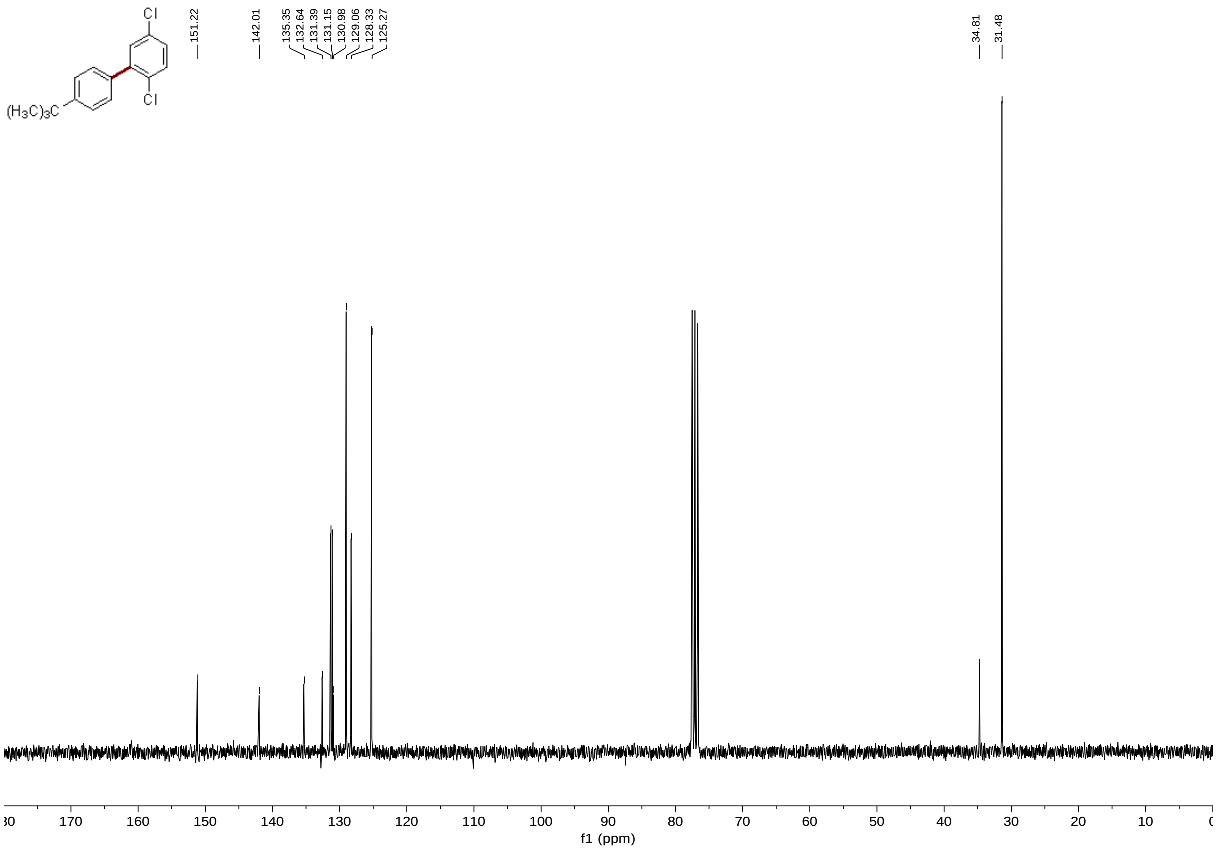
Cl
Cl
(H3C)3C
151.22
142.01
135.35
132.64
131.39
131.15
130.98
129.06
128.33
125.27
34.81
31.48
f1 (ppm)

3x as a mixture of C2/C3/C4 isomers (21:50:29)

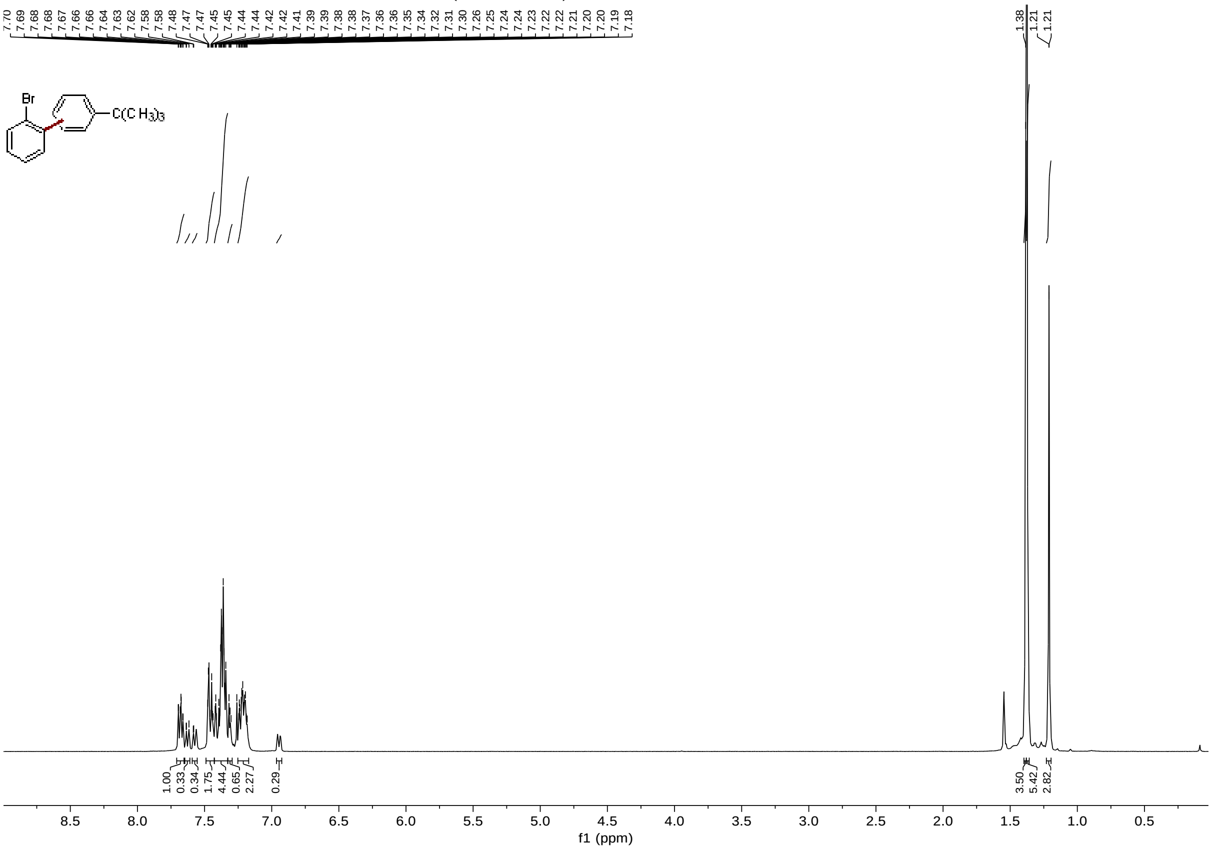

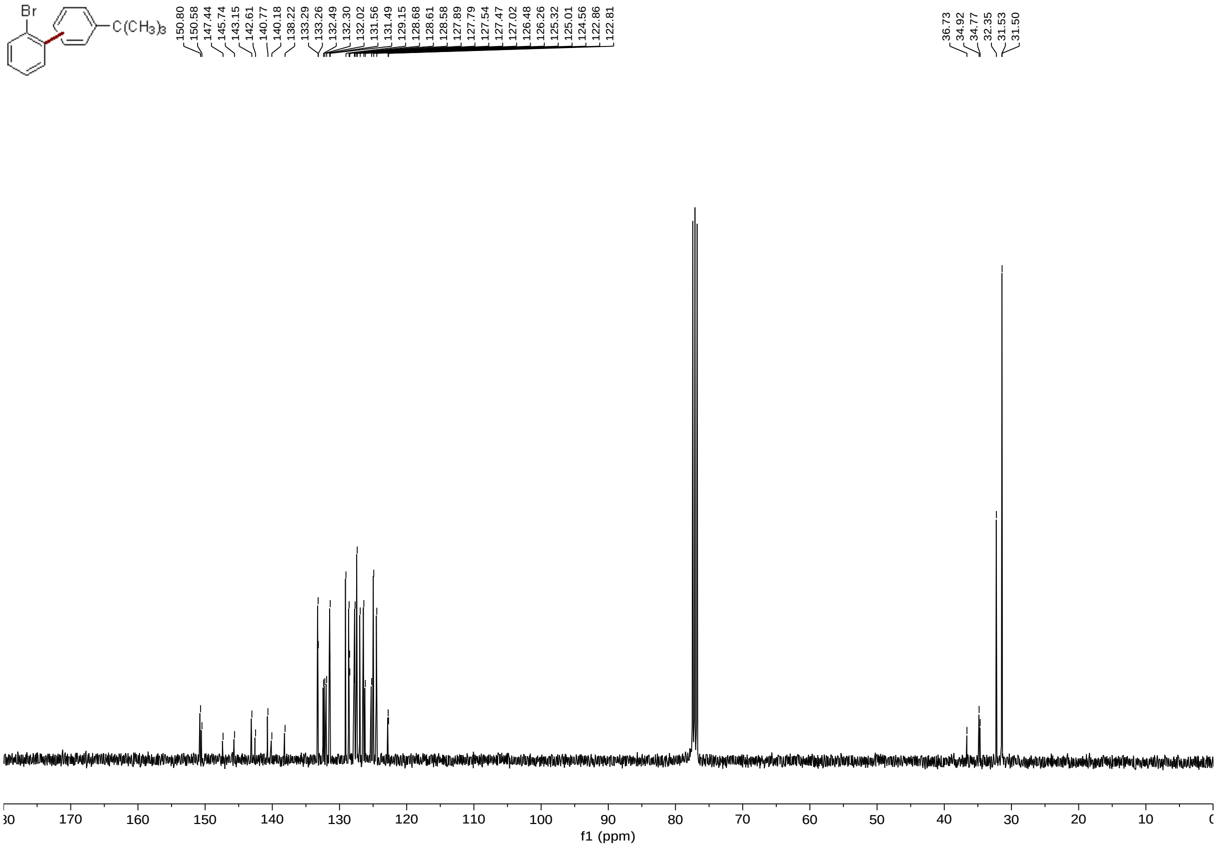

3a as a mixture of C2/C3/C4 isomers (12:57:31)

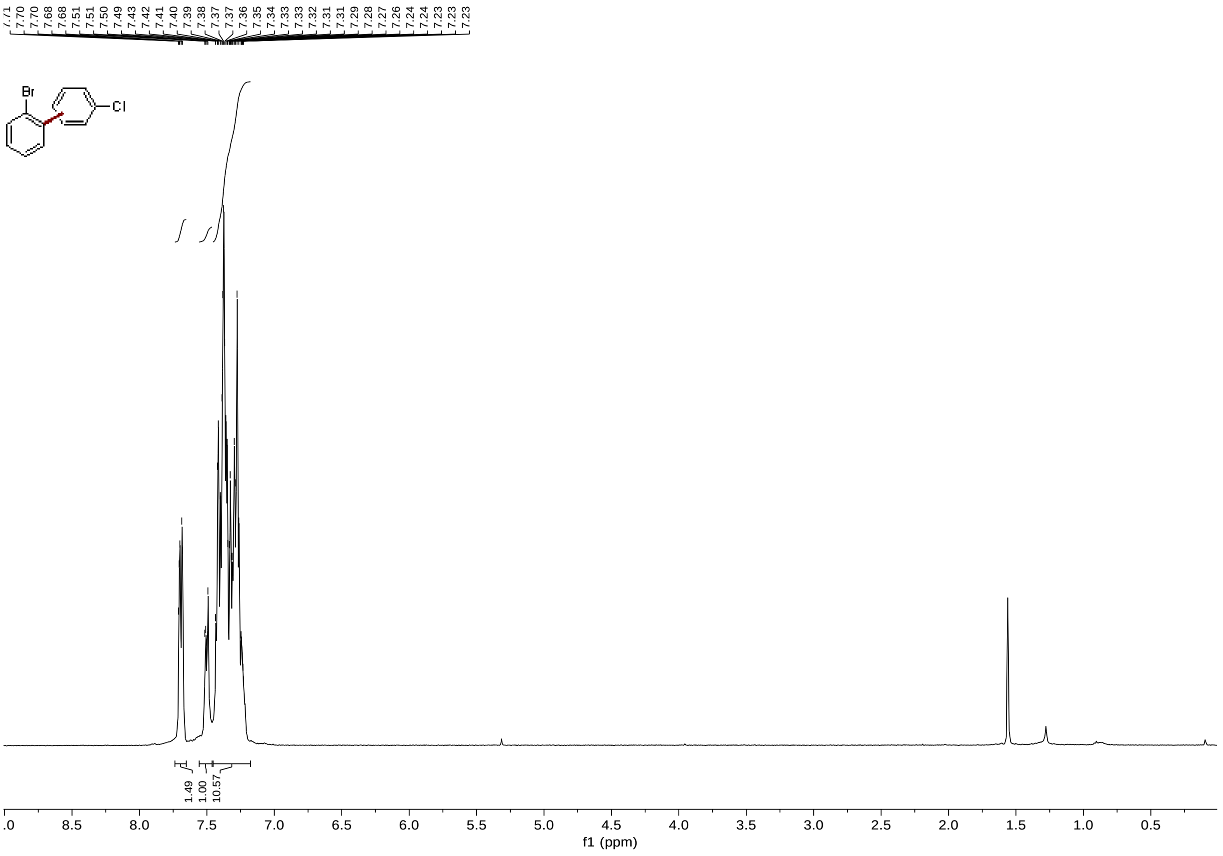

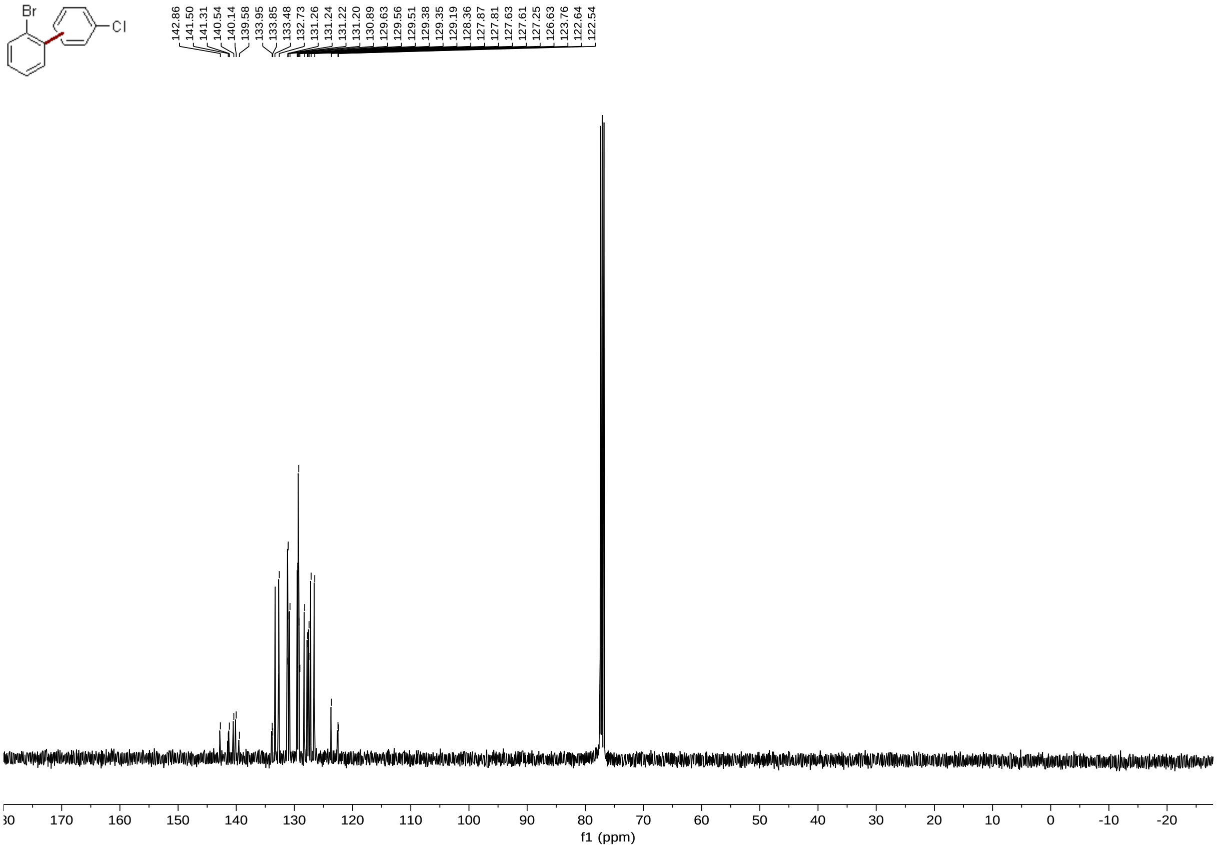

3y as a mixture of C2/C3/C4 isomers (5:60:35)

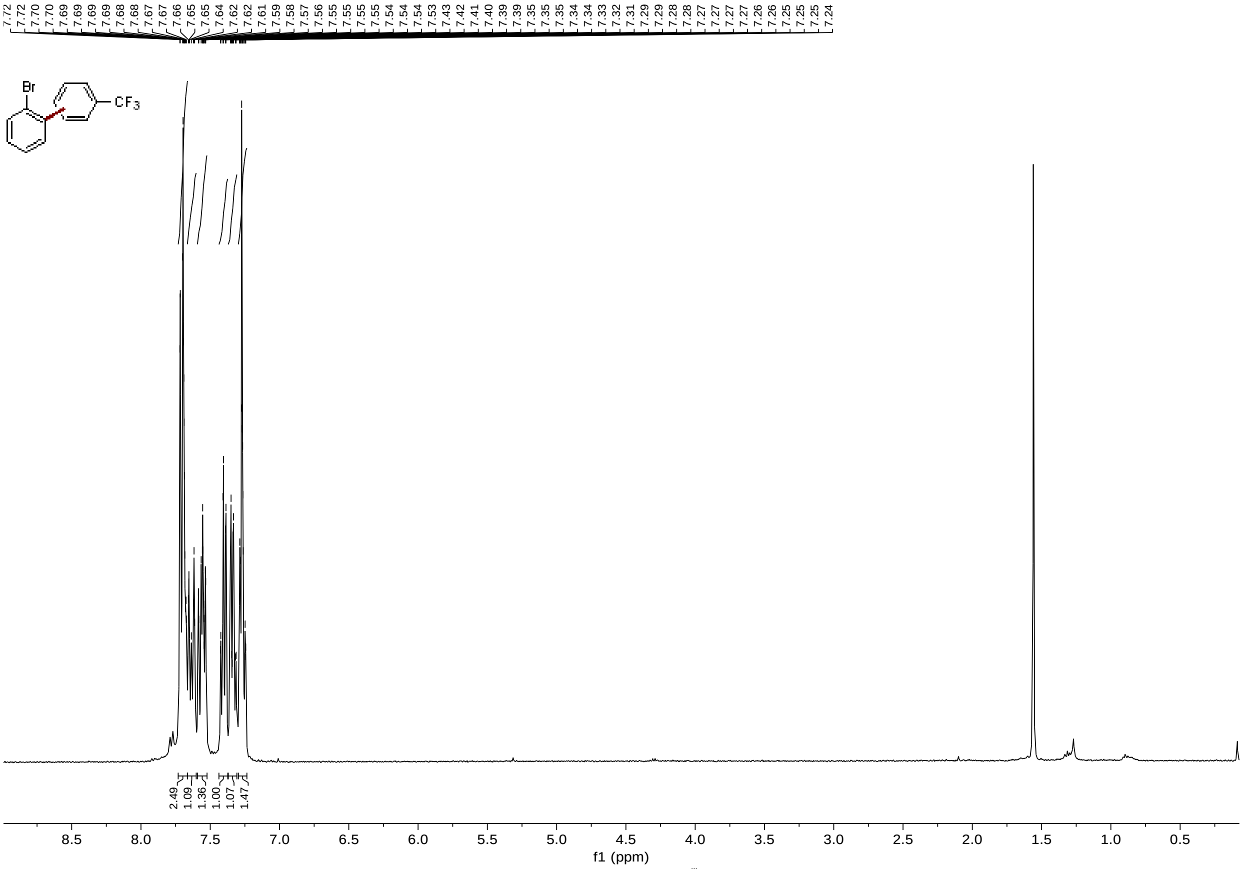

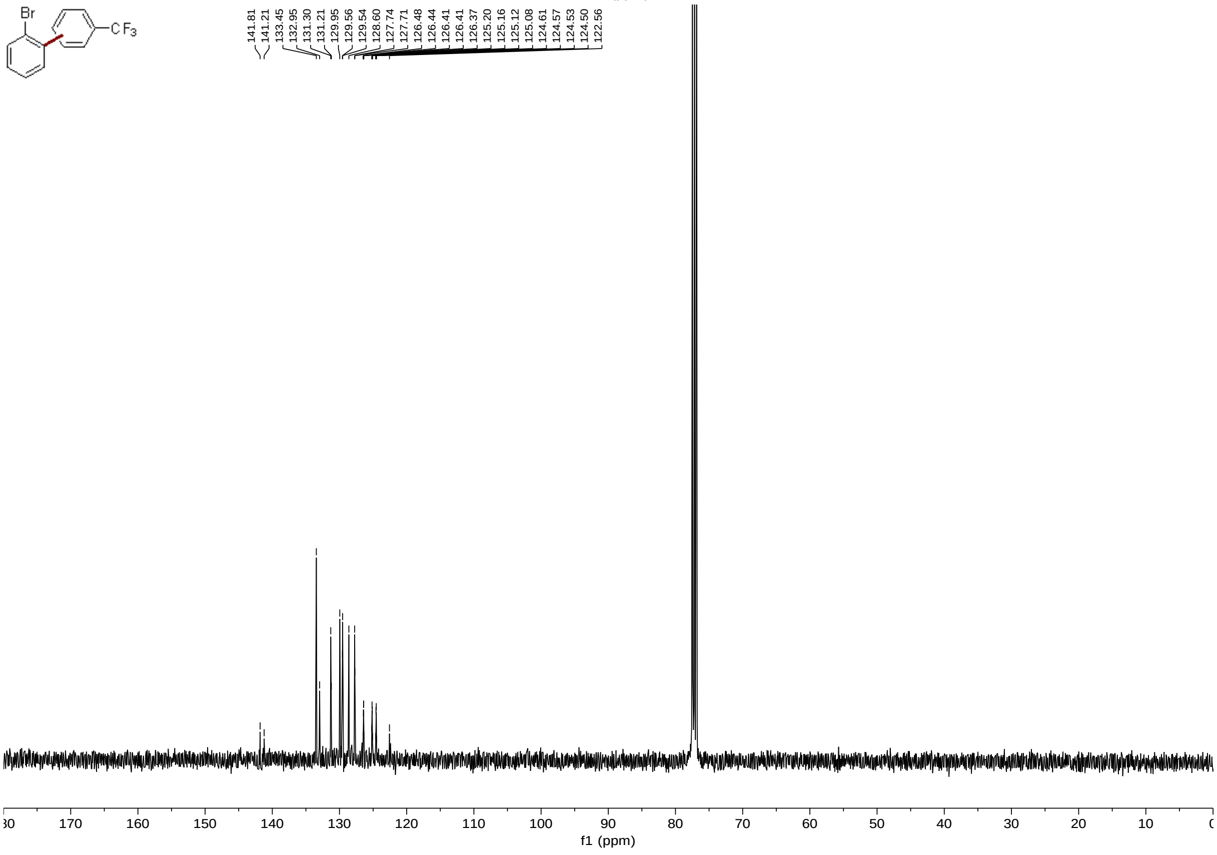

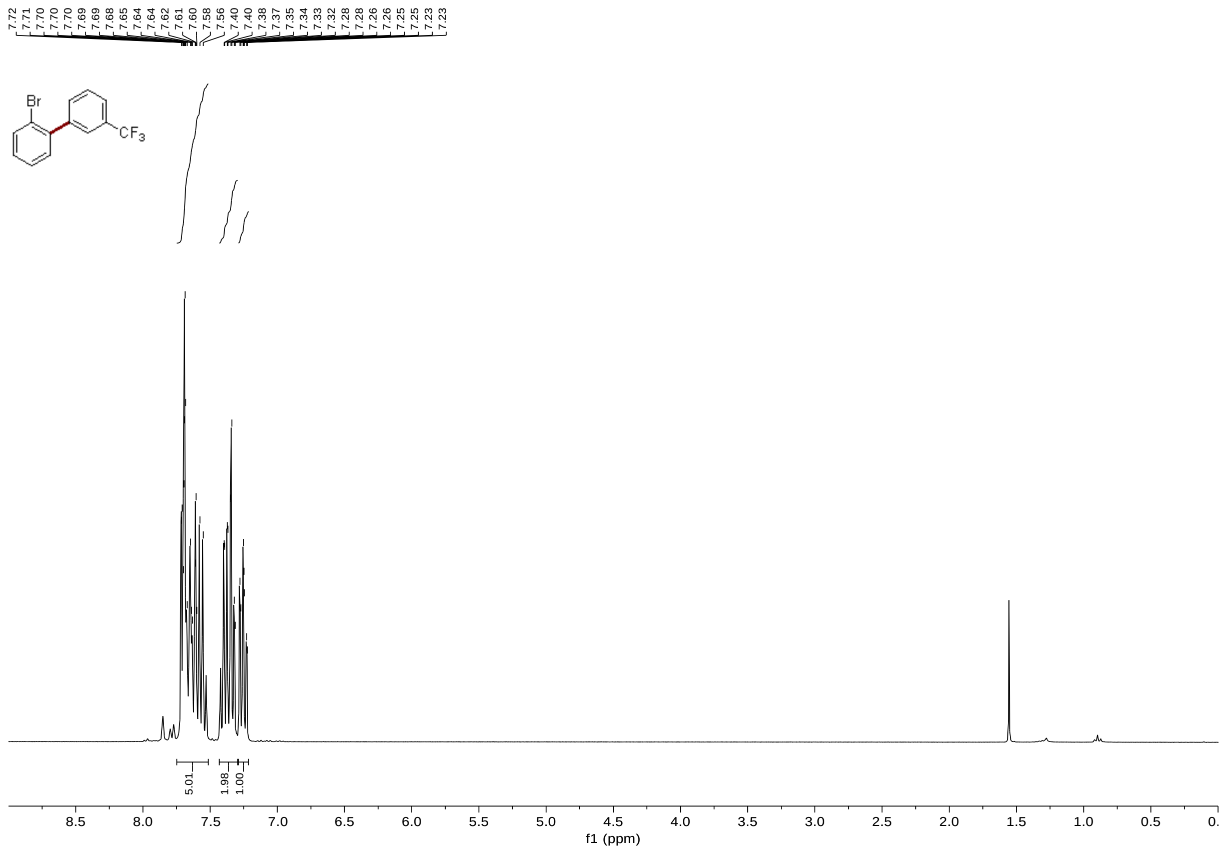
7.72 7.71 7.70 7.70 7.70 7.69 7.69 7.68 7.65 7.64 7.64 7.62 7.61 7.60 7.58 7.56 7.40 7.40 7.38 7.37 7.35 7.34 7.33 7.32 7.28 7.28 7.26 7.26 7.25 7.25 7.23 7.23
Br
CF3
5.01
1.98
1.00
f1 (ppm)

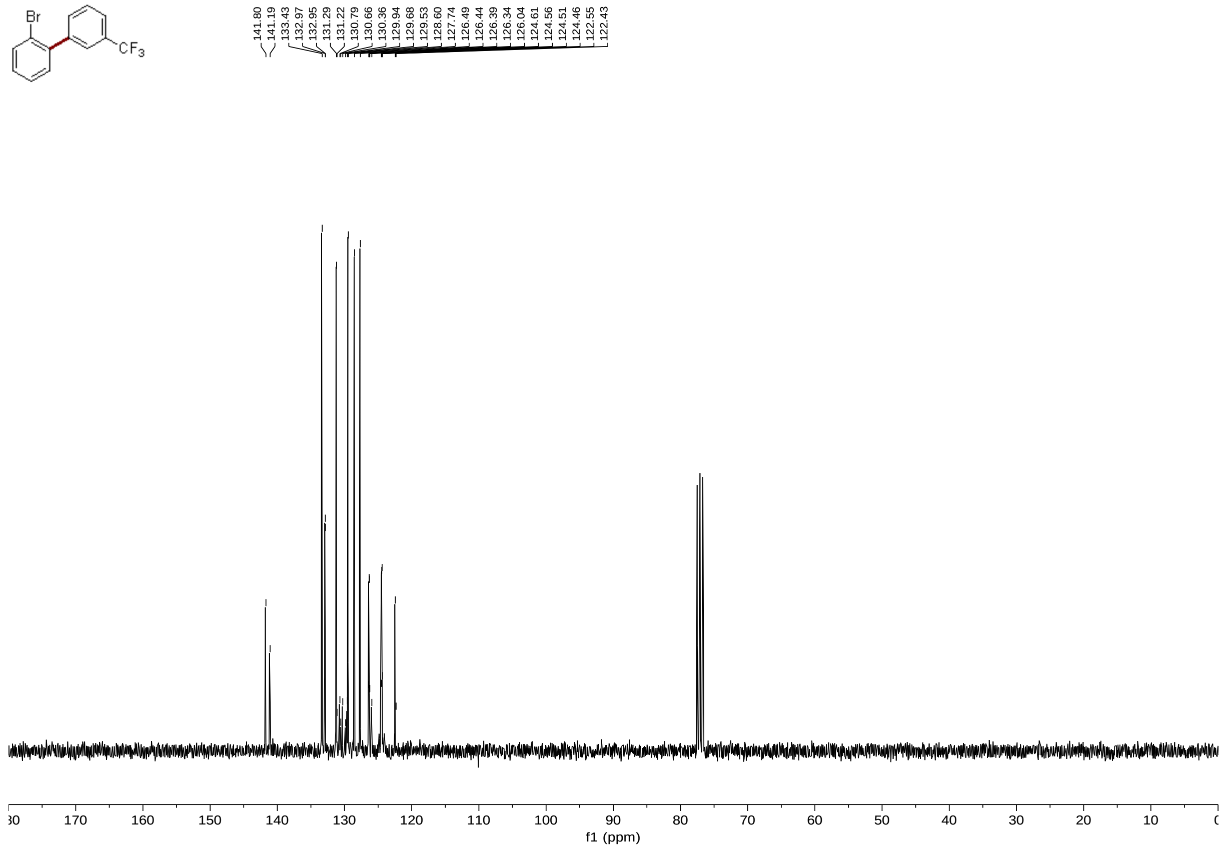
Br
CF3
141.80 141.19 133.43 132.97 132.95 131.29 131.22 130.79 130.66 130.36 129.94 129.68 129.53 128.60 127.74 126.49 126.44 126.39 126.34 126.04 124.61 124.56 124.51 124.46 122.55 122.43
f1 (ppm)

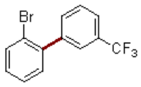
Br
CF3

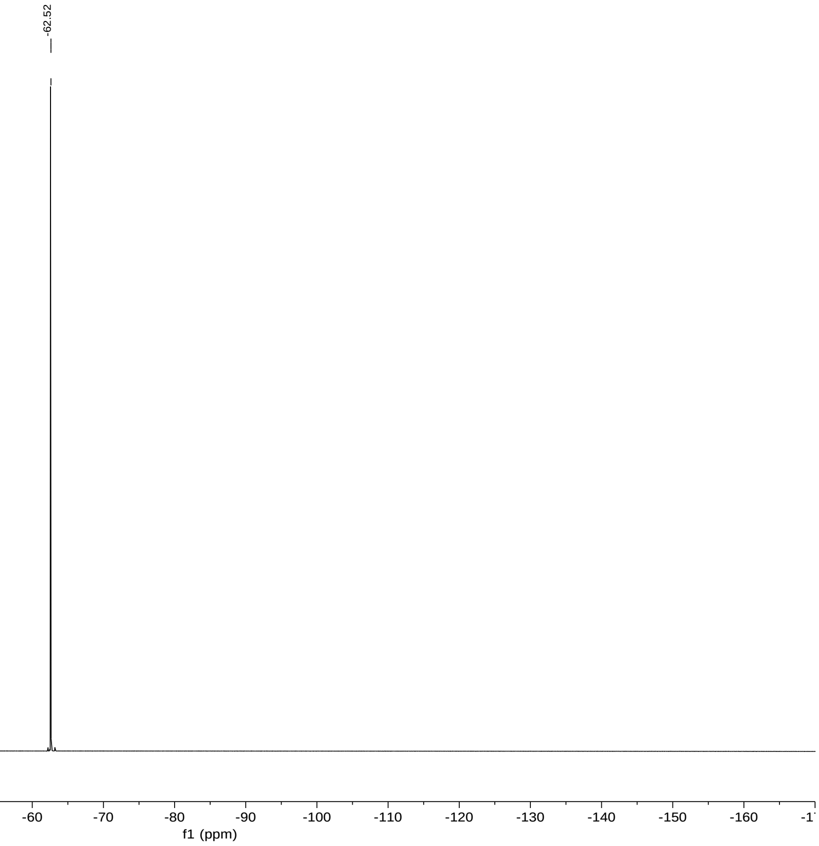
-62.52
-10
-20
-30
-40
-50
-60
-70
-80
-90
-100
-110
-120
-130
-140
-150
-160
f1 (ppm)

3z as a mixture of C2/C3+C4 isomers (62:38), ^{1}H and ^{13}C NMR of C2 isomer given

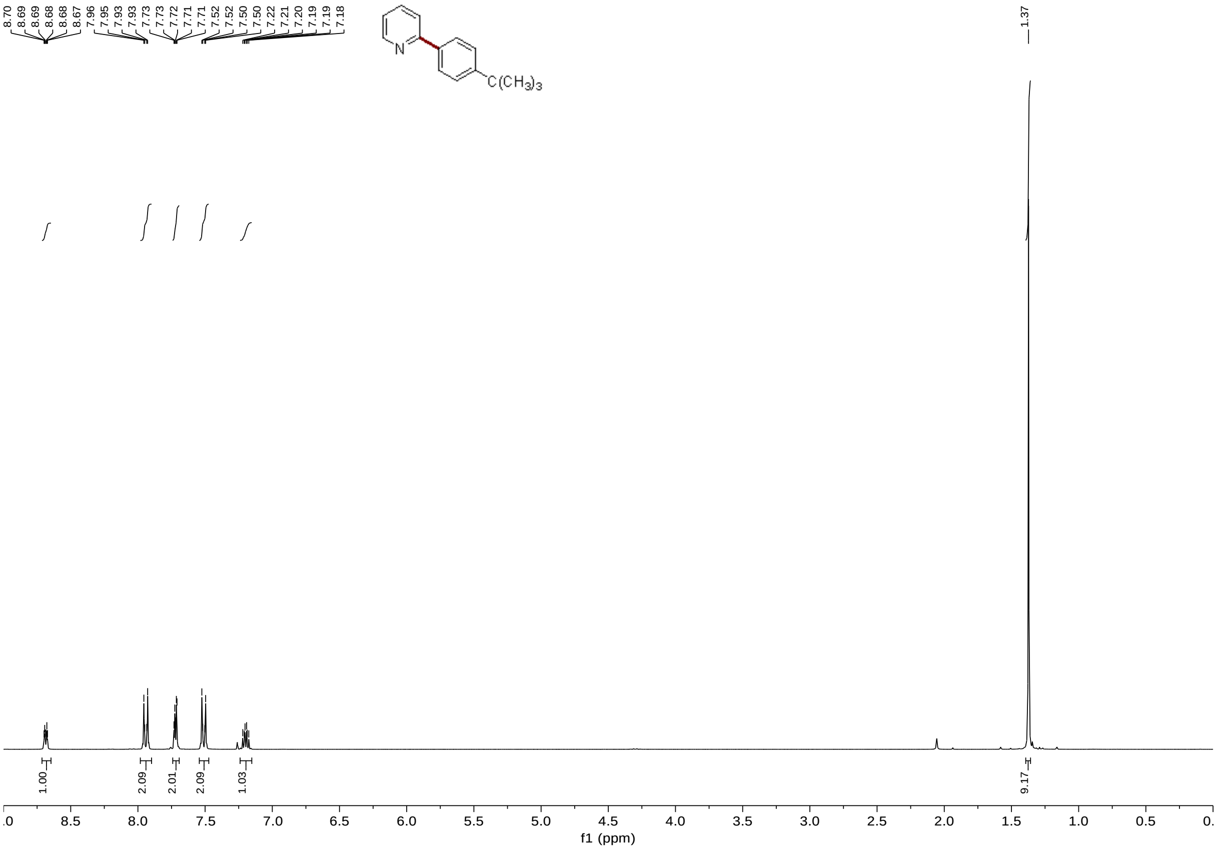

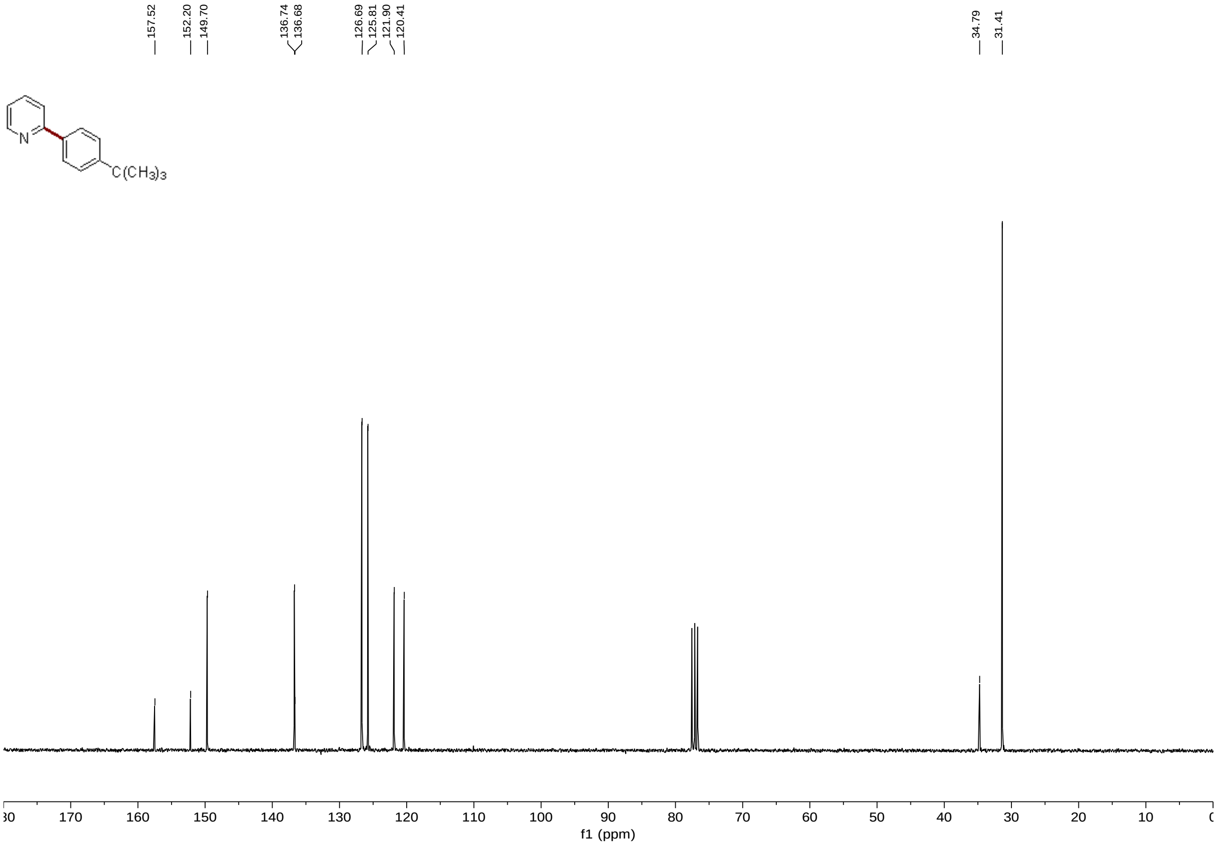

3aa as a mixture of C2/(C3+C4)/C5 isomers (37:22:41)

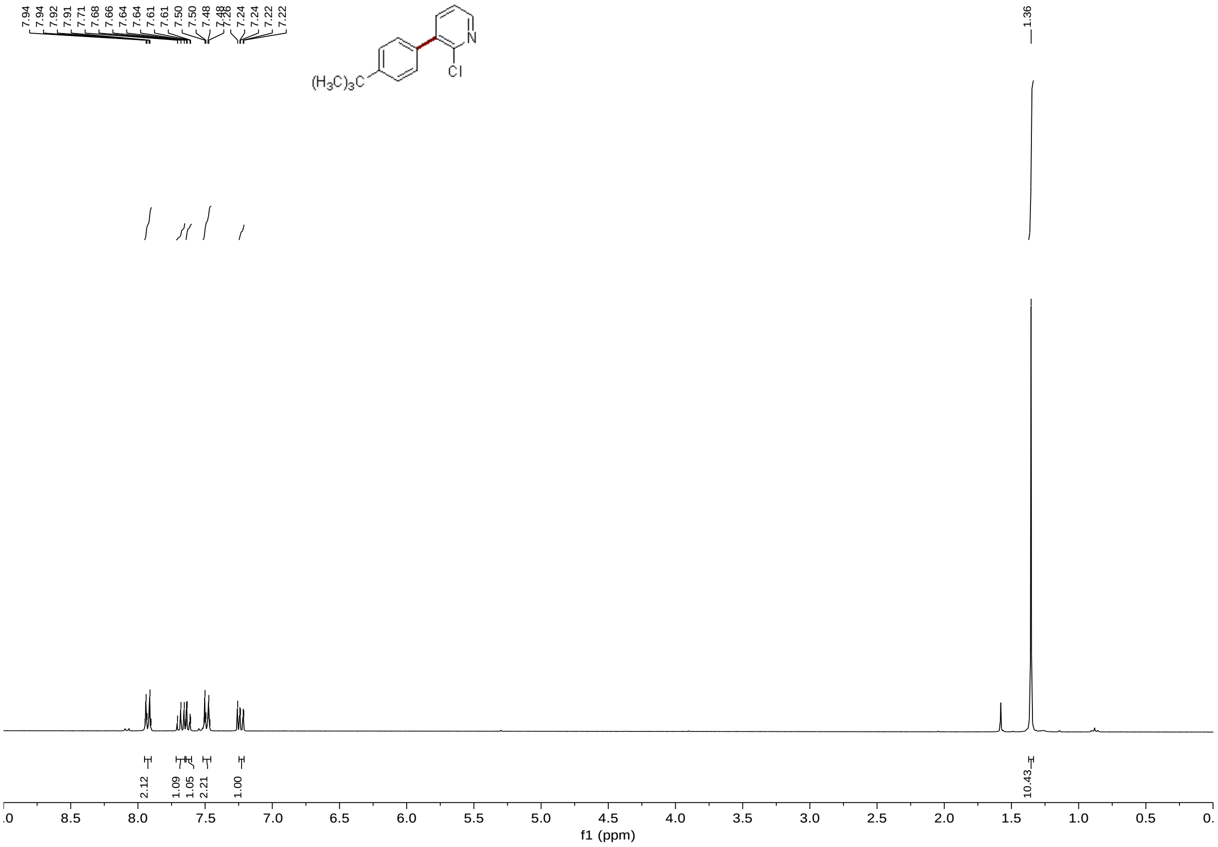

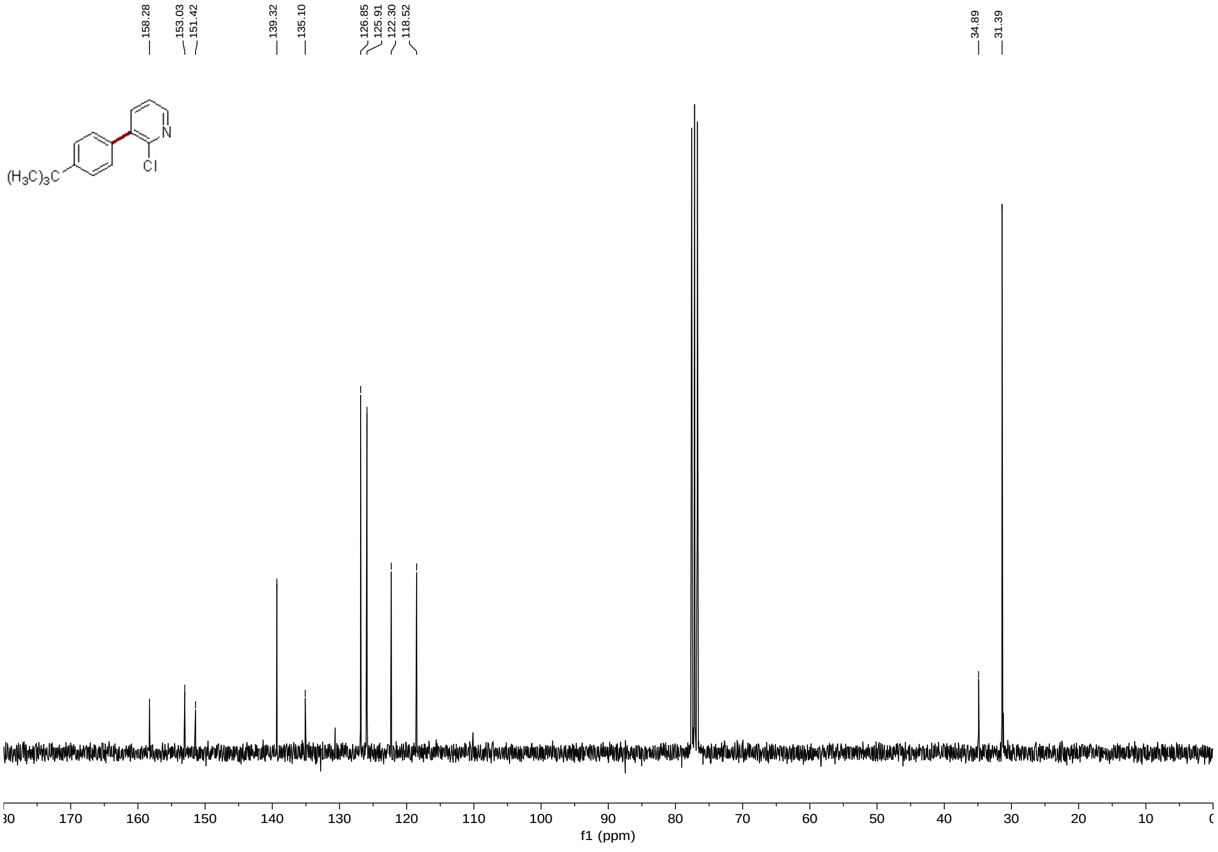

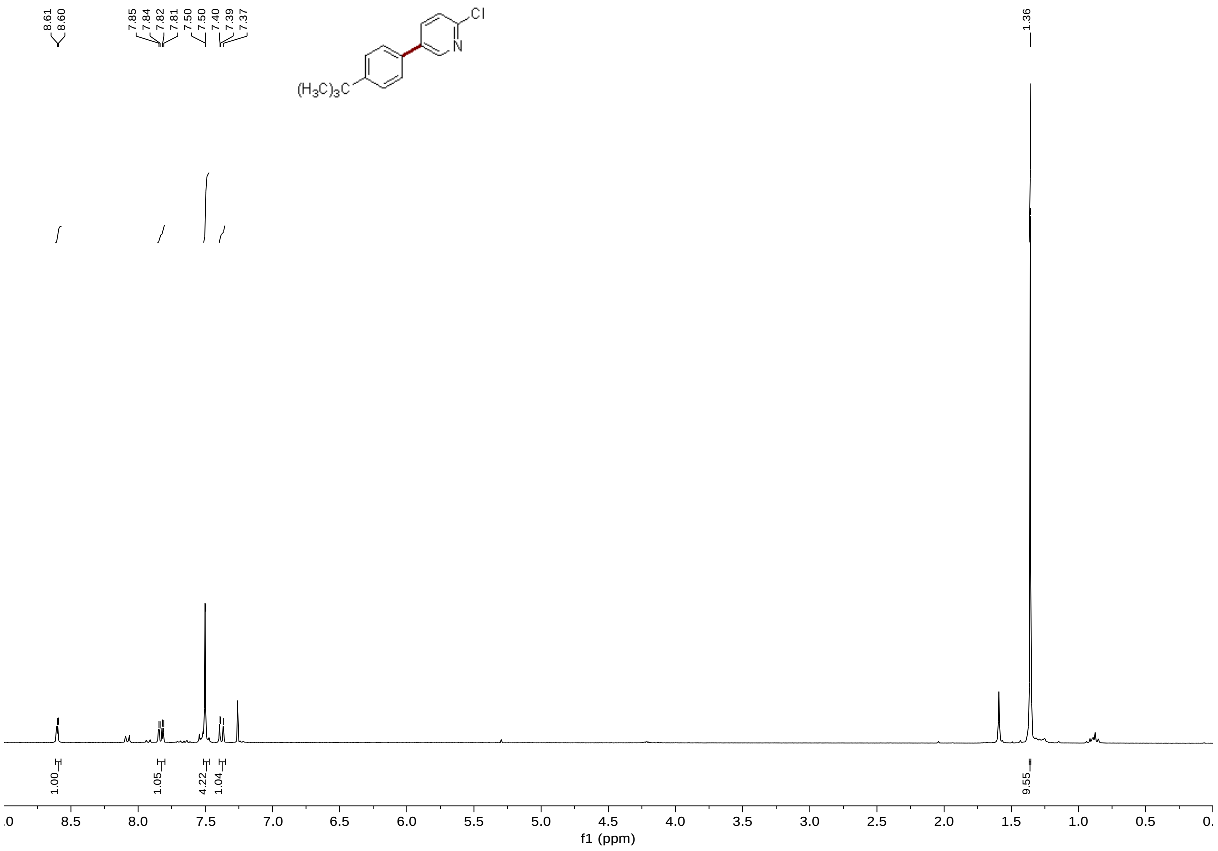
8.61
8.60
7.85
7.84
7.82
7.81
7.50
7.50
7.40
7.39
7.37
1.36
(H3C)3C
Cl
N
1.00
1.05
4.22
1.04
9.55
f1 (ppm)

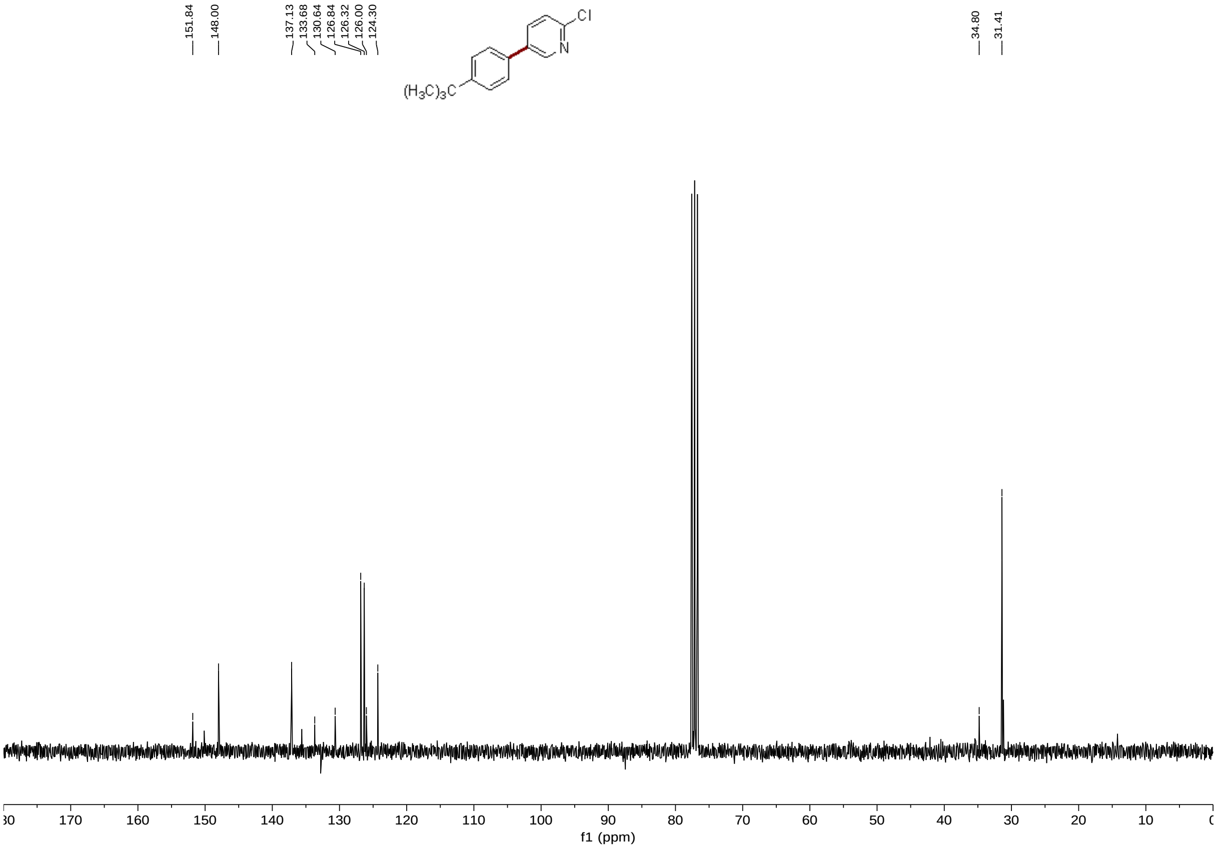
151.84
148.00
137.13
133.68
130.64
126.84
126.32
126.00
124.30
34.80
31.41
(H3C)3C
Cl
N
f1 (ppm)

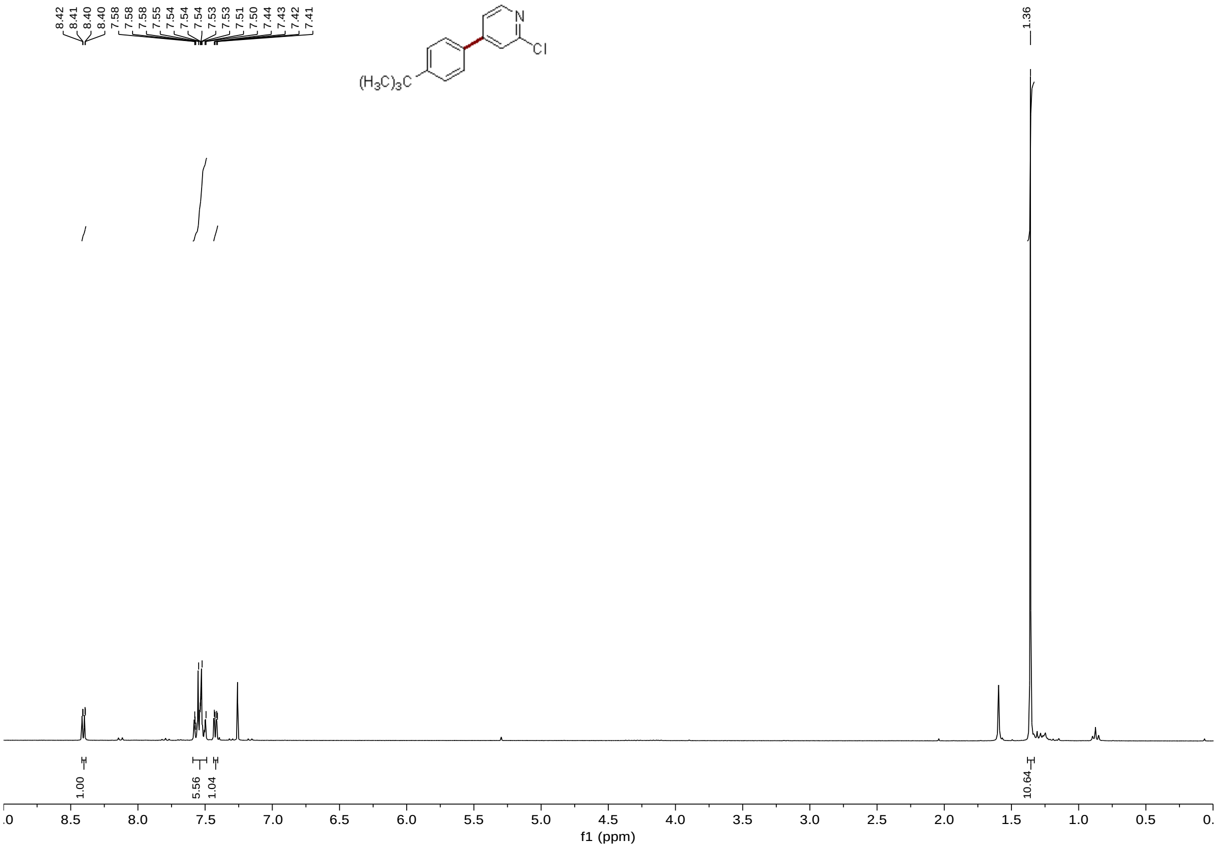
8.42
8.41
8.40
8.40
7.58
7.58
7.55
7.54
7.54
7.54
7.53
7.53
7.51
7.50
7.44
7.43
7.42
7.41
1.36
N
Cl
(H3C)3C
1.00
5.56
1.04
10.64
8.5 8.0 7.5 7.0 6.5 6.0 5.5 5.0 4.5 4.0 3.5 3.0 2.5 2.0 1.5 1.0 0.5
f1 (ppm)

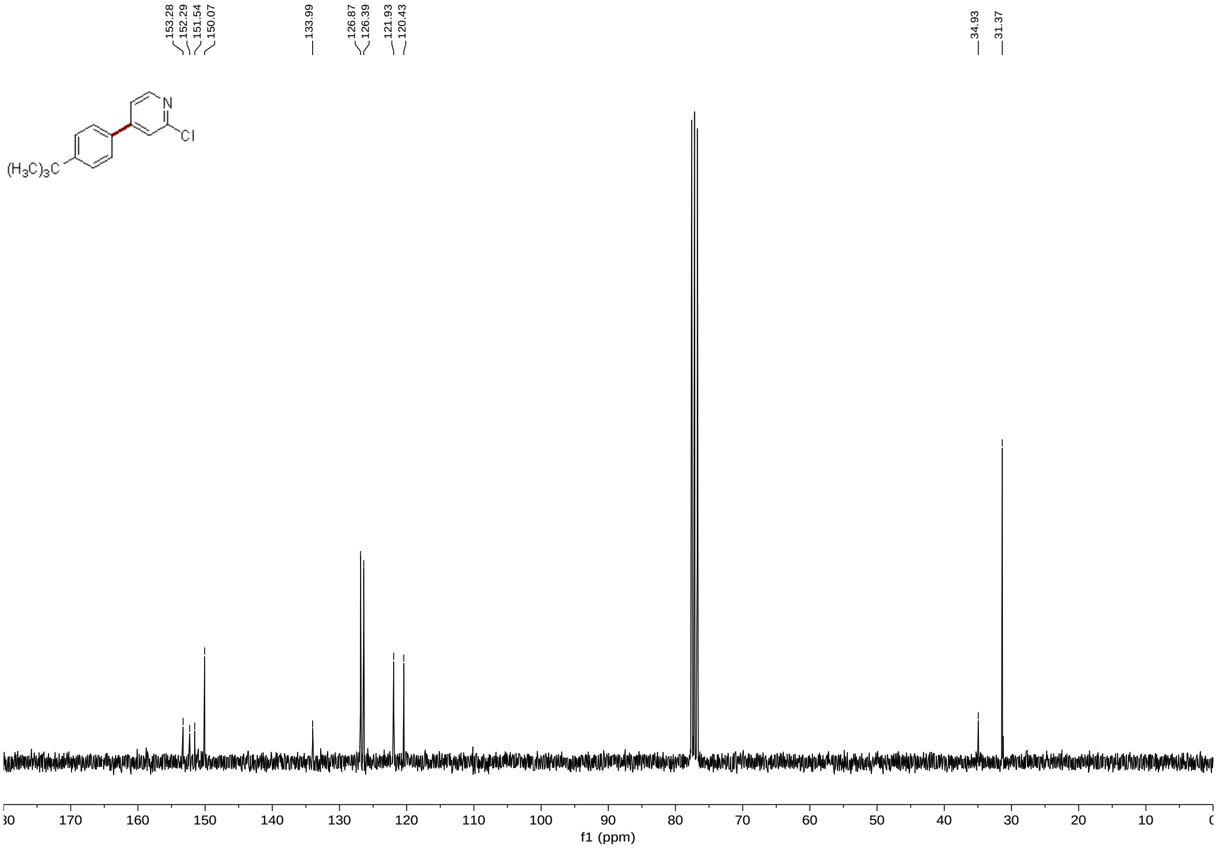
153.28
152.29
151.54
150.07
133.99
126.87
126.39
121.93
120.43
34.93
31.37
N
Cl
(H3C)3C
170 160 150 140 130 120 110 100 90 80 70 60 50 40 30 20 10
f1 (ppm)

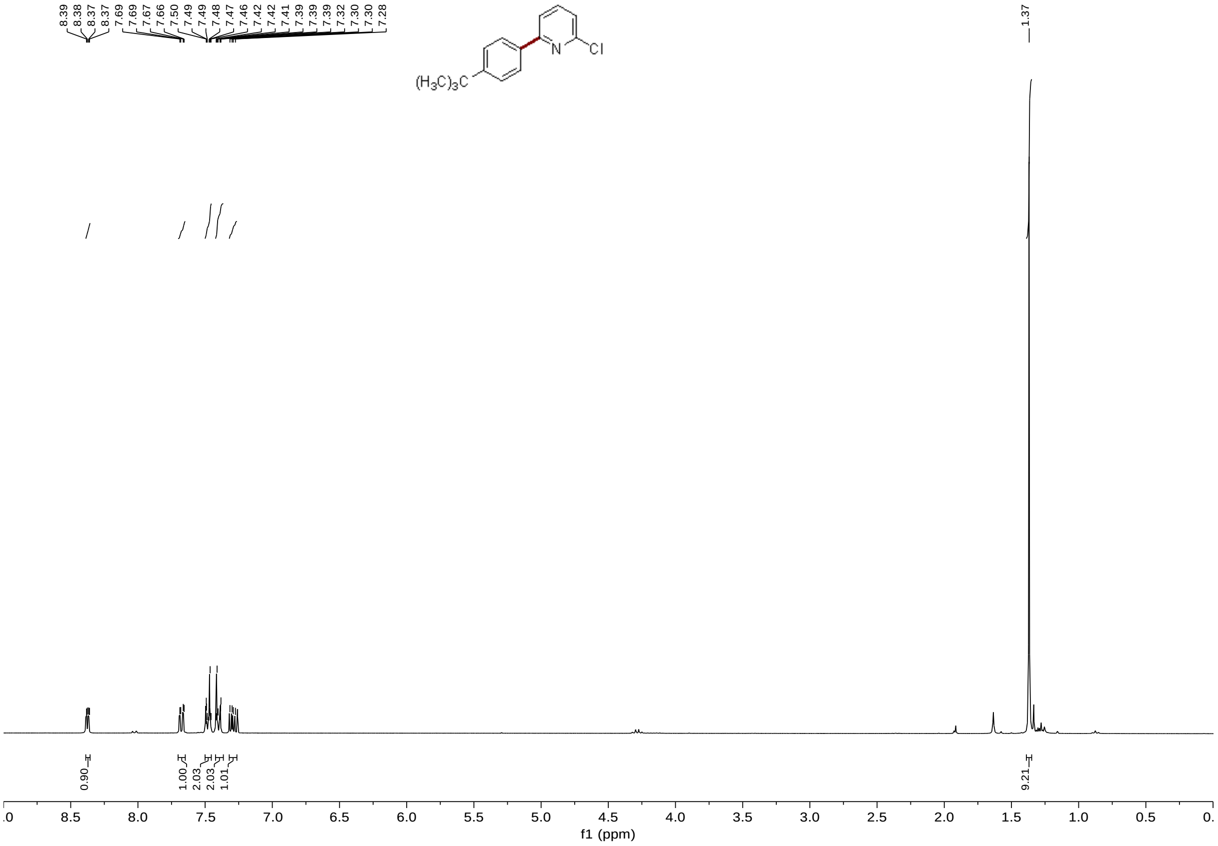

8.39
8.38
8.37
8.37
7.69
7.69
7.67
7.66
7.50
7.49
7.49
7.48
7.47
7.46
7.42
7.42
7.41
7.39
7.39
7.39
7.39
7.32
7.30
7.30
7.28
1.37
$(H_3C)_3C$
N
Cl
0.90
1.00
2.03
2.03
1.01
9.21
f1 (ppm)

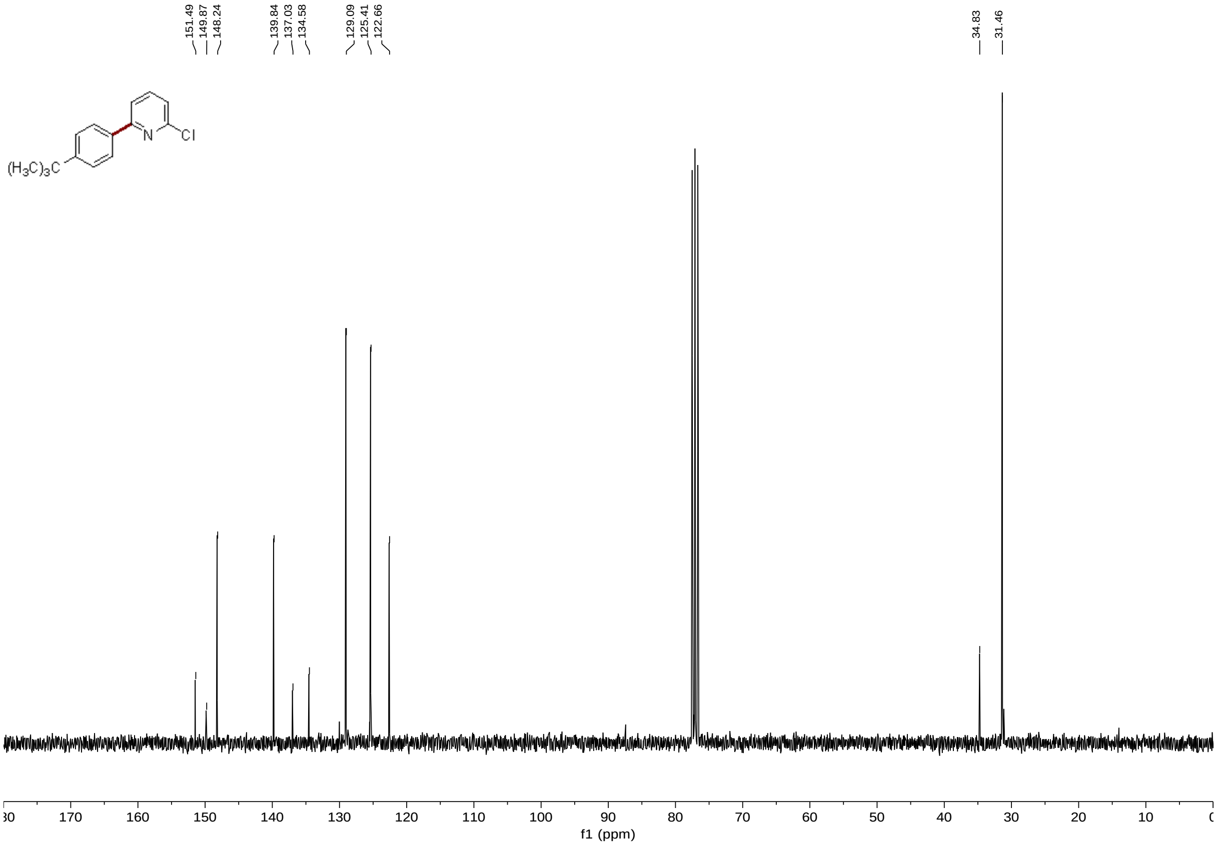

151.49
149.87
148.24
139.84
137.03
134.58
129.09
125.41
122.66
34.83
31.46
$(H_3C)_3C$
N
Cl
f1 (ppm)

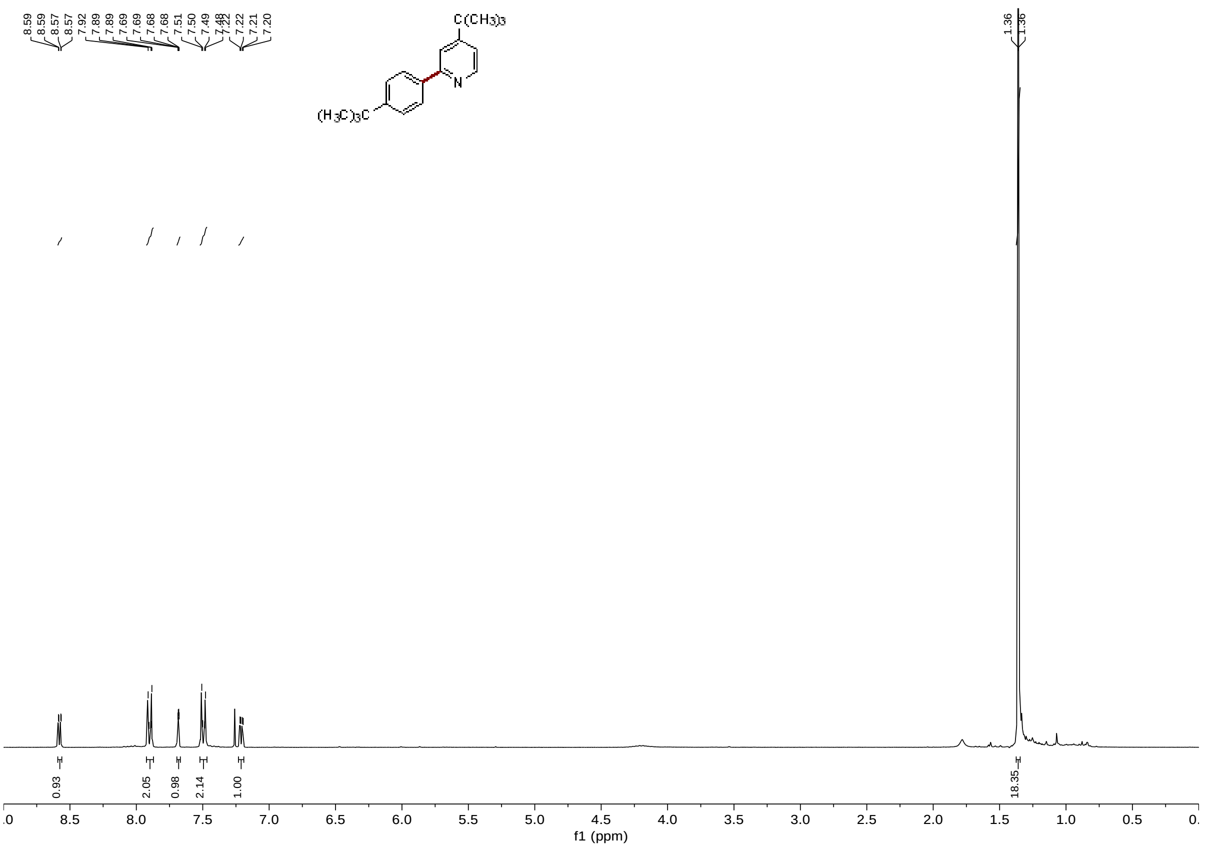

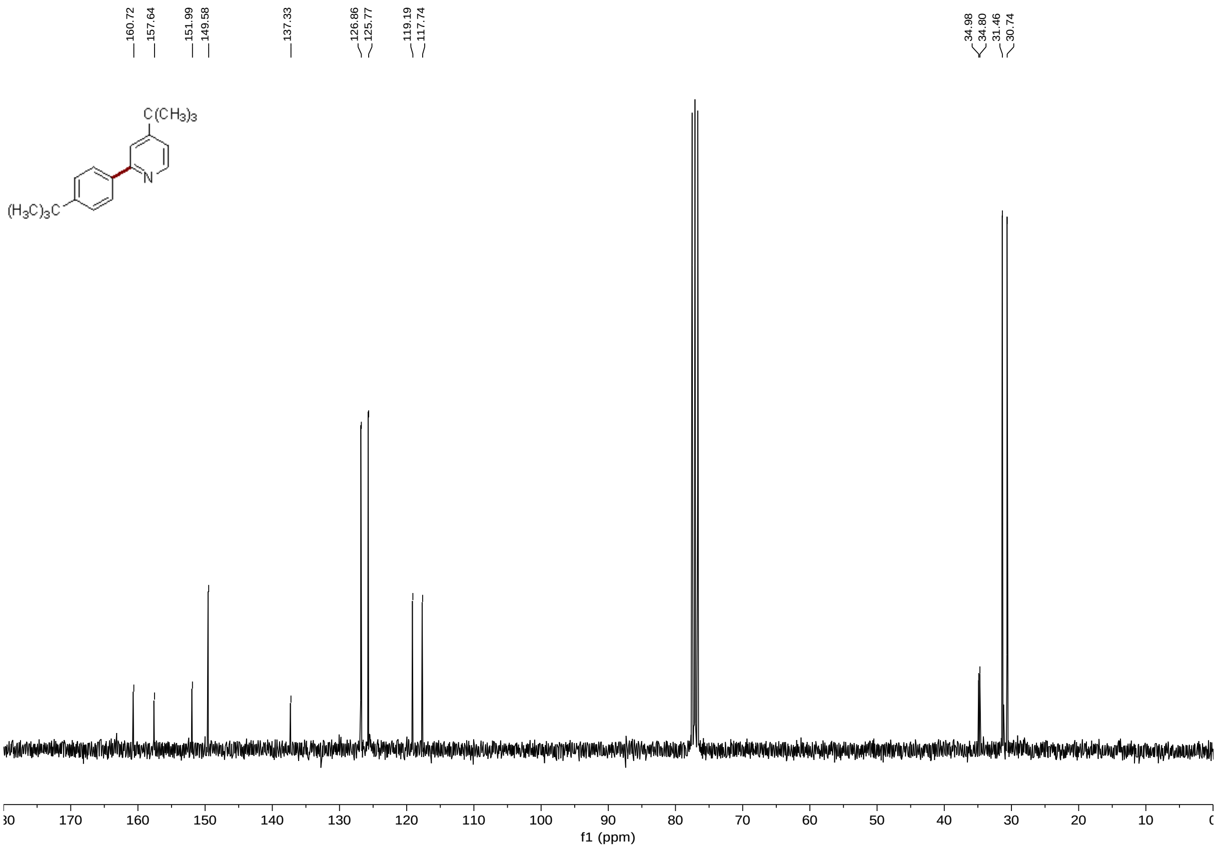

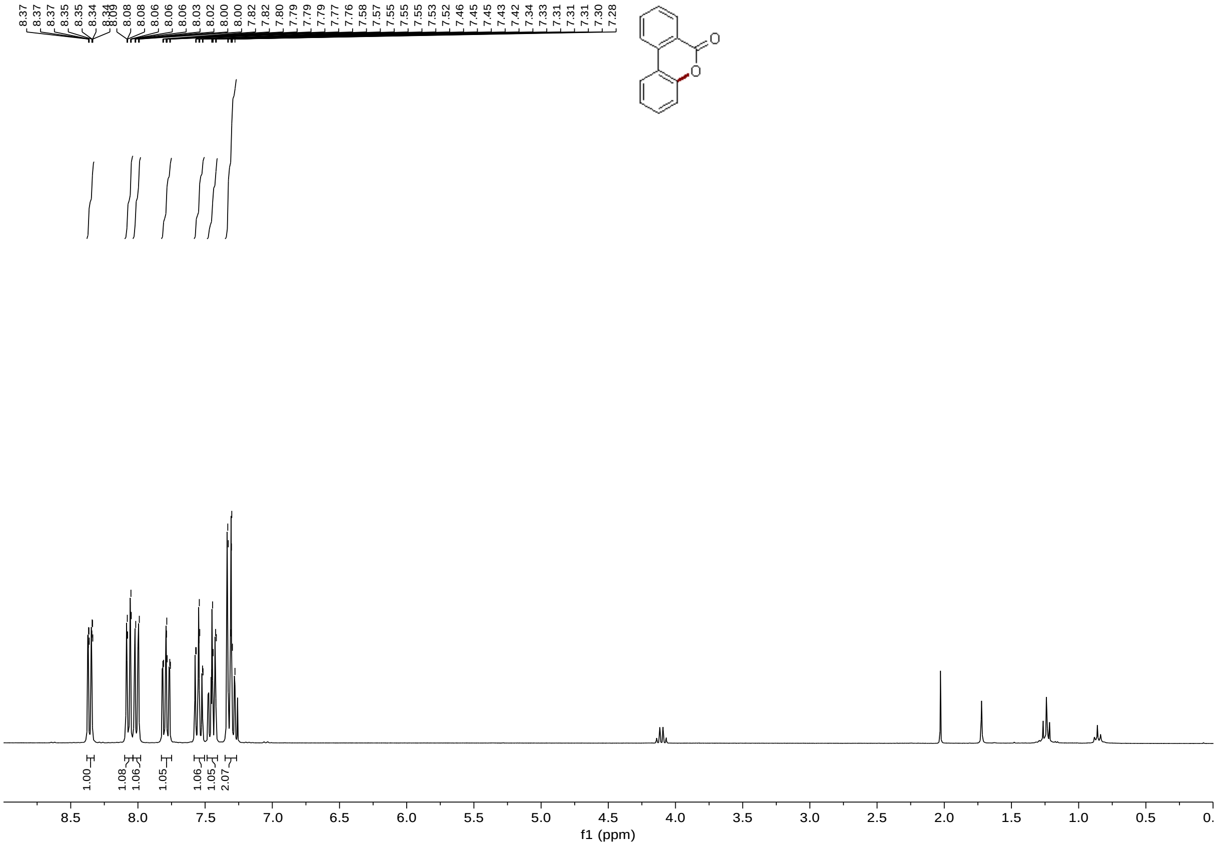
8.37
8.37
8.37
8.35
8.35
8.34
8.34
8.08
8.08
8.06
8.06
8.03
8.02
8.00
8.00
7.82
7.82
7.80
7.79
7.79
7.79
7.77
7.76
7.58
7.57
7.55
7.55
7.53
7.52
7.46
7.45
7.43
7.42
7.34
7.33
7.31
7.31
7.30
7.28
O
O
1.00
1.08
1.06
1.05
1.06
1.05
2.07
f1 (ppm)

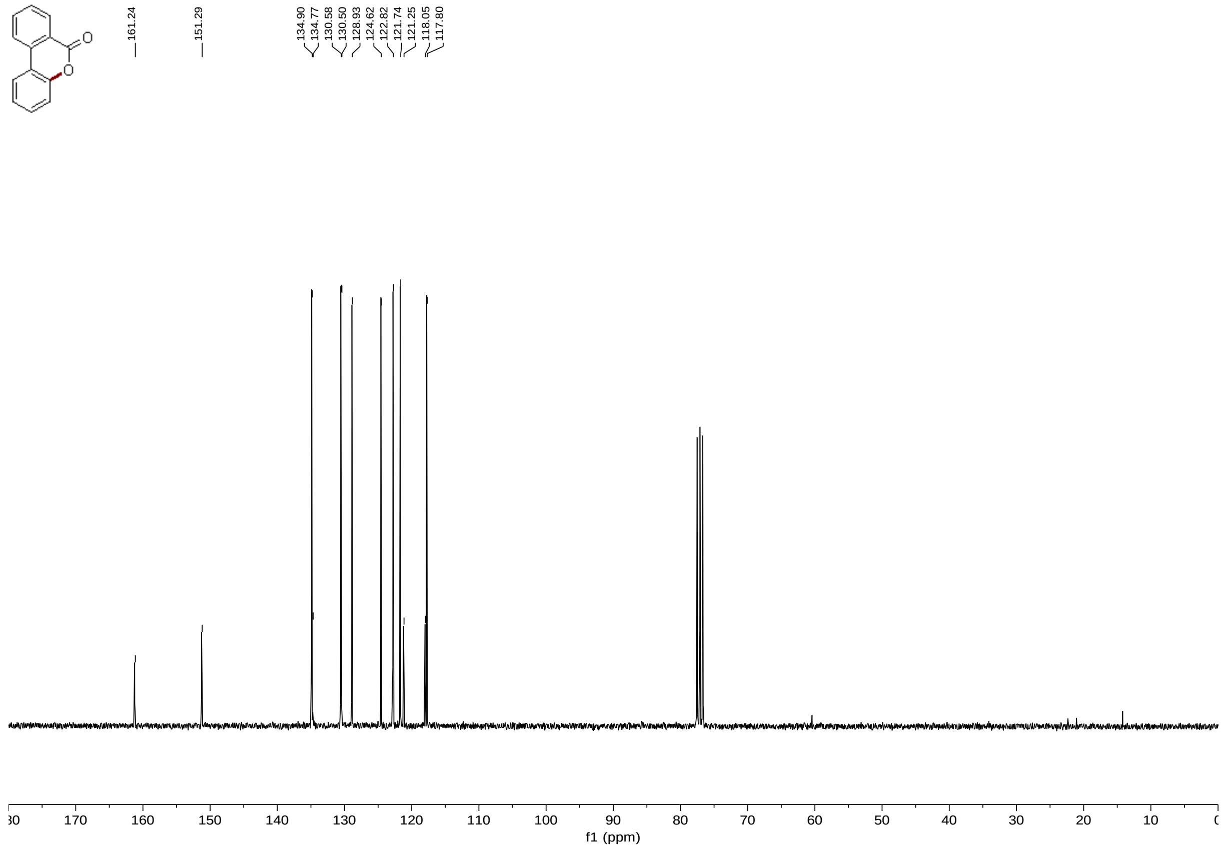
O
O
161.24
151.29
134.90
134.77
130.58
130.50
128.93
124.62
122.82
121.74
121.25
118.05
117.80
f1 (ppm)

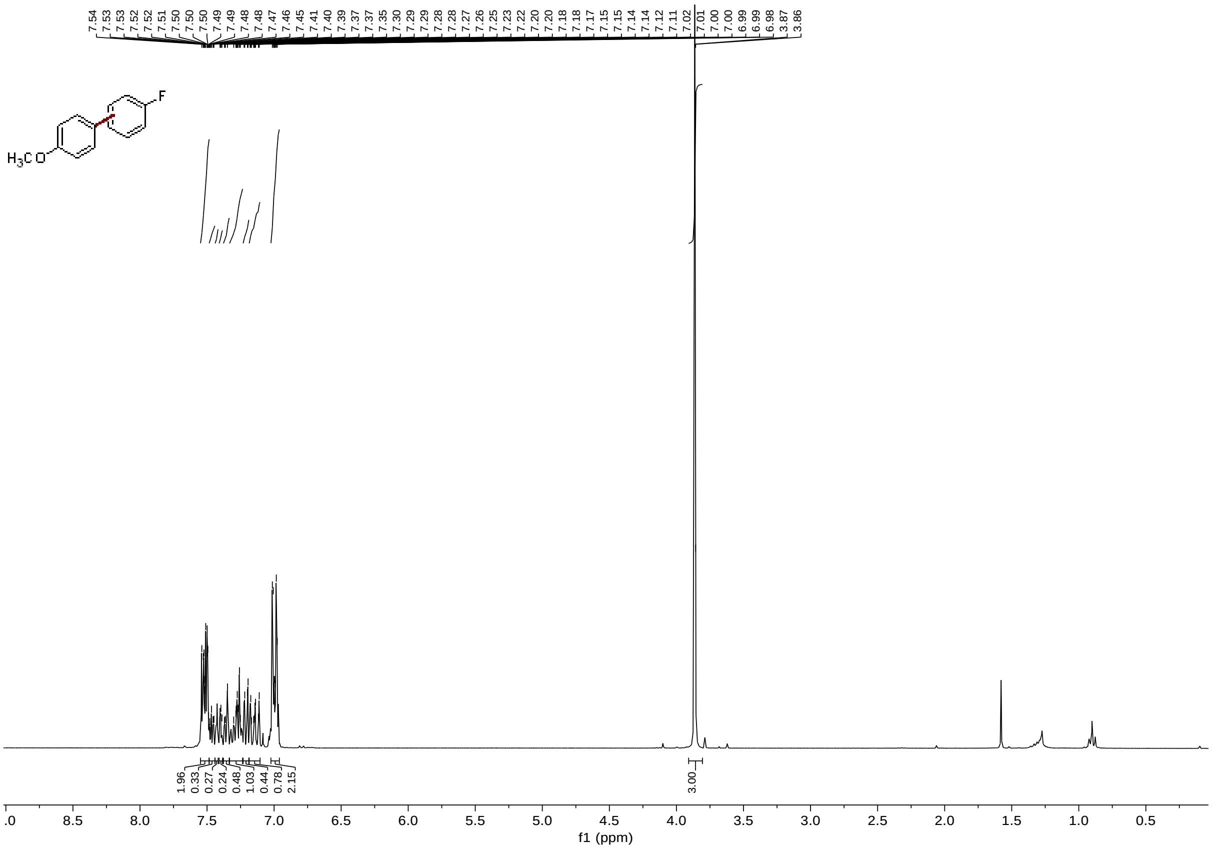

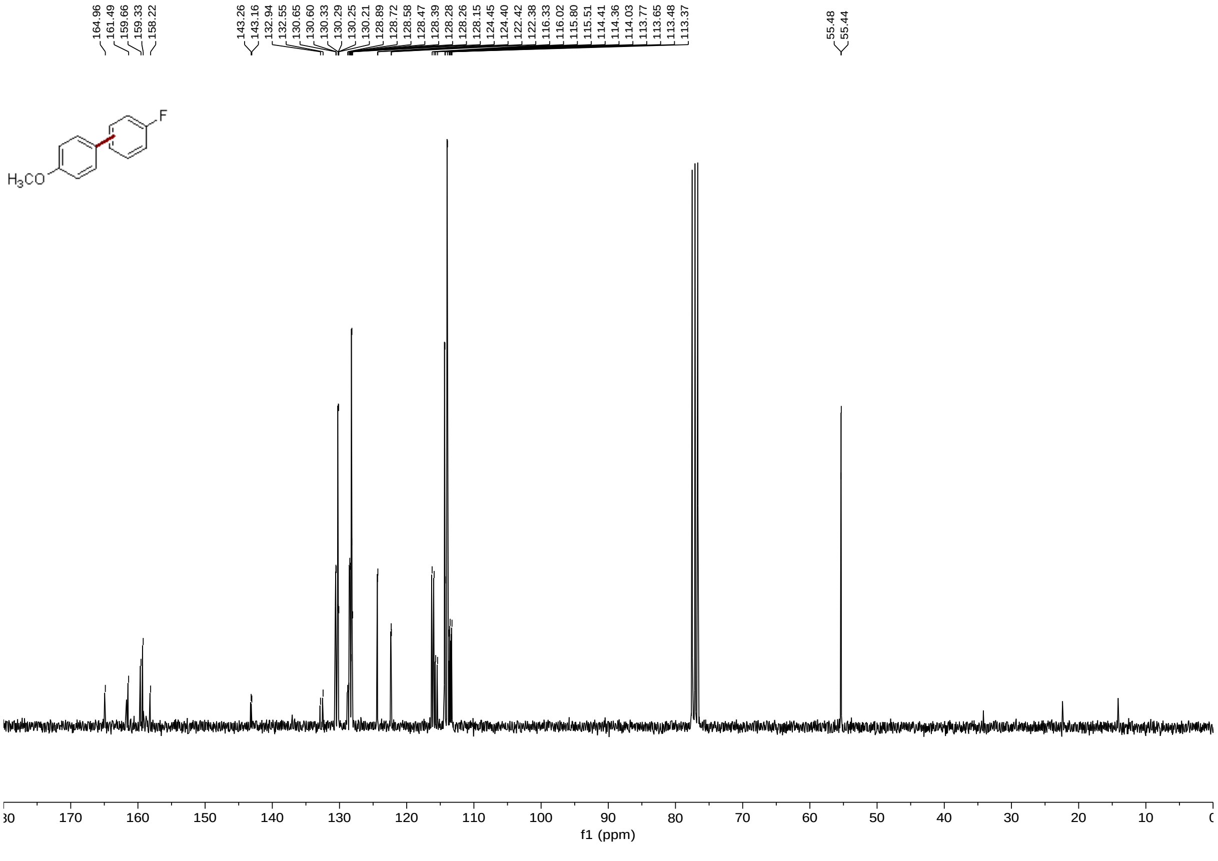

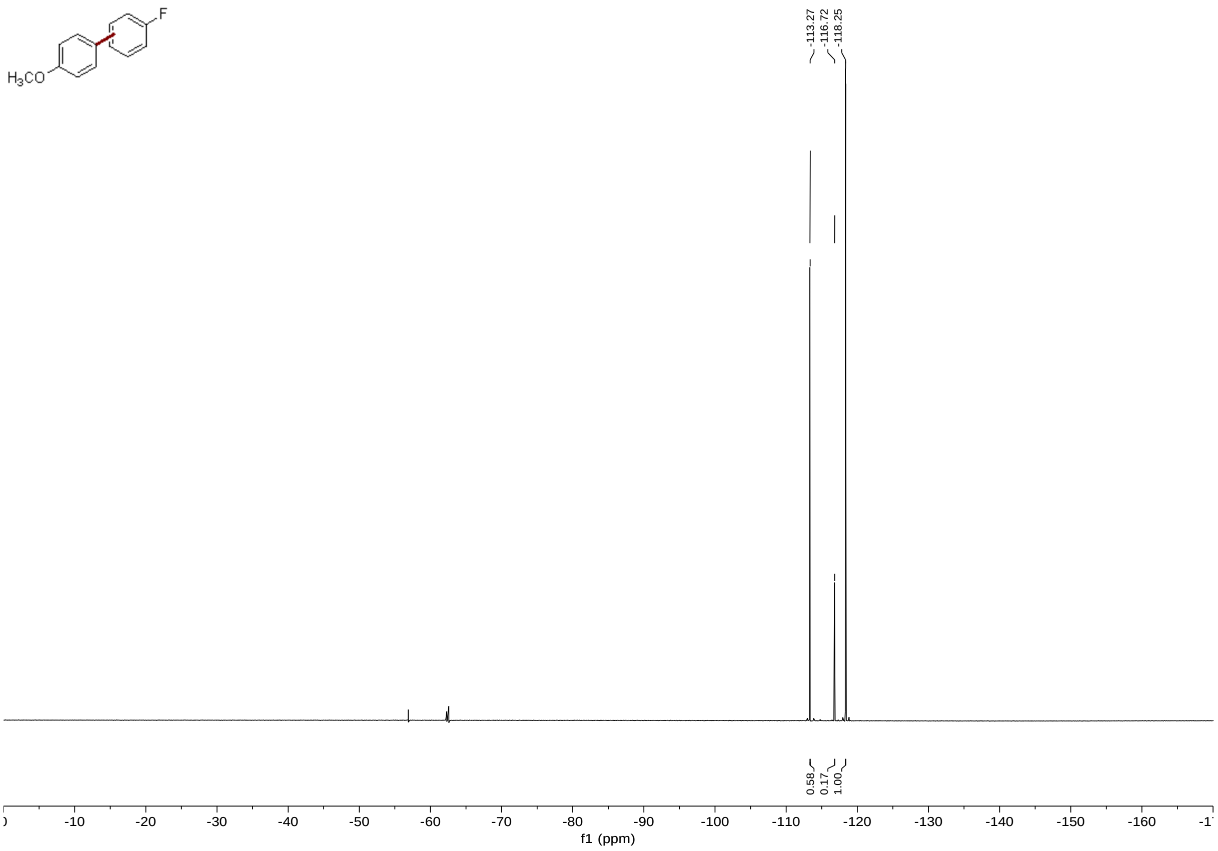

H3CO
F
-113.27
-116.72
-118.25
0.58
0.17
1.00
-10 -20 -30 -40 -50 -60 -70 -80 -90 -100 -110 -120 -130 -140 -150 -160
f1 (ppm)

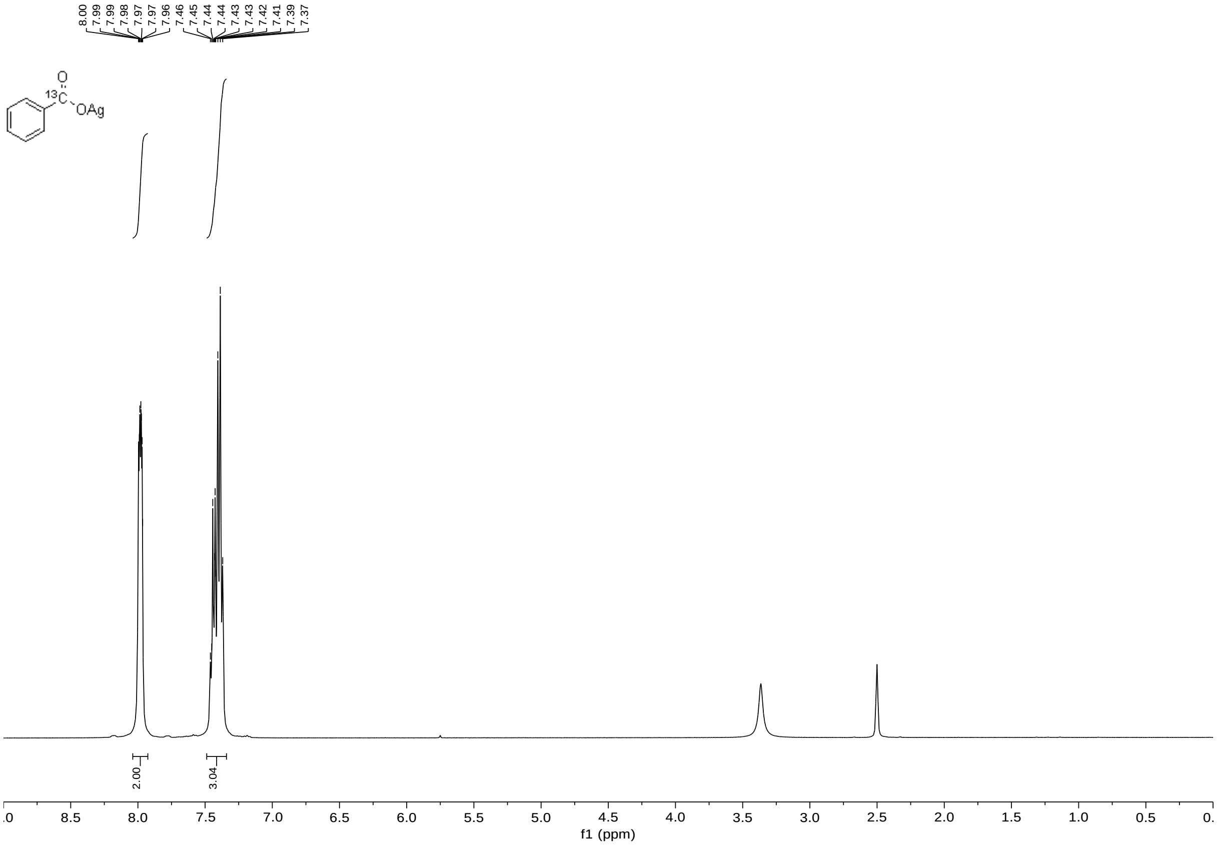

8.00 7.99 7.99 7.98 7.97 7.97 7.96 7.46 7.45 7.44 7.44 7.43 7.43 7.42 7.41 7.39 7.37
O
13C
OAg
2.00
3.04
8.5 8.0 7.5 7.0 6.5 6.0 5.5 5.0 4.5 4.0 3.5 3.0 2.5 2.0 1.5 1.0 0.5
f1 (ppm)

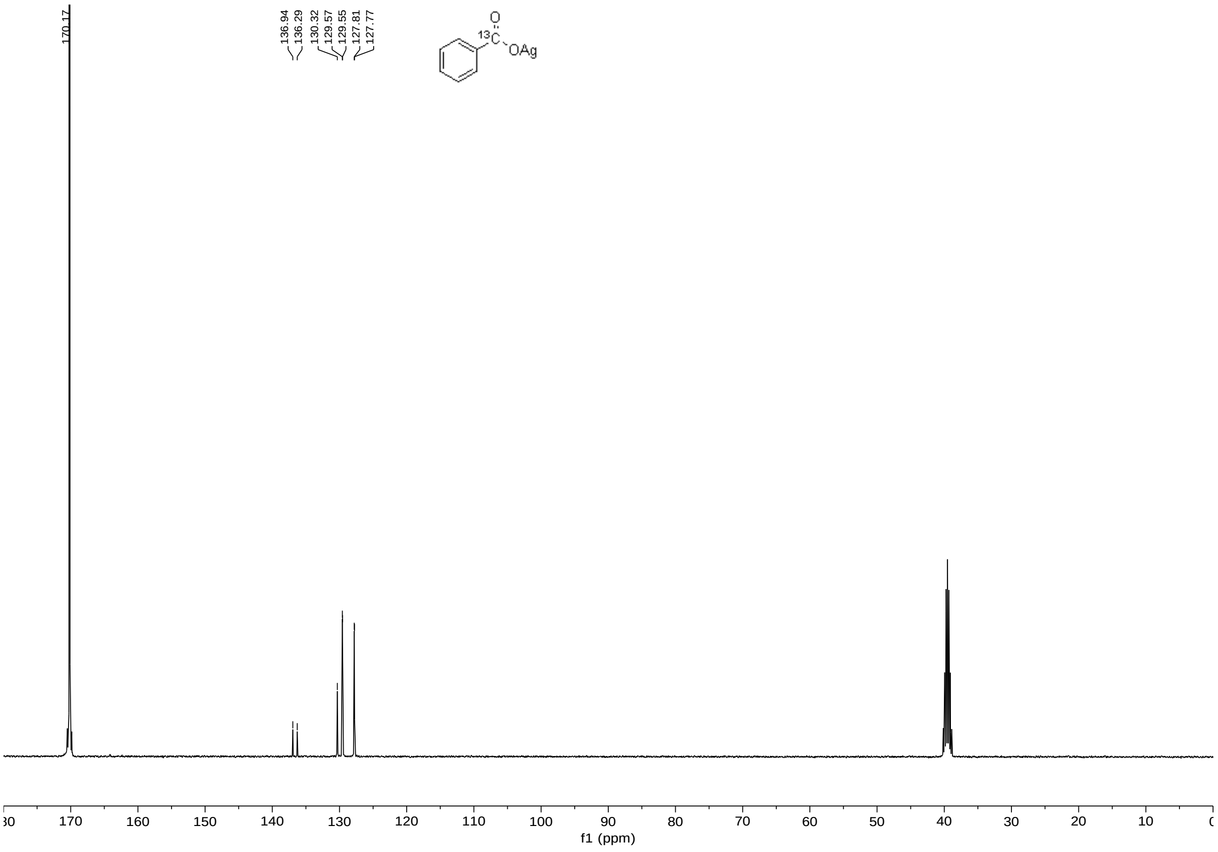
170.17
136.94
136.29
130.32
129.57
129.55
127.81
127.77
O
13C
OAg
f1 (ppm)

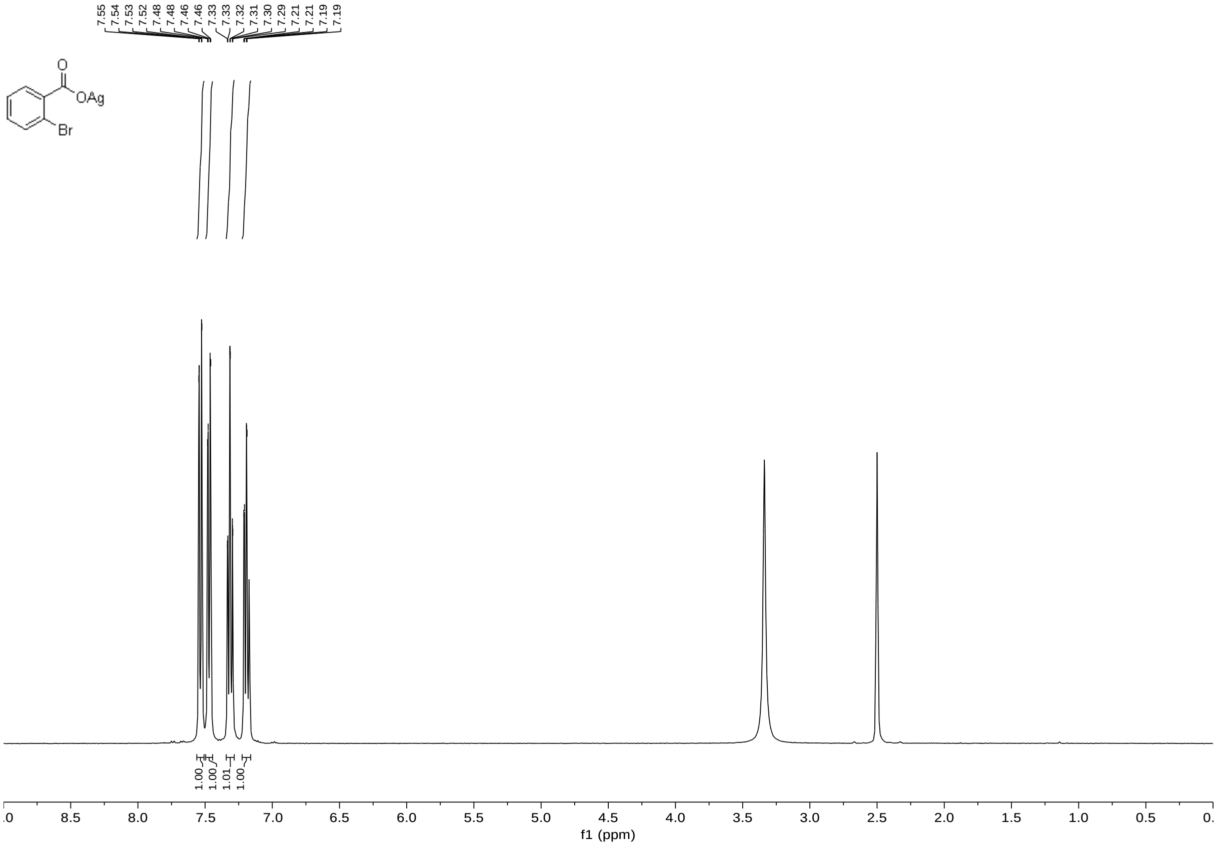
7.55
7.54
7.53
7.52
7.48
7.48
7.46
7.46
7.33
7.33
7.32
7.31
7.30
7.29
7.21
7.21
7.19
7.19
O
OAg
Br
1.00
1.00
1.01
1.00
f1 (ppm)

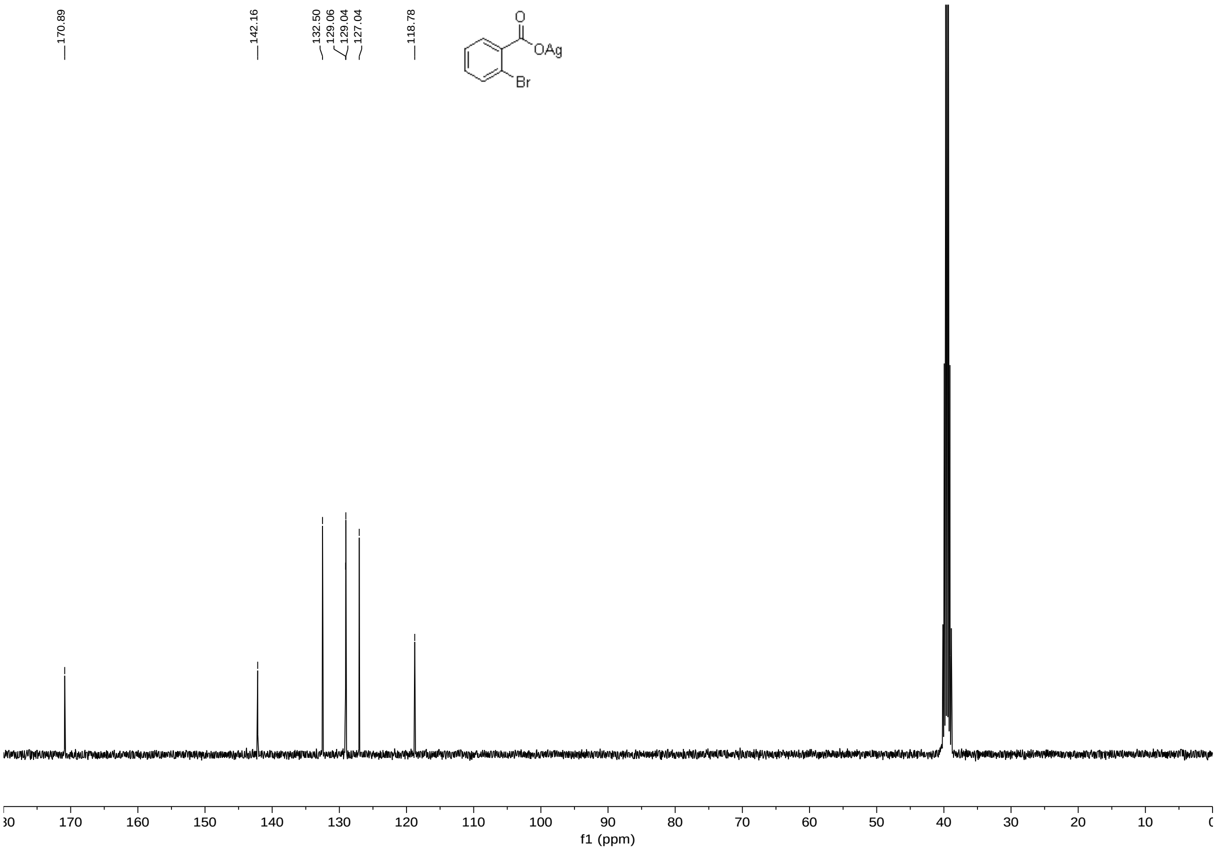

170.89
142.16
132.50
129.06
129.04
127.04
118.78
O
OAg
Br
170
160
150
140
130
120
110
100
90
80
70
60
50
40
30
20
10
f1 (ppm)
